# Recurrent tumor-specific regulation of alternative polyadenylation of cancer-related genes

**DOI:** 10.1186/s12864-018-4903-7

**Published:** 2018-07-13

**Authors:** Zhuyi Xue, René L. Warren, Ewan A. Gibb, Daniel MacMillan, Johnathan Wong, Readman Chiu, S. Austin Hammond, Chen Yang, Ka Ming Nip, Catherine A. Ennis, Abigail Hahn, Sheila Reynolds, Inanc Birol

**Affiliations:** 1 BC Cancer Agency, Genome Sciences Centre, Vancouver, BC V5Z 4S6 Canada; 2 Institute for Systems Biology, Seattle, 98109 WA USA; 3 Department of Medical Genetics, University of British Columbia, Vancouver, BC V6T 1Z3 Canada

**Keywords:** Alternative polyadenylation, Cancer, 3’ UTR, Cleavage site, RNA-Seq, de novo assembly, Trans-ABySS, The Cancer Genome Atlas, Cloud computing

## Abstract

**Background:**

Alternative polyadenylation (APA) results in messenger RNA molecules with different 3′ untranslated regions (3’ UTRs), affecting the molecules’ stability, localization, and translation. APA is pervasive and implicated in cancer. Earlier reports on APA focused on 3’ UTR length modifications and commonly characterized APA events as 3’ UTR shortening or lengthening. However, such characterization oversimplifies the processing of 3′ ends of transcripts and fails to adequately describe the various scenarios we observe.

**Results:**

We built a cloud-based targeted de novo transcript assembly and analysis pipeline that incorporates our previously developed cleavage site prediction tool, KLEAT. We applied this pipeline to elucidate the APA profiles of 114 genes in 9939 tumor and 729 tissue normal samples from The Cancer Genome Atlas (TCGA). The full set of 10,668 RNA-Seq samples from 33 cancer types has not been utilized by previous APA studies. By comparing the frequencies of predicted cleavage sites between normal and tumor sample groups, we identified 77 events (i.e. gene-cancer type pairs) of tumor-specific APA regulation in 13 cancer types; for 15 genes, such regulation is recurrent across multiple cancers. Our results also support a previous report showing the 3’ UTR shortening of *FGF2* in multiple cancers. However, over half of the events we identified display complex changes to 3’ UTR length that resist simple classification like shortening or lengthening.

**Conclusions:**

Recurrent tumor-specific regulation of APA is widespread in cancer. However, the regulation pattern that we observed in TCGA RNA-seq data cannot be described as straightforward 3’ UTR shortening or lengthening. Continued investigation into this complex, nuanced regulatory landscape will provide further insight into its role in tumor formation and development.

**Electronic supplementary material:**

The online version of this article (10.1186/s12864-018-4903-7) contains supplementary material, which is available to authorized users.

## Background

Alternative polyadenylation (APA) is a widespread regulatory mechanism that yields mRNAs with different 3′ untranslated regions (3’ UTRs) [1–5]. APA affects both normal cellular functions, such as proliferation and differentiation [6–8], and diseases [9, 10], including cancer [11–13]. For at least six genes, cancer cells favor mRNAs with shorter 3’ UTRs relative to normal cells; these mRNAs exhibit higher stability, potentially contributing to oncogenesis [11]. For most genes, however, mRNA stability may have a limited influence from 3’UTR isoforms [14].

APA is commonly characterized as length modulation of 3’ UTRs [6, 13, 15–17]. Shortening of 3’ UTR indicates that for a given gene, a transcript isoform with a shorter 3’ UTR is overexpressed relative to an isoform with a longer 3’ UTR given two conditions; lengthening of a 3’ UTR refers to the opposite scenario. Hence, APA is a form of differential isoform expression that pertains to the 3′ end. However, this paradigm is limited in scope as it considers only pairs of cleavage sites (CSs), commonly referred to as the proximal and distal CSs. Such characterization used to be sufficient when the number of annotated CSs was small (29,283 in human [18]), and most genes with APA had only two CSs [15]. However, with high-throughput sequencing methods, our understanding of the number of potential CSs a gene may have has increased significantly. For example, a study using a specialized sequencing protocol, PolyA-Seq, reported 439,390 CSs in human, where 49.3% of the genes have three or more CSs [19]. With more than two CSs, naming them proximal/distal is ambiguous and inadequate. In one case, only the most distal CS is called distal while all others are proximal [16]; in another case, proximal and distal CSs refer only to those from the top two most abundant isoforms [17]. Furthermore, 3’ UTR shortening/lengthening is relative and contextual. For example, when only the medium-length 3’ UTR is up- or down-regulated, neither shortening nor lengthening is appropriate.

While specialized high-throughput 3′ end sequencing protocols have been developed for CS profiling, they are not as widely adopted as RNA-Seq [13] and provide only limited transcriptomic data. Given that standard RNA-Seq libraries contain sufficient read evidence to identify APA events [13, 20, 21], the vast RNA-Seq datasets of The Cancer Genome Atlas (TCGA) have the potential to enable comprehensive APA analysis for both normal and tumor samples. A previous study on this topic introduced the DaPars model using a regression algorithm [13]. However, it imposes a fixed number of CSs for all genes, which is overly restrictive; in the reported results, it considered only two CSs, which is an oversimplification, as stated above. Furthermore, DaPars requires matched normal and tumor samples as input; thus, it effectively ignored the majority of tumor samples from TCGA since normal samples are highly underpopulated. In contrast, prediction tools like KLEAT [21] and ContextMap 2 [20] have no presumption of how many CSs a gene may have. They both leverage RNA-Seq reads with poly(A) tails. KLEAT uses de novo transcriptome assembly to identify contigs with poly(A) tails, which serve as high-confidence CS evidence [21]. Nonetheless, due to isoform overlap and complex mapping between CSs and stop codons, quantifying the isoform expression corresponding to the predicted CSs is still a challenge with RNA-Seq data.

Data analysis at the TCGA-scale is challenging. The download and storage alone can be a substantial undertaking. But with cloud computing, all data storage, transfer, and analysis could take place within a scalable cloud environment, avoiding most local storage cost and slow Internet communication. The cloud can provide thousands of CPUs in a short time for massively parallel processing, which could speed up large-scale analysis substantially.

Here, we built a cloud-based, targeted de novo transcript assembly pipeline that incorporates KLEAT, which we developed previously [21]. We executed the pipeline on the ISB Cancer Genomics Cloud, a cancer genomics cloud pilot based on the Google Cloud Platform (GCP, https://cloud.google.com/) [22], and predicted the CS profiles of 114 cancer-related genes for 10,668 RNA-Seq samples (totaling 67 TB in data size) in three days. Then, in the subsequent analysis on local servers, we applied a novel CS usage quantification approach by calculating the frequency per CS within a group of samples, leveraging the hundreds of samples available for each TCGA cancer cohort. By comparing the CS frequencies between normal and tumor sample groups, we identified widespread tumor-specific APA regulations that are recurrent across multiple cancer types. Over half of the identified events of tumor-specific regulation do not fall under the simplistic 3’UTR shortening and lengthening paradigm, but instead reveal a more intricate APA modulation of 3’UTRs in cancer.

## Results

### Cleavage site prediction on the cloud

**Fig. 1:**
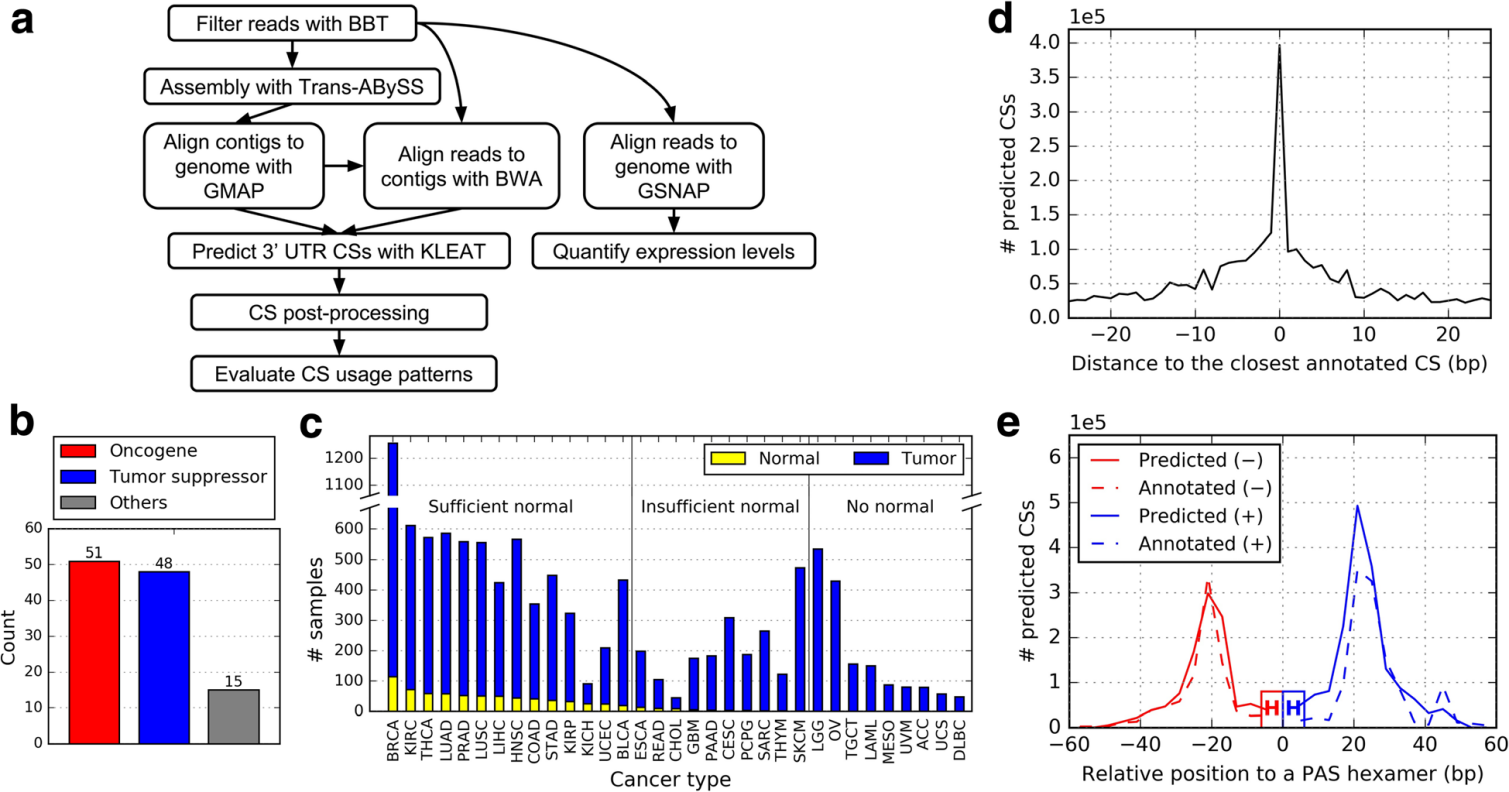
Cleavage site predictions. (**a**) Schematic diagram of the CS prediction pipeline. See Additional file 1: Figure S3A for a description of the CS post-processing step. (**b**) Count of gene types. (**c**) Count of TCGA RNA-Seq samples across 33 cancer types (sorted in decreasing order of normal and tumor samples). Sufficient normal: ≥15 samples. Alphabetically, ACC: adrenocortical carcinoma; BLCA: bladder urothelial carcinoma; BRCA: breast invasive carcinoma; CESC: cervical squamous cell carcinoma and endocervical adenocarcinoma; CHOL: cholangiocarcinoma; COAD: colon adenocarcinoma; DLBC: lymphoid neoplasm diffuse large B-cell lymphoma; ESCA: esophageal carcinoma; GBM: glioblastoma multiforme; HNSC: head and neck squamous cell carcinoma; KICH: kidney chromophobe; KIRC: kidney renal clear cell carcinoma; KIRP: kidney renal papillary cell carcinoma; LAML: acute myeloid leukemia; LGG: brain lower grade glioma; LIHC: liver hepatocellular carcinoma; LUAD: lung adenocarcinoma; LUSC: lung squamous cell carcinoma; MESO: mesothelioma; OV: ovarian serous cystadenocarcinoma; PAAD: pancreatic adenocarcinoma; PCPG: pheochromocytoma and paraganglioma; PRAD: prostate adenocarcinoma; READ: rectum adenocarcinoma; SARC: sarcoma; SKCM: skin cutaneous melanoma; STAD: stomach adenocarcinoma; TGCT: testicular germ cell tumors; THCA: thyroid carcinoma; THYM: thymoma; UCEC: uterine corpus endometrioid carcinoma; UCS: uterine carcinosarcoma; UVM: uveal melanoma. (**d**, **e**) Validation of our pipeline for predicting CSs. (**d**) Distribution of the distances between predicted and the closest annotated CSs. (**e**) Distribution of the distances between a predicted CS and the PAS hexamer motif found within 50 bp upstream. A high-resolution version of this figure is available for download in Additional file 5

To select a cleavage site (CS) prediction tool, we benchmarked DaPars [13], KLEAT [21] and ContextMap 2 [20] with a universal human reference RNA-Seq library (https://basespace.illumina.com/datacentral, dataset name: “HiSeq 2500: TruSeq Stranded mRNA LT (SEQC: UHR & Brain)”, sample ID: mRNA-UHRR-C1), and then compared their predictions to the CSs reported by PolyA-Seq, a data type also derived from the universal human reference and which served as the ground truth for tool evaluation [19] (Additional file 1: Figure S1). DaPars underperforms compared to the other two methods, most likely due to the limitation imposed by its two-CS model. ContextMap 2 has a limited sensitivity despite extensive parameter tuning, consistent with the authors’ own benchmark [20]. Hence, we used KLEAT to build a CS prediction pipeline (Fig. 1a).

We predicted the CS profiles of 114 cancer-related genes [11, 23] (Fig. 1b and Additional file 2: Table S1), in 9939 tumor and 729 normal TCGA paired-end RNA-Seq samples across 33 cancer types (Fig. 1c and Additional file 2: Table S2). To show the genes’ relatedness to cancer, we confirmed that all select genes have at least one pathogenic mutation (Additional file 1: Figure S2A), and they undergo fusion (F), mutation (M), overexpression (O) or underexpression (U) in different diseases (Additional file 1: Figure S2B), according to COSMIC v80 [24].

The pipeline was designed for the GCP, a scalable cloud computing environment, and packaged as a Docker (https://www.docker.com/) image for easy deployment and results reproducibility. We reached massive parallelism with a peak usage of over 3800 4-vCPU virtual machines running concurrently. In total, the samples consist of 1.6 trillion reads (53 bp average length) and amount to 67 TB of data after compression. We processed all samples in three days.

We filtered and clustered raw predictions from KLEAT to remove off-target and low-confidence CSs (Additional file 1: Figure S3A and Methods). Comparing the refined results to the Ensembl gene annotations (GRCh37.75) [25], we find that 66% of the predicted CSs are within 15 bp of an annotated site (Fig. 1d); 79% have a polyadenylation signal (PAS) hexamer motif within a 50 bp upstream window. Consistent with previous reports [26, 27], the distribution of distances between CSs and PAS hexamers peaks at around 21 bp (Fig. 1e and Additional file 1: Figure S3B), and the top two motifs are AATAAA (52%) and ATTAAA (13%) (Additional file 1: Figure S3C).

### Recurrent tumor-specific APA regulation

We quantified the usage of each predicted CS by calculating its frequency within a group of samples, leveraging the availability of tens or hundreds of normal and tumor samples per cancer type within TCGA. The usage frequency is defined as the fraction of normal/tumor samples within a cancer type predicted to use a given CS (Methods). We refer to all CS frequencies within one gene in a sample group as a cleavage pattern. When comparing a tumor cleavage pattern to a normal one, we identify an event (gene-cancer type pair) of tumor-specific APA regulation if one CS has a significantly higher frequency in tumor while another CS has a significantly lower frequency (*P* < 0.01 Fisher’s exact test, False Discovery Rate (FDR) < 0.002, Methods). Such analysis is applied to all gene-cancer type pairs available. In total, we identified 77 events that involve 33 genes across 13 cancer types. For 15 of these genes, the tumor-specific regulations are recurrent in multiple cancer types.

**Fig. 2:**
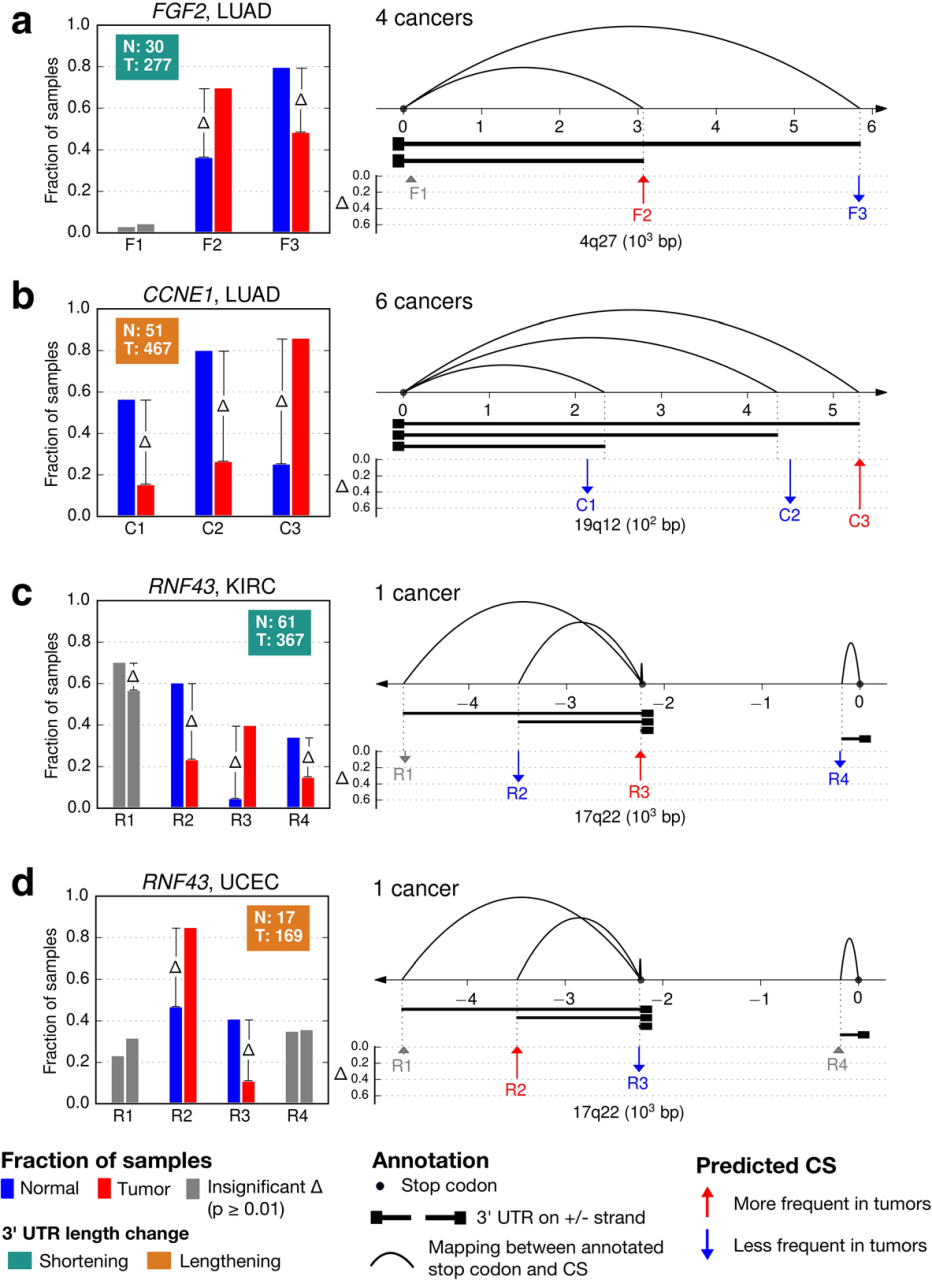
Selected events of tumor-specific APA regulations that indicate clear 3’ UTR length modulations in cancer. (**a**) *FGF2* in LUAD, a 3’ UTR shortening event. (**b**) *CCNE1* in LUAD, a 3’ UTR lengthening event. (**c**, **d**) *RNF43* in KIRC (3’ UTR shortening) and UCEC (lengthening). (**a**-**d**) Inside each left-hand panel, each group of bars represents the frequency of a specific CS in normal (blue) and tumor (red) samples. Bar groups are ordered by corresponding CS genomic coordinates. The text box shows the number of normal (N) and tumor (T) samples that were used for frequency calculation. The label box color indicates the trend of 3’ UTR length modulation in cancer. At the top, we indicate the number of cancer types with recurrent tumor-specific APA regulations. For example, “4 cancers” means that besides LUAD, tumor-specific APA regulation of *FGF2* is also observed in three other cancer types with consistent patterns (see text and Additional file 3: Figure S4 for details). Inside each right-hand panel, the diagram represents a depiction of the 3′ end region of each gene with 3’ UTR models directly below the genome axis. The axis direction (right/left) indicates the relative DNA strand (plus/minus); the axis coordinates are offset by that of the gene’s first stop codon. On the axis, arcs show the relationship between CSs and stop codons based solely on annotation. Below the axis, vertical arrows indicate the positions of predicted CSs. Annotated and predicted CSs match well, but they are not expected to overlap exactly. An arrow pointing upwards (downwards) represents an increase (decrease) in frequency from normal to tumor. Arrow height represents the difference (Δ) of the increase/decrease. Bars and arrows of insignificant difference are colored gray. For clarity, CSs with frequencies lower than 5% in both normal and tumor samples, and that do not undergo any significant change in any cancer type considered herein are not shown. For a comprehensive view of all CSs with distribution of gene expression levels, see Additional file 3: Figure S4. A high-resolution version of this figure is available for download in Additional file 5

**Fig. 3:**
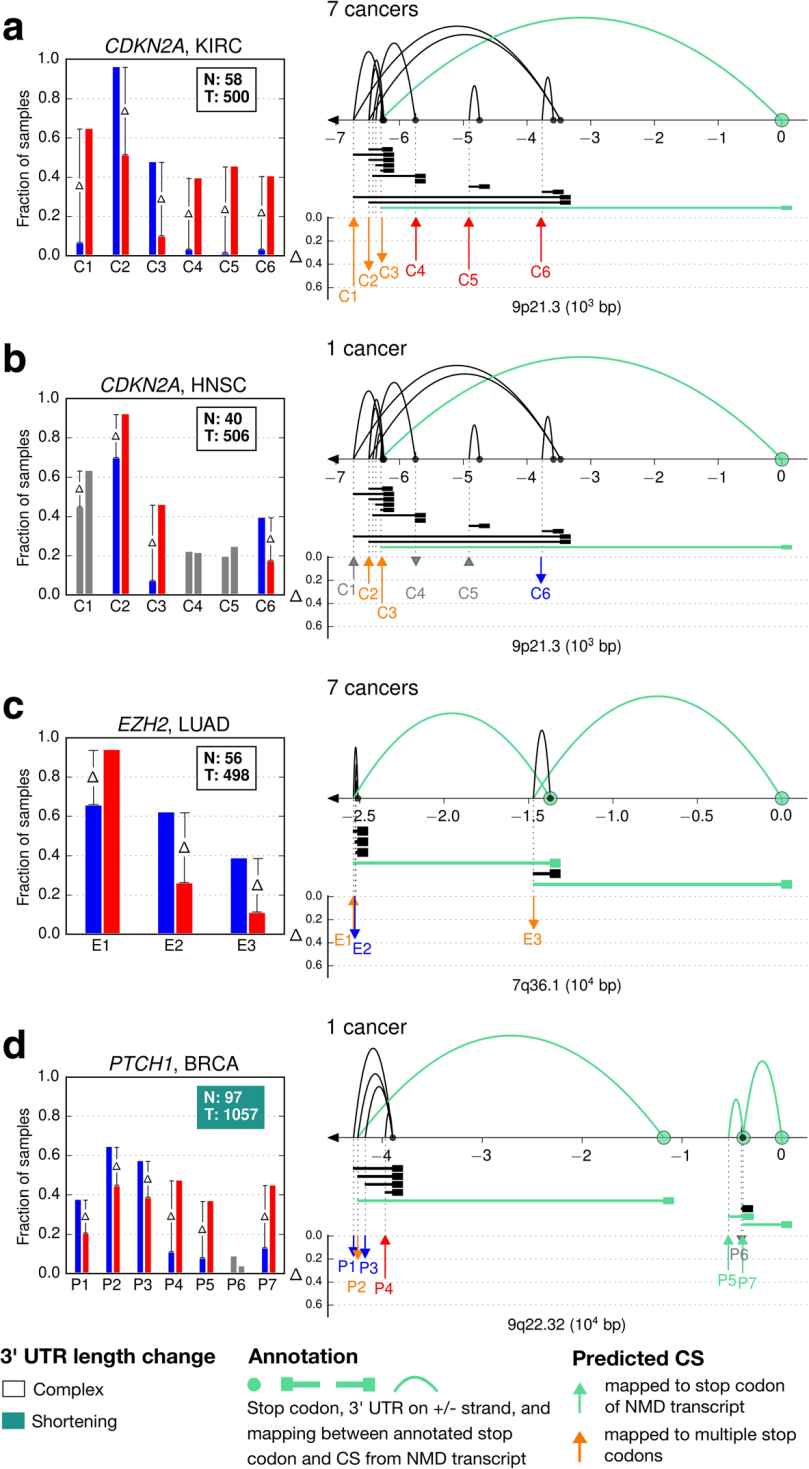
Selected events of tumor-specific APA regulations that do not fit the 3’ UTR length modulation paradigm. (**a**,**b**) *CDKN2A* in KIRC and HNSC. (**c**) *EZH2* in LUAD. (**d**) *PTCH1* in BRCA. (**a**-**d**) The legend of Fig. 2 applies. In addition, when the 3’ UTR length change is too complex to be resolved into a shortening or lengthening trend, the corresponding text box is left uncolored. NMD-related transcript elements are colored in cyan. An orange arrow indicates that a predicted CS with a significant frequency change is mapped to multiple stop codons, with its associated 3’ UTR length being ambiguous. A high-resolution version of this figure is available for download in Additional file 5

In our work, we highlight eight events. The first set of four events involves three genes, *FGF2*, *CCNE1*, *RNF43*, whose tumor-specific APA regulations indicate clear length modulations of the 3’ UTR in cancer (Fig. 2). The second set of four events involves the genes, *CDKN2A, EZH2*, and *PTCH1*, and show more complex modulations that do not fit the shortening/lengthening paradigm (Fig. 3). All other events are depicted in Additional file 3: Figure S4. Gene-level and CS-level summaries of all 77 events are provided in Additional file 2: Tables S3 and S4, respectively. In addition, we presented all 1596 (114 genes × 14 cancer types) gene-cancer pairs, including non-reported events, via an interactive web interface at http://bcgsc.ca/downloads/tasrkleat-static/off-cloud/results_data/all-apa-cases.html [28].

The *FGF2* gene (Fig. 2a) presents a 3’ UTR shortening event that has been reported previously in several cancer cell lines [11]. We label CSs by a letter from the gene name, followed by its relative index on the positive strand. *FGF2* is a positive strand gene with a single annotated stop codon, so an increment in the index (e.g. F2 to F3) indicates an increase in 3’ UTR length. In four TCGA cohorts (LUAD, BRCA, LUSC, and PRAD), the frequency of F2 increases in tumor samples, while that of F3 decreases, both significantly (*p* = 0.0004 and 0.001, respectively). We conclude that FGF2 undergoes 3’ UTR shortening in these cancers. The F1 site is over 2 kb from the closest annotated CS, and demonstrates the ability of our analysis to detect potential novel CSs. However, its usage frequency is low (typically less than 20%, Additional file 2: Table S4), and does not undergo significant change (*p* > 0.01, Fisher’s exact test) from normal to tumor; hence it is ignored for interpreting the 3’ UTR length modulation.

*CCNE1* (Fig. 2b) presents a 3’ UTR lengthening example in six cancer types (LUAD, BRCA, HNSC, KIRP, LIHC, and LUSC). It has three predicted CSs (C1, C2, C3) and a single annotated stop codon. C1 and C2 are associated with shorter 3’ UTR isoforms, and their respective frequency decreases, while the longer-3’UTR associated C3 increases in frequency in tumor samples, indicative of a 3’UTR lengthening in the aforementioned cancer cohorts.

In contrast to *FGF2* and *CCNE1*, the *RNF43* gene (Fig. 2c, d) has two annotated stop codons. It shows both 3’ UTR shortening and lengthening, depending on the tissue of origin. *RNF43* has four predicted CSs. Since it is a negative-strand gene, an increment in the index indicates a decrease in 3’ UTR length, for CSs that share the same stop codon (R1, R2, and R3). Among them, R2 and R3 undergo significant shifts in their frequencies from normal to tumor in both KIRC and UCEC. However, these shifts occur in opposite directions, indicating 3’ UTR shortening in KIRC, but 3’ UTR lengthening in UCEC. As for R4, which is associated with a different stop codon, its frequency decreases significantly in KIRC, indicating decreased expression of its corresponding isoforms in KIRC tumor.

Unlike the above events that can be characterized as 3’ UTR shortening or lengthening, over half of the identified events of tumor-specific APA regulation indicate more complex modulation to 3’ UTRs. The *CDKN2A* gene, like *RNF43*, also displays multiple disease-dependent frequency shifts in KIRC and HNSC (C2, C3, and C6, Fig. 3a,b). However, *CDKN2A* is much more complex because it has seven annotated stop codons, and some of its CSs could exhibit one-to-many relationship to certain stop codons (C1, C2, and C3), while others have a one-to-one relationship to separate stop codons (e.g. C4, C5, and C6) (Fig. 3, arcs plots). One-to-many relationships blur the 3’UTR length assignment for the involved CS; correspondence to separate stop codons confounds the length comparison due to limited or no overlap among the involved 3’ UTRs. In addition, one stop codon (matched to C3) belongs to a transcript involved in nonsense mediated decay (NMD), which is a surveillance mechanism for removing prematurely transcribed mRNAs [29, 30]. NMD transcripts have longer 3’ UTRs than protein coding transcripts (*P* = 6.5 × 10^− 16^, Kolmogorov–Smirnov test) (Additional file 1: Figure S5), which could facilitate its detection by the decay machinery [31]. We observe the implication of NMD to be common (Figs. 3 and Additional file 3: Figure S4), which adds further complexity to the interpretation of APA. The intricate pattern of tumor-specific APA regulation of C*DKN2A* in KIRC is also identified in COAD, KICH, KIRP, LIHC, PRAD and THCA, but describing such regulation by 3’ UTR length modulation would be inadequate.

Much like *CDKN2A*, the *EZH2* gene (Fig. 3c) displays a recurring regulation in seven cancer types (BRCA, KIRC, KIRP, LIHC, LUAD, PRAD, THCA). Besides, it illustrates another level of complexity, as its second stop codon (near E3) is shared by both protein coding and NMD transcripts. Thus, *EZH2* presents a many-to-many-to-many relationship among CSs, stop codons, and transcript types.

Finally, we show *PTCH1* in BRCA **(**Fig. 3d**).** Despite complex mappings between CSs and stop codons, this event can be characterized as 3’ UTR shortening. Ignoring CSs that were mapped either to a separate stop codon (P5, P6, and P7), or to multiple stop codons (P2), we are left with three CSs. Among these, P4 (shorter 3’ UTR) increases in frequency, while P1 and P3 (longer 3’ UTRs) decrease in frequency in tumor samples, implying 3’ UTR shortening.

**Fig. 4:**
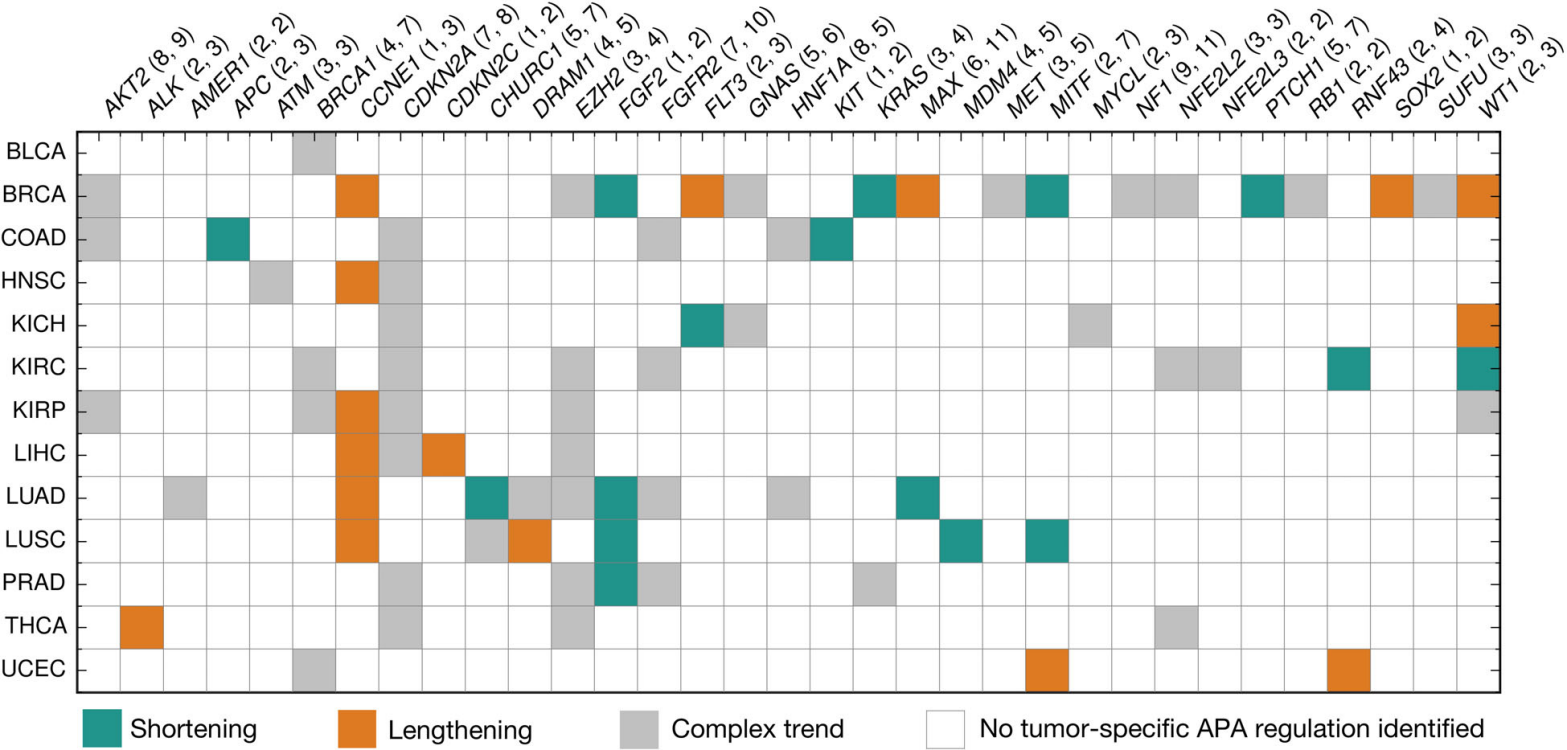
Trends of 3’ UTR length modulation across all 77 tumor-specific APA events. The numbers of annotated stop codons and CSs per gene are shown in parentheses. For example, *AKT2* (8, 9) means the gene has eight annotated stop codons and nine annotated CSs

For all identified 77 events of tumor-specific APA regulation, we identified equal numbers (16) of 3’ UTR shortening and lengthening events, and we labeled the remaining 45 events as having complex trends (Fig. 4).

## Discussion

The current paradigm of APA characterization revolves around analyzing two CSs at a time, namely proximal and distal CSs, and emphasizes 3’ UTR length change, namely shortening or lengthening [15]. However, at the age of high-throughput sequencing, we now know that the majority of human genes may have more than two CSs [19], and considering only two CSs needlessly limits the scope of APA analysis. Our analysis considered all known CSs and 3’ UTRs (tandem, overlapping, mutually exclusive or NMD) for a list of cancer-related genes. We found that 40% (31/77) of the reported APA events have three or more CSs undergoing significant frequency changes (*p* < 0.01, Fisher’s exact test). While we also report 3’ UTR shortening and lengthening events, including the independent identification of a previously published observation (*FGF2*) [11], over half of our identified events of APA regulation deviate from simple 3’ UTR length modulations. The deviation is due to the inherent complexity of APA analysis, which will be discussed below, and it suggests that 3’ UTR length alone may not be a major factor in its function, consistent with a previous study that reported the limited effects of various 3’ UTR isoforms on mRNA stability [14].

While a CS is essentially a single point (after clustering) on the genome axis, and quantifying its frequency is relatively straightforward, the analysis quickly becomes complex once we considered their corresponding 3’ UTRs. The complexity of APA analysis is multifold. First, in the 77 events of tumor-specific APA regulation reported here, 33/297 (11%) CSs are mapped to more than one stop codon. In such a multi-mapping situation, CS frequency cannot represent the usage of any of the corresponding isoforms, neither can an expression-level type of quantification from a specialized 3′ end sequencing protocols [16, 19, 32, 33]. Instead, it only provides an aggregate measure of usage for all the 3’ UTRs cleaved at the same CS. Second, when the CS-to-stop codon mappings are one-to-one, the interpretation of 3’ UTR length modification can still be difficult, especially when the 3’ UTRs are associated with different stop codons and have limited or no overlap (e.g. *CDKN2A*). In addition, 16/33 of the reported genes have stop codons related to NMD transcripts; in some genes (e.g. *EZH2*), a stop codon can be used in both protein coding and NMD transcripts, which complicates the implications of regulation by APA even more. Describing APA regulation by 3’ UTR length change is mostly inadequate, and comprehensive characterization will require techniques that can disambiguate the relationship between a gene’s 3’ UTRs and CS repertoire. At a deeper level, APA is a partial view of the differential expression of isoforms at the 3′ end.

We recognize that, despite its limitations, the old paradigm of 3’ UTR shortening and lengthening is sufficient within specific contexts. For example, *CD47* has only two CSs and one stop codon, and its two APA isoforms have identical upstream sequence compositions; thus, proximal and distal CSs, short and long 3’ UTRs all have concrete meanings. Remarkably, the CD47 protein translated from the long isoform is relocated to the cell membrane while the protein corresponding to the short isoform remains in the endoplasmic reticulum [34].

Our APA analysis on RNA-Seq data is analogous to that using PAS-Seq [32] and 3’READS [33] data. While these studies both used read count per CS (vertical measure) per sample, we used frequency per sample group (horizontal measure), leveraging the large number of samples available within TCGA. All three studies used Fisher’s exact test to identify significance of APA regulation. However, our analysis considers all CSs instead of only proximal and distal ones.

To report an event (gene-cancer type pair) of tumor-specific APA regulation, we enforced a rather stringent requirement; the co-occurrence of at least one significant increase (*p* < 0.01, Fisher’s exact test) and one significant decrease in the frequencies of two CSs of a given gene. This requirement was designed to minimize the influence of gene expression variation on the comparison of CS frequencies. Without this requirement, an increase or decrease in the CS frequency could simply be a result of gene up- or down-regulation in the normal or tumor samples. For example, if a gene is down-regulated in tumor, its corresponding CSs will be observed less frequently. Conversely, if there is an increase in the usage of at least one other CS, it implies the involvement of APA regulation mechanisms [1–4]. While such stringency brings down the FDR (< 0.002), it also reduces sensitivity by excluding potential APA events, consistent with an earlier report [11] (e.g. *CCND2* in COAD, HNSC, KIRC, KIRP, LUSC, and THCA; *DICER1* in BRCA, LUSC, and STAD; *RAB10* in BLCA, BRCA, COAD, LUSC, STAD, and UCEC, data shown at the aforementioned URL [28]).

Of the 33 genes reported herein, 18 show tumor-specific APA regulation in a single cancer. We propose two potential reasons for the lack of recurrence for these events. First, a gene undergoing tumor-specific APA regulation in one cancer may not do so in another. This includes cases where APA regulation in multiple cancer types may be following different patterns, as we have highlighted for *RNF43*. Second, we expect the requirement of concurrent increase/decrease in CS frequencies to reduce our sensitivity in detecting APA regulation events. As a result, some tumor-specific APA regulation events may indeed be recurrent in other cancer types, but below the detection limit of our approach.

This work presents the results of a targeted analysis. The 114 genes we selected are not only cancer-related, but also display a range of 3′-end patterns from simple (e.g. *FGF2*) to complex (e.g. *CDKN2A*); thus, they are suitable for studying tumor-specific APA and demonstrating the complexities of APA regulation in human cancers. We acknowledge their limitation in showing genome-wide tumor-specific APA regulations. Still, the genes inspected in our study reveal that APA regulation in cancer is more complex than previously thought. Despite our stringent requirements for reporting a tumor-specific APA regulation event, a sizable proportion of APA genes (33/114) were identified, and about half of them (15/33) show recurrent tumor-specific APA regulation across multiple cancers. Therefore, we think that the specific events reported herein represent a wider phenomena, and that many more additional events of tumor-specific APA regulation remain to be discovered.

## Conclusions

We identified widespread recurrent tumor-specific APA regulation across multiple TCGA cancers, using standard RNA-Seq data. We observed a wide spread complex APA regulatory regime, with many genes using multiple CSs. This new perspective demands a specialized vocabulary to describe APA, as the conventional paradigm of 3’ UTR shortening/lengthening is insufficient to describe these observations. Further understanding of this complex process would also yield insight into the potential functional consequences of APA in normal and disease states.

## Methods

### RNA-Seq data

We used a copy of the TCGA RNA-Seq data hosted by the Institute for Systems Biology-Cancer Genomics Cloud (ISB-CGC) pilot on the Google Cloud Storage, part of the Google Cloud Platform (GCP), mirroring the repository hosted at the NCI Genomic Data Commons (GDC, https://gdc.cancer.gov/). In total, 10,668 samples were analyzed, with each sample identified by a unique analysis ID. Sample types are generalized as ‘normal’ (solid tissue normal) and ‘tumor’ (which includes primary solid tumor, metastatic, recurrent solid tumor, additional - new primary). A more detailed description of the protocols for data collection is provided in Additional file 4: Supplementary Methods.

### Design of the targeted CS prediction pipeline

RNA-Seq reads were first filtered against the candidate genes using the biobloomcategorizer utility from BioBloomTools (BBT) [35]. The resulting categorized reads were then assembled into contigs with Trans-ABySS [36], and these contigs were in turn aligned to the reference human genome with GMAP [37]. The raw reads were aligned to both the assembled contigs with BWA [38], and the reference genome with GSNAP [39]. Both contig-to-genome and read-to-contig alignment results were used to predict CSs with KLEAT [21], and the read-to-genome alignments were used for both expression level quantification and assessment of KLEAT predictions.

### Implementation of the pipeline

The pipeline was implemented in Python with the Ruffus framework [40]. The software used include SAMtools-0.1.18 [41], BioBloom tools-2.0.12 [35], Trans-ABySS-1.5.2 [36], ABySS-1.5.2 [42], GMAP-2014-12-28 [37, 39], and BWA-0.7.12 [38]. The source code also includes a copy of the specific version of KLEAT.py used in this study. For its use on the GCP, a Docker image of the pipeline can be built from the Dockerfile included in the source code.

### Execution of the pipeline

We executed the pipeline on the ISB-CGC powered by the GCP. For each RNA-Seq sample, a virtual machine (VM) instance with four vCPUs, 20 GB of memory, and a sample size-dependent amount of persistent disk was used. For each instance, we requested a sufficient disk size for storing both input data and intermediate and final results, calculated as 30 x Size(sample) + 50 GB. The scaling factor of 30 is based on experience in pilot runs, and the extra 50 GB was reserved for storing reference data. Google Genomics Pipelines API (https://cloud.google.com/genomics/reference/rest/v1alpha2/pipelines) was used to orchestrate all VM instance tasks including VM creation, deletion, and data transfer, and it substantially reduced the administrative workload.

The reference data included an hg19 reference genome [43], the GMAP/GSNAP [37, 39] index of hg19, a prebuilt BioBloom filter [35] of all the candidate genes’ transcripts, and a specific version of the gene annotation used by KLEAT [21] (more details on annotation are available in Additional file 4: Supplementary Methods).

The BioBloom filter was built with the biobloommaker utility from BBT [35]. As for the input to biobloommaker, all transcripts of all the candidate genes from the Ensembl annotation [25] were used. The annotated sequences were augmented by 300 bp flanking sequences on both ends of each transcript to collect RNA-Seq reads that were partially aligned to them.

During the de novo assembly of transcripts for each sample, three *k*-mer sizes were used, depending on the corresponding read length: {22, 32, 42}, {32, 52, 72}, and {32, 62, 92} were used for samples with read lengths of 45–50, 75–76, and 100 bp, respectively.

### Annotation pre-processing

The Ensembl annotation was downloaded from http://ftp.ensembl.org/pub/release-75/gtf/homo_sapiens/Homo_sapiens.GRCh37.75.gtf.gz, and then pre-processed before being used. First, we extracted the annotated CSs of all protein coding and NMD transcripts that were CDS 3′ complete (without cds_end_NF tag, https://www.gencodegenes.org/gencode_tags.html) for all candidate genes. To calculate 3’ UTR lengths, we also extracted the mapping information between annotated CSs and stop codons from transcripts. A more detailed description of the extraction process can be found in Additional file 4: Supplementary Methods. After extraction, since a predicted CS may not have transcript-level resolution when associated with multiple transcripts, we discarded transcript-level information from the annotation, and removed redundant mapping relationships caused by multiple transcripts sharing the same CS and stop codon. Lastly, we clustered the annotated CSs as described in CS Clustering below.

### CS prediction and post-processing

The CSs were predicted by KLEAT with all parameters set to default. We post-processed the KLEAT results before any CS usage frequency analysis (Additional file 1: Figure S3A). Specifically, we parsed the 10,668 KLEAT output files (one per sample), using the information from the following fields: *gene*, *transcript_strand*, *chromosome*, *cleavage_site*, *length_of_tail_in_contig*, *number_of_bridge_reads*, and *max_bridge_read_tail_length*. In total, 67,544,140 CSs were predicted across 10,668 samples. First, we filtered out off-target CSs by only selecting those that were associated with the candidate genes, keeping 17% of the predictions. After initial filtering by genes, we reassigned each remaining CS to the closest clustered annotated CS (See Annotation pre-processing), and then calculated the signed distance between them. We also calculated the location of PAS hexamer motifs, if present, searching up to a 50 bp window upstream of a predicted CS. When multiple PAS hexamers existed in the window, the strongest one was picked [26]. Next, we applied another filter to select the most confident predictions. Specifically, a predicted CS must meet at least one of the following two criteria to be retained:

1. Its distance to the closest annotated CS was required to be 25 bp or less. The 25-bp threshold was chosen by plotting the distribution of distances, and taking a threshold at the plateau. This criterion was designed for selecting CSs that had already been annotated.
2. One of the two strongest PAS hexamers AATAAA and ATTAAA [27] were required to be within a 50 bp window, and at least one of the following conditions of polyadenylation evidence was satisfied: length_of_tail_in_contig ≥4, number_of_bridge_reads ≥2, or max_bridge_read_tail_length ≥ 4. The second criterion is an empirical one that is independent of annotation, and it is designed mainly for selecting potential novel CSs.

We verified that AATAAA and ATTAAA were the two most frequent PAS hexamers associated with the predicted CSs both before and after the second filtering steps (Additional file 1: Figure S3C). After the two filtering steps, about 5% of the CSs were retained and clustered as described in the CS Clustering section. The CSs filtered out by this process are considered not robust enough and thus omitted from further analysis to reduce false positives. We also confirm that there is no gene overlap among the 114 genes investigated here. The post-processing steps resulted in 2136 unique predicted CSs in 114 candidate genes across all samples.

### CS clustering

We used the single-linkage hierarchical clustering algorithm to combine CSs that were ≤ 20 bp apart, iterating when necessary for clusters to converge. After clustering, we selected the mode CS coordinate within each cluster as its representative location. If multiple modes existed, the median of the modes was used. Then, every CS was associated with one of the representative CSs, and multiple CSs associated with the same representative CS were merged within each sample. The clustering method was independently applied to both annotated and predicted CSs.

The clustering process inevitably decreases the prediction resolution, so our analysis is not able to distinguish CSs that were closer than the clustering cutoff (20 bp). However, we verified that the clustering results were insensitive to different cutoff values, even though the number of clusters could vary.

### CS usage frequency calculation

For a given CS in each gene, each cancer type and each sample type (normal/tumor), its frequency is calculated as the fraction of samples that were predicted to use it:

$$ freq=\frac{s}{g} $$

where *s* is the number of samples predicted to use the CS, and *g* is the total number of samples with sufficient gene expression level of this gene available for this cancer type and sample type. For each sample, the gene expression level is considered sufficient if at least one CS was predicted within the gene; otherwise, the expression was considered insufficient, and the sample was excluded from the frequency calculation.

### Comparison of cleavage patterns between normal and tumor samples

For every predicted CS of every gene in each cancer type, we calculated its frequencies in both normal and tumor samples, and then evaluated the significance of the difference with a Fisher’s exact test. The input to the test included the number of normal and tumor samples with and without a CS predicted. The frequencies of multiple CSs within one gene collectively formed a cleavage pattern for that gene, and to report the difference in patterns between normal and tumor, we required the co-occurrence of at least one significant increase (*p* < 0.01) and one significant decrease in the frequencies of two CSs, respectively.

To estimate the false discovery rate (FDR), we obtained an upper bound for the *p*-value at the gene-cancer pair level by multiplying the lowest *p*-values of its corresponding significant (p < 0.01) increase and decrease in CS frequency. Thus, pair-level p-values are less than 0.0001 (0.01 × 0.01). For gene-cancer type pairs that are not reported, we assigned an arbitrary p-value of 1. In total 1596 hypothesis tests (114 genes × 14 cancer types) were conducted, and applying the Benjamini-Hochberg procedure [44], we obtain an FDR < 0.002. Note that our FDR calculation is conservative since we only considered two CSs when estimating the pair-level p-values while 40% (31/77) of the reported APA events had three or more CSs undergoing significant frequency changes.

### Resolution of the 3’ UTR length change trends

We first mapped a predicted CS to the closest annotated one. If it was > 25 bp away, the predicted CS was considered potentially novel, and was ignored for length trend resolution because of the uncertainty of its corresponding stop codon. After trying a range of values, the 25-bp cutoff was selected when the number of unmapped CSs reached a plateau.

After mapping we determined the associated stop codons for each CS, also based on annotation. We do not assume that a CS could be associated with all upstream stop codons, in accordance with the transcript annotations, which do not support an all-to-all type of relationship (arcs in Figs. 2, 3 and Additional file 3: Figure S4). If a CS was associated with only a single stop codon, its corresponding 3’ UTR length was unambiguously calculated and used for trend resolution. All CSs mapped to multiple stop codons were ignored. A detailed description of the trend resolution approach is provided in Additional file 4: Supplementary Methods.

### Python libraries used

In addition to the aforementioned Ruffus framework [40], we used several other Python libraries for scientific computing [45] to facilitate our analysis. The hierarchical clustering algorithm implemented in SciPy-0.18.1 [46, 47] was used for CS clustering. Pandas-0.19.0 [48] was used for tabular data transformation and analysis. Matplotlib-1.5.3 [49] was used for plotting. Jupyter-1.0.0 notebook [50] was used for tracking analysis steps and results.

## Additional files

Additional file 1: Supplementary figures. It includes all supplementary figures except **Figure S4.** **Figure S1.** Benchmark of DaPars, KLEAT and ContextMap 2. **Figure S2.** Relevance of 114 select genes to cancer according to COSMIC. **Figure S3.** Detail of CS predictions. **Figure S5.** Distribution of 3’ UTR lengths of protein coding and NMD transcripts. (PDF 2210 kb)

Additional file 2: Supplementary tables. It includes all supplementary tables. **Table S1.**List of 114 cancer-related genes. **Table S2.** Summary of the 33 cancer types. **Table S3.** Summary of the reported 33 genes involved in 77 events of tumor-specific cleavage patterns. **Table S4.** Details of all CSs involved in the reported 77 APA events. **Table S5.** Mapping relations between diseases in TCGA and those in COSMIC (Supplementary Methods). (XLSX 235 kb)

Additional file 3: **Figure S4.** Illustration of all 77 identified events of tumor-specific cleavage patterns. (PDF 5699 kb)

Additional file 4: Supplementary methods. Description of additional methods. (PDF 157 kb)

Additional file 5: Figures available for download. (PDF 86 kb)

## Abbreviations

3’ UTR: 3′ untranslated region
ACC: Adrenocortical carcinoma
APA: Alternative polyadenylation
BBT: BioBloomTools
BLCA: Bladder urothelial carcinoma
BRCA: Breast invasive carcinoma
CESC: Cervical squamous cell carcinoma and endocervical adenocarcinoma
CHOL: Cholangiocarcinoma
COAD: Colon adenocarcinoma
COSMIC: Catalogue of Somatic Mutations in Cancer
CS: Cleavage site
DLBC: Lymphoid neoplasm diffuse large B-cell lymphoma
ESCA: Esophageal carcinoma
FDR: False discovery rate
GBM: Glioblastoma multiforme
GCP: Google Cloud Platform
GCS: Google Cloud Storage
GDC: NCI Genomic Data Commons
GRC: Genome Reference Consortium
HNSC: Head and neck squamous cell carcinoma
ISB-CGC: Institute for Systems Biology-Cancer Genomics Cloud
KICH: Kidney chromophobe
KIRC: Kidney renal clear cell carcinoma
KIRP: Kidney renal papillary cell carcinoma
LAML: Acute myeloid leukemia
LGG: Brain lower grade glioma
LIHC: Liver hepatocellular carcinoma
LUAD: Lung adenocarcinoma
LUSC: Lung squamous cell carcinoma
MESO: Mesothelioma
NMD: Nonsense-mediated mRNA decay
OV: Ovarian serous cystadenocarcinoma
PAAD: Pancreatic adenocarcinoma
PAS: Polyadenylation signal
PCPG: Pheochromocytoma and paraganglioma
PRAD: Prostate adenocarcinoma
READ: Rectum adenocarcinoma
SARC: Sarcoma
SKCM: Skin cutaneous melanoma
STAD: Stomach adenocarcinoma
TCGA: The Cancer Genome Atlas
TGCT: Testicular germ cell tumors
THCA: Thyroid carcinoma
THYM: Thymoma
UCEC: Uterine corpus endometrioid carcinoma
UCS: Uterine carcinosarcoma
UVM: Uveal
VM: Virtual machine
