# Supplementary material for: Recurrent tumor-specific regulation of alternative polyadenylation of cancer-related genes
Source: BMC Genomics. 2018 Jul 13;19:536. doi: 10.1186/s12864-018-4903-7 (PMC6045855; doi:10.1186/s12864-018-4903-7)
Supplement: Supplementary file 1 — Supplementary figures. It includes all supplementary figures except Figure S4. Figure S1. Benchmark of DaPars, KLEAT and ContextMap 2. Figure S2. Relevance of 114 select genes to cancer according to COSMIC. Figure S3. Detail of CS predictions. Figure S5. Distribution of 3’ UTR lengths of protein coding and NMD transcripts. (PDF 2210 kb) [file 12864_2018_4903_MOESM1_ESM.pdf]

# Supplementary Figures

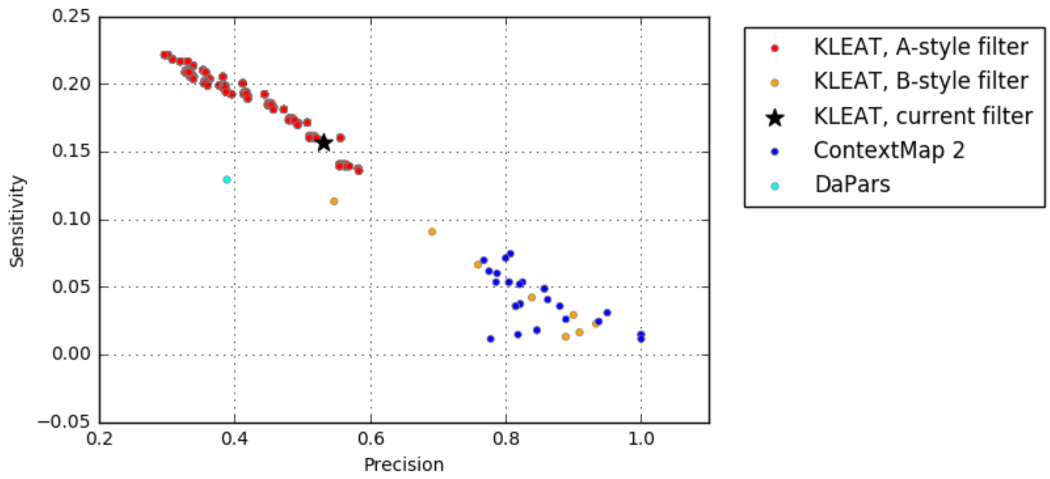


**Figure S1. Benchmark of DaPars, KLEAT and ContextMap 2**

To run prediction tools, we used the reads after the biobloomfilter step (Figure 1A). DaPars require two sets RNA-Seq data corresponding to different conditions, so we paired it with human brain reference (sample ID: mRNA-Brain-C4) together with university human reference RNA-Seq data. We extracted all the predicted CSs by DaPars regardless of dynamic APA. For KLEAT predictions, we used two styles of filtering. A-Style: consistent with the filter used in this study (Materials and Methods), the KLEAT predictions are filtered by all combinations of the following parameters: distance to the closest annotated CS ≤ [15: 95] bp (step size=10); hexamer is one of the two strongest hexamers [AATAAA, ATTAAA], or of all 16 hexamers used in KLEAT, [AATAAA,ATTAAA, AGTAAA, TATAAA, CATAAA, GATAAA, AATATA, AATACA, AATAGA, AAAAAG, ACTAAA, AAGAAA, AATGAA, TTTAAA, AAAACA, GGGGCT]; length_of_tail_in_contig ≥ [0: 6] (step size=2); number_of_bridge_reads ≥ [0: 6] (step size=2)]; max_bridge_read_tail_length ≥ [0: 6] (step size=2). B-style: consistent with the comparison conducted in the ContextMap 2's paper [1]; the KLEAT predictions are filtered by the number of tail+bridge reads ≥ [1,10] (step size=1). For ContextMap 2 predictions, we varied the two running parameters that have the biggest influence on the sensitivity: length of the sliding window: $w_l \in$ [4: 10] (step size=1), Minimum number of poly(A) reads supporting a set of pairwise overlapping poly(A) sites: $r_s \in$ [2: 5] (step size=1) [1]. The result corresponding to the filter used in this study is highlighted with a star. The benchmark is limited to 114 genes; we filtered both predictions results based on RNA-Seq data and PolyA-Seq data to only retain CSs of these genes. If the prediction and the closest cleavage site from PolyA-Seq data are within 50 bp, it is considered a true positive.

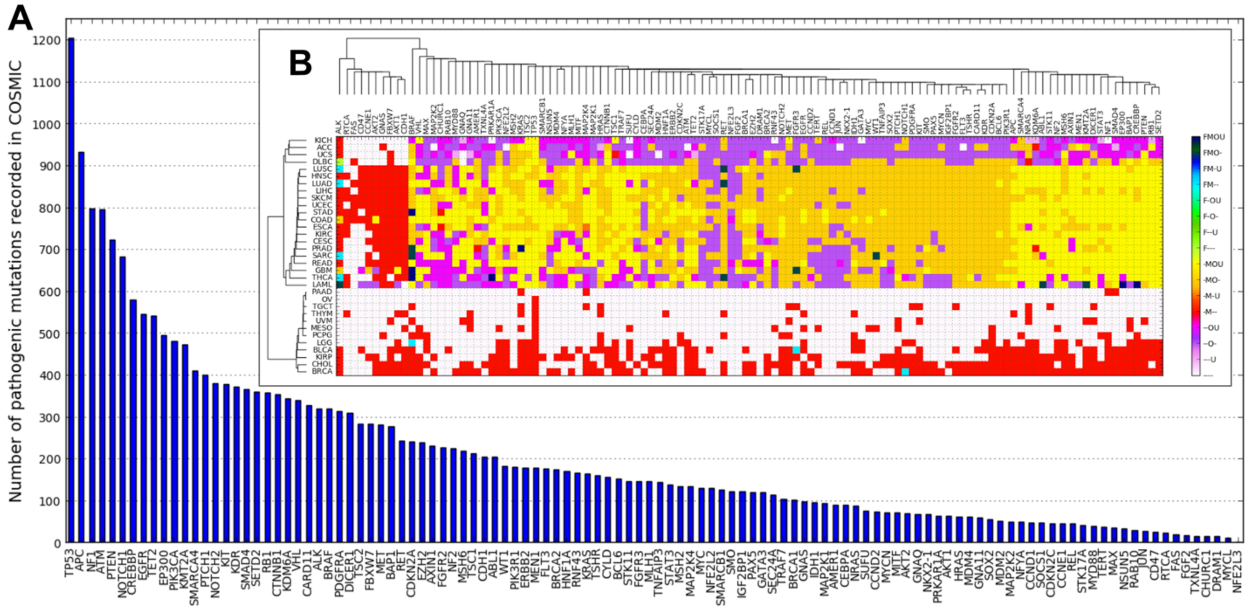


**Figure S2. Relevance of 114 curated genes to cancer according to COSMIC.**

(A) Number of pathogenic (FATHMM score ≥ 0.7) mutations per gene. All 114 genes have at least one pathogenic mutation. (B) Grid view of existence of fusion (F), mutation (M), over- (O), and under-expression (U) of genes among different cancer types. Each cell corresponds to a 4-dimension binary vector with 1 indicating an existence of F/M/O/U event. Genes and diseases were hierarchically clustered with single-linkage and hamming distance. All mutational data are obtained from the COSMIC v80 [2] database.

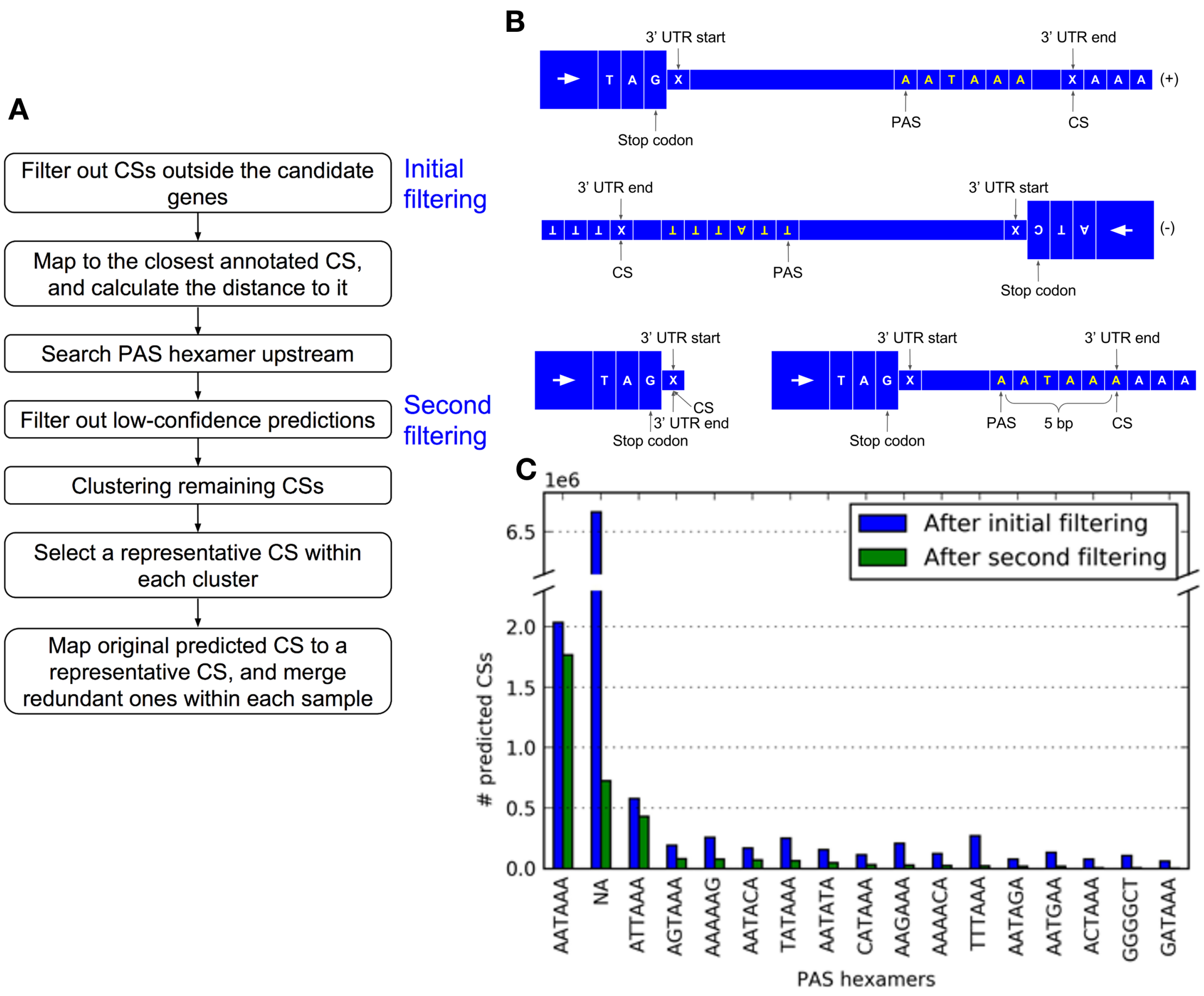


**Figure S3. More details of CS predictions.**

(A) Schematic diagram of the CS post-processing workflow after KLEAT prediction. The initial filtering removes predicted CSs that were not associated with any candidate genes. The second filtering selects high confidence predictions based on their distances to annotated CSs and polyadenylation evidence. (B) 1-based coordinate system for different 3’ UTR examples: 1) a plus strand 3’ UTR, 2) a minus strand 3’ UTR, 3). a 0-length 3’ UTR, 4) a 3’ UTR with the PAS hexamer motif located right upstream of the CS, so the minimum distance between the CS and the PAS is 5 bp. (**C**) Distribution of PAS hexamers within 50 bp upstream of predicted CSs. The motifs AATAAA and ATTAAA are the most prevalent PAS hexamers, both before and after the second filtering steps. The second filtering step removed most of the CSs without any PAS hexamer (NA) detected within 50 bp.

**NOTE**: The file is too large to be inserted here. Please see this figure in a separate file.

**Figure S4. Illustration of all 77 identified events of tumor-specific cleavage patterns.**

Legends in Figure 2 and Figure 3 apply. However, different from Figure 2 and Figure 3 in the main text, all predicted CSs are plotted without filtering by the low frequency ($<5\%$) and insignificant change criteria. As a result, the number of CSs for the same gene in different cancer types may differ. The *FGF2*-THCA event is included as a negative control. In addition, we also computed the distribution of expression in reads per kilobase of transcript per million sequenced reads (RPKMS) [3].

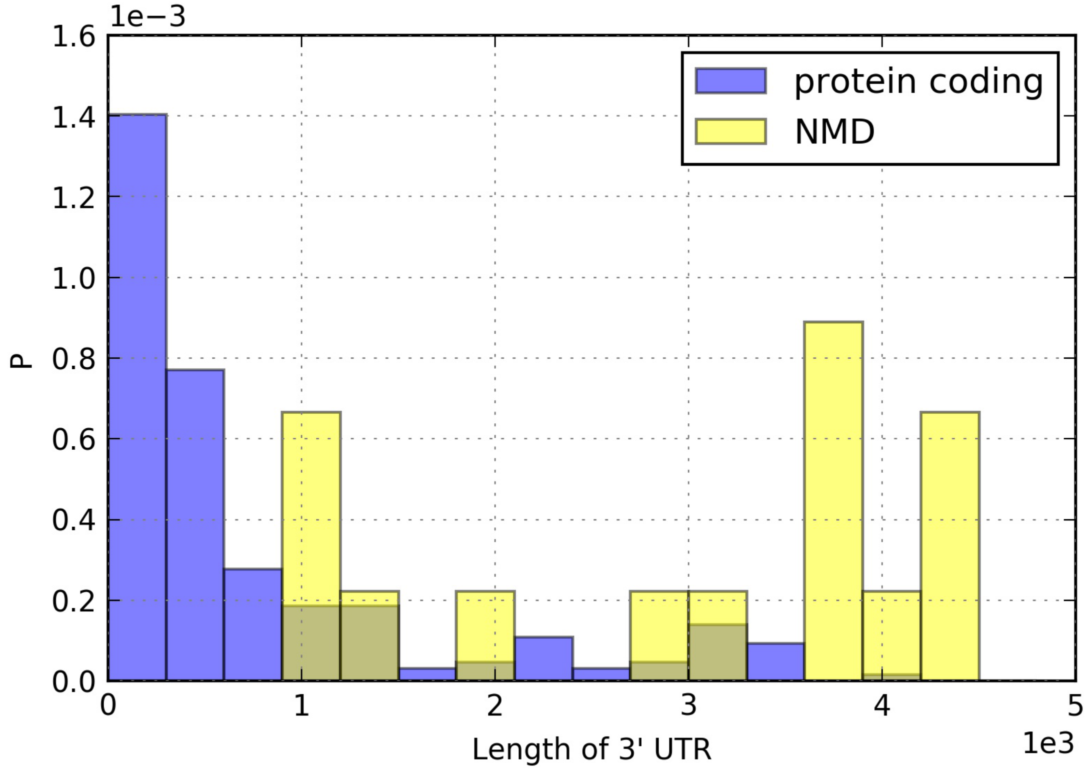


**Figure S5. Distribution of 3’ UTR lengths of protein coding and NMD transcripts.**

The mean length of protein coding 3’ UTRs is 2,158 bp, while that of NMD transcripts is 12,721 bp. The difference is significant ($P = 6.5x10^{-16}$, KS-test). The distribution is calculated based on predicted CSs that are mapped to only one stop codon. If introns exist in the 3’ UTRs under comparison, they are also included. Y axis means relative frequency.

# References

1. Bonfert T, Friedel CC. Prediction of Poly(A) Sites by Poly(A) Read Mapping. Tian B, editor. PLoS One. 2017;12:e0170914.

2. Forbes SA, Beare D, Gunasekaran P, Leung K, Bindal N, Boutselakis H, et al. COSMIC: exploring the world's knowledge of somatic mutations in human cancer. Nucleic Acids Res. 2015;43:D805–11.

3. Gibb EA, Warren RL, Wilson GW, Brown SD, Robertson GA, Morin GB, et al. Activation of an endogenous retrovirus-associated long non-coding RNA in human adenocarcinoma. Genome Med. 2015;7:22.
