# Supplementary material for: Recurrent tumor-specific regulation of alternative polyadenylation of cancer-related genes
Source: BMC Genomics. 2018 Jul 13;19:536. doi: 10.1186/s12864-018-4903-7 (PMC6045855; doi:10.1186/s12864-018-4903-7)
Supplement: Supplementary file 3 — Figure S4. Illustration of all 77 identified events of tumor-specific cleavage patterns. (PDF 5699 kb) [file 12864_2018_4903_MOESM3_ESM.pdf]

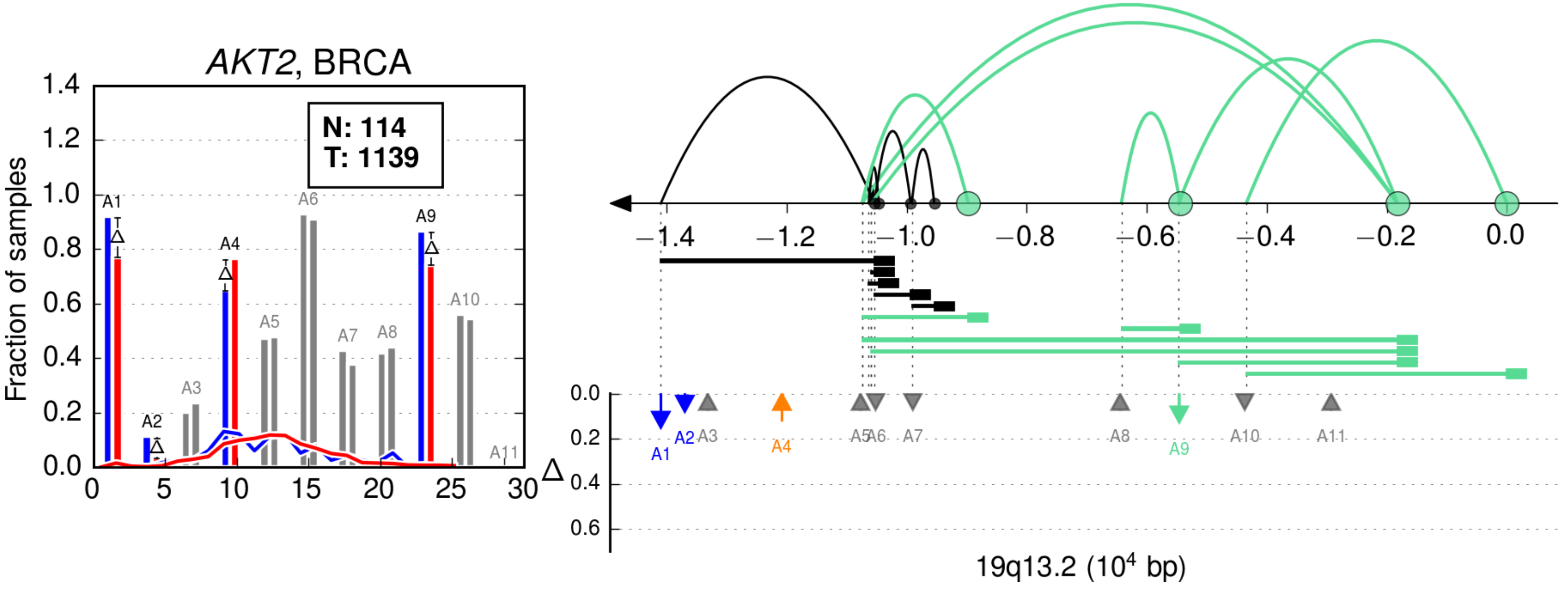

AKT2, BRCA
N: 114
T: 1139
Fraction of samples
A1
A2
A3
A4
A5
A6
A7
A8
A9
A10
A11
Δ
19q13.2 ($10^4$ bp)

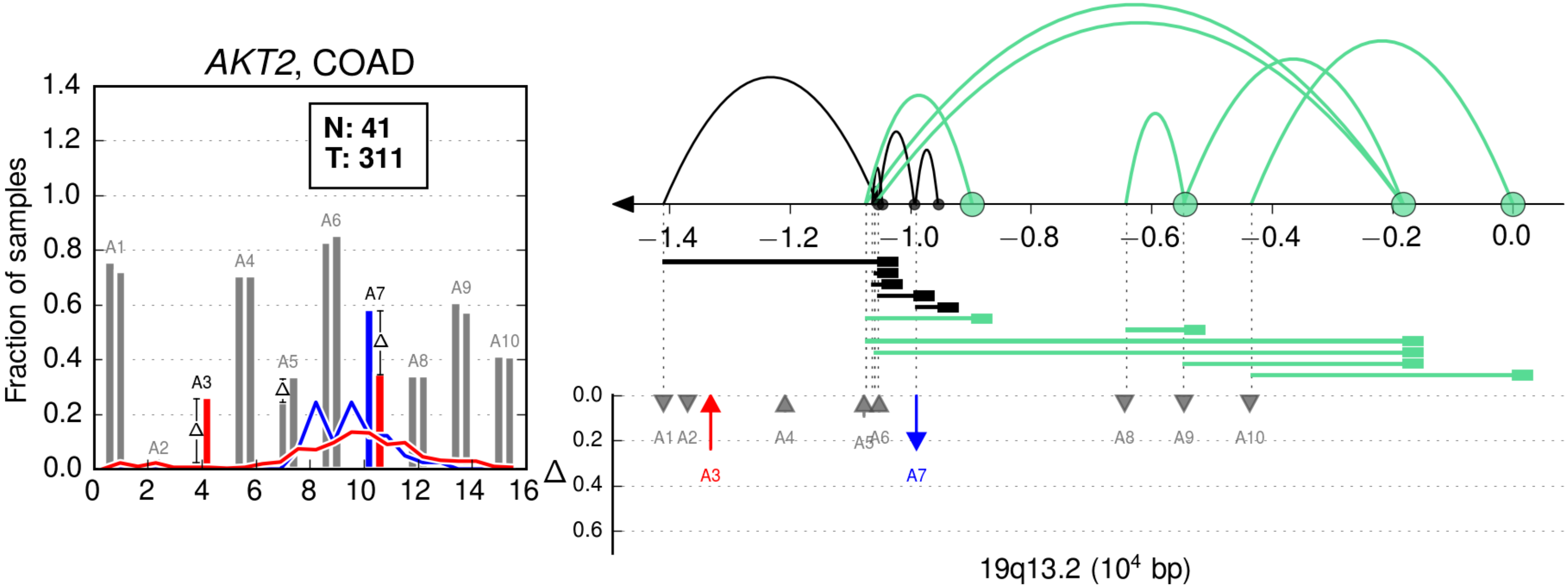

AKT2, COAD
N: 41
T: 311
Fraction of samples
A1
A2
A3
A4
A5
A6
A7
A8
A9
A10
Δ
19q13.2 (10^4 bp)

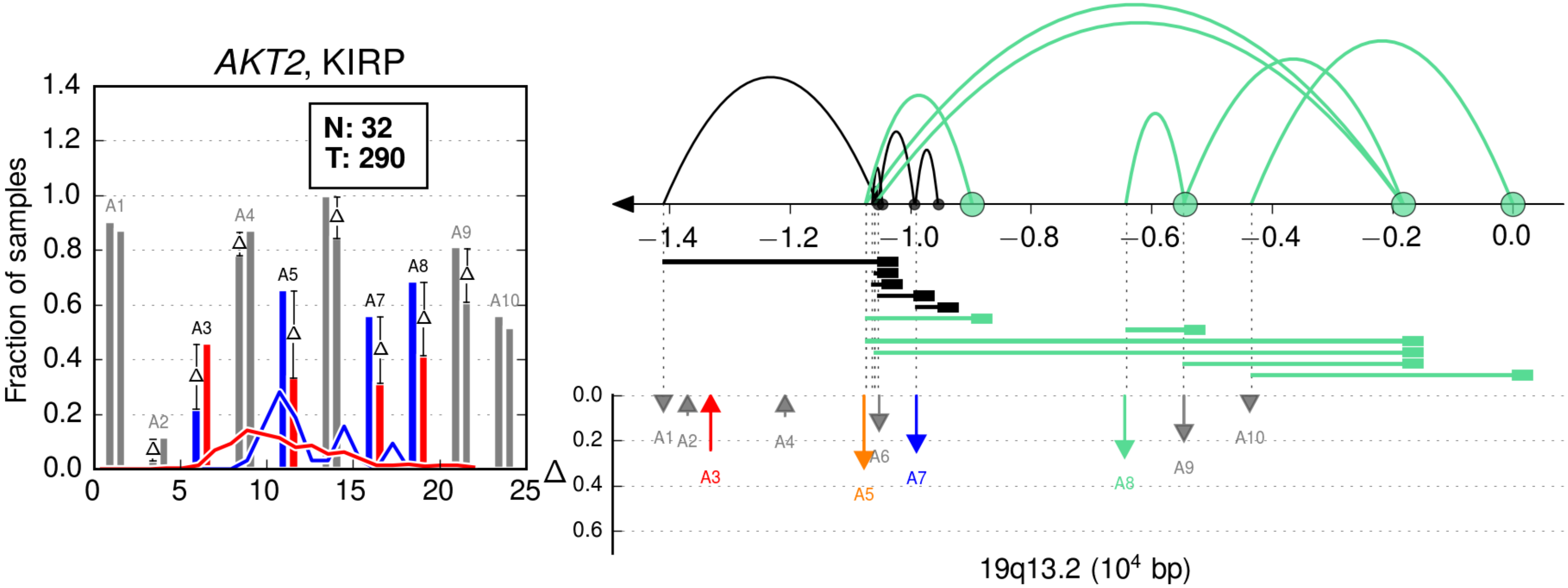
AKT2, KIRP
N: 32
T: 290
Fraction of samples
A1
A2
A3
A4
A5
A7
A8
A9
A10
Δ
19q13.2 ($10^4$ bp)
A1 A2 A3 A4 A5 A6 A7 A8 A9 A10

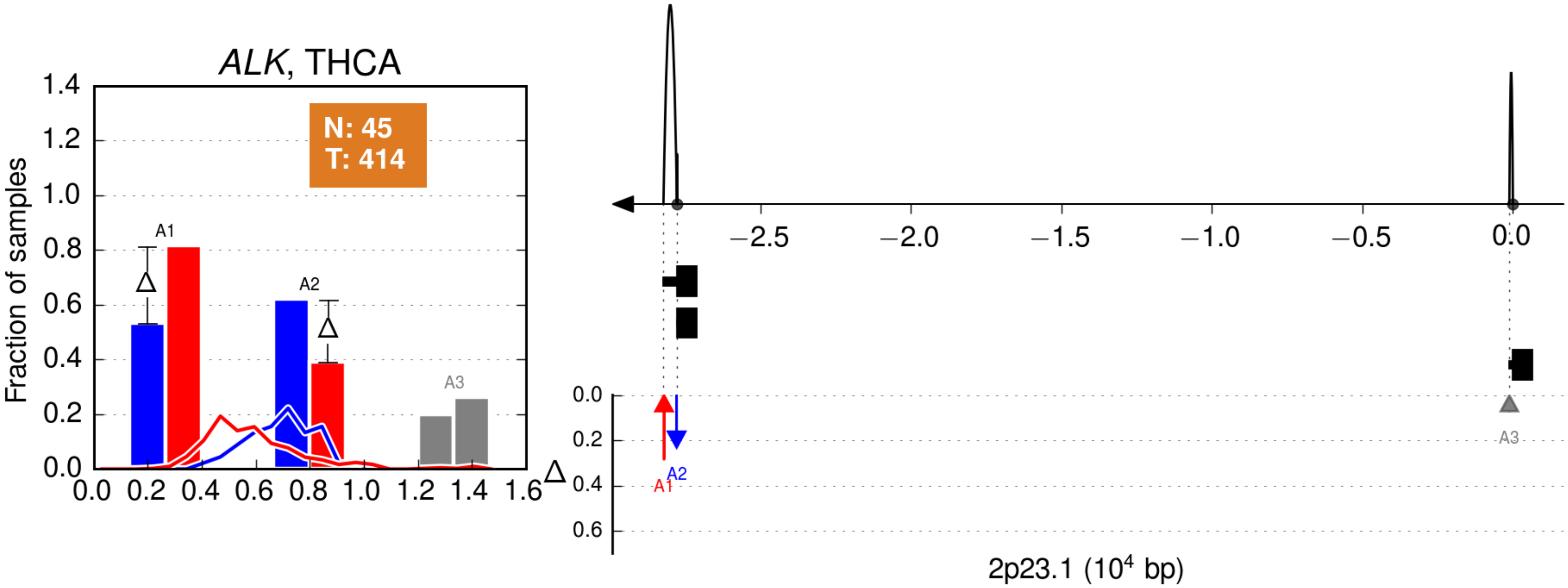

ALK, THCA
N: 45
T: 414
Fraction of samples
A1
A2
A3
Δ
2p23.1 (10^4 bp)

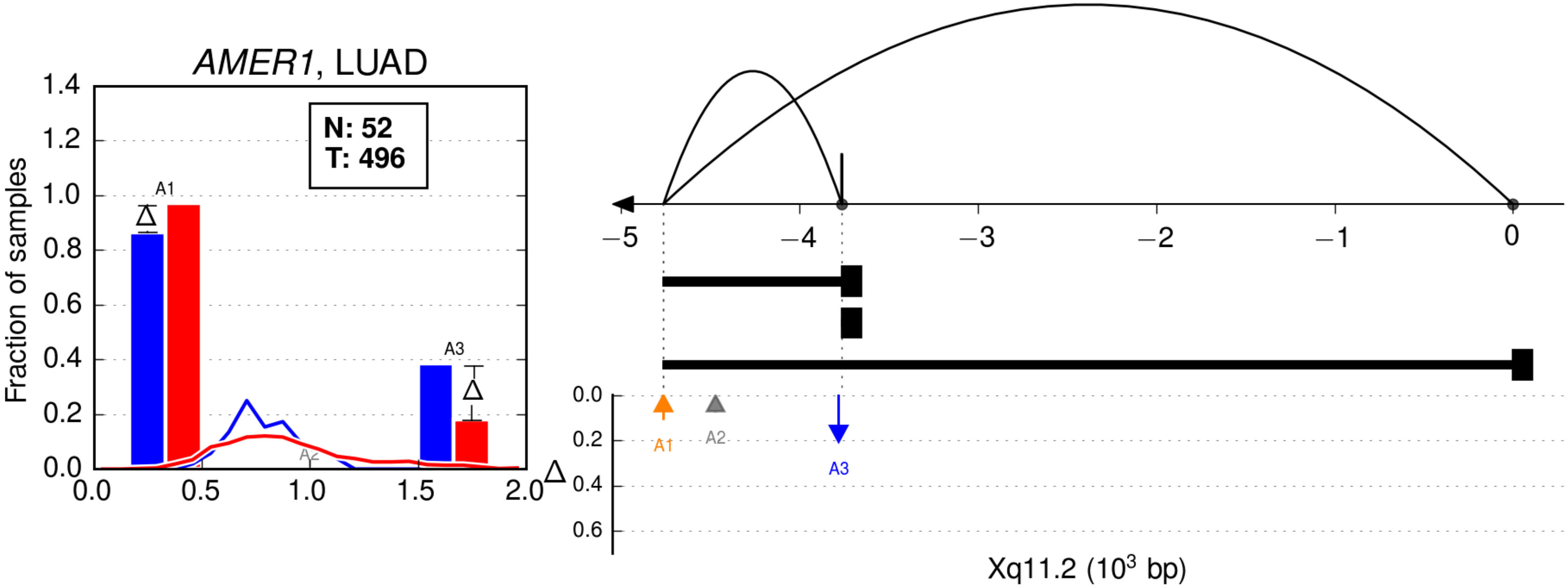

AMER1, LUAD
N: 52
T: 496
Fraction of samples
A1
A2
A3
Δ
Xq11.2 ($10^3$ bp)

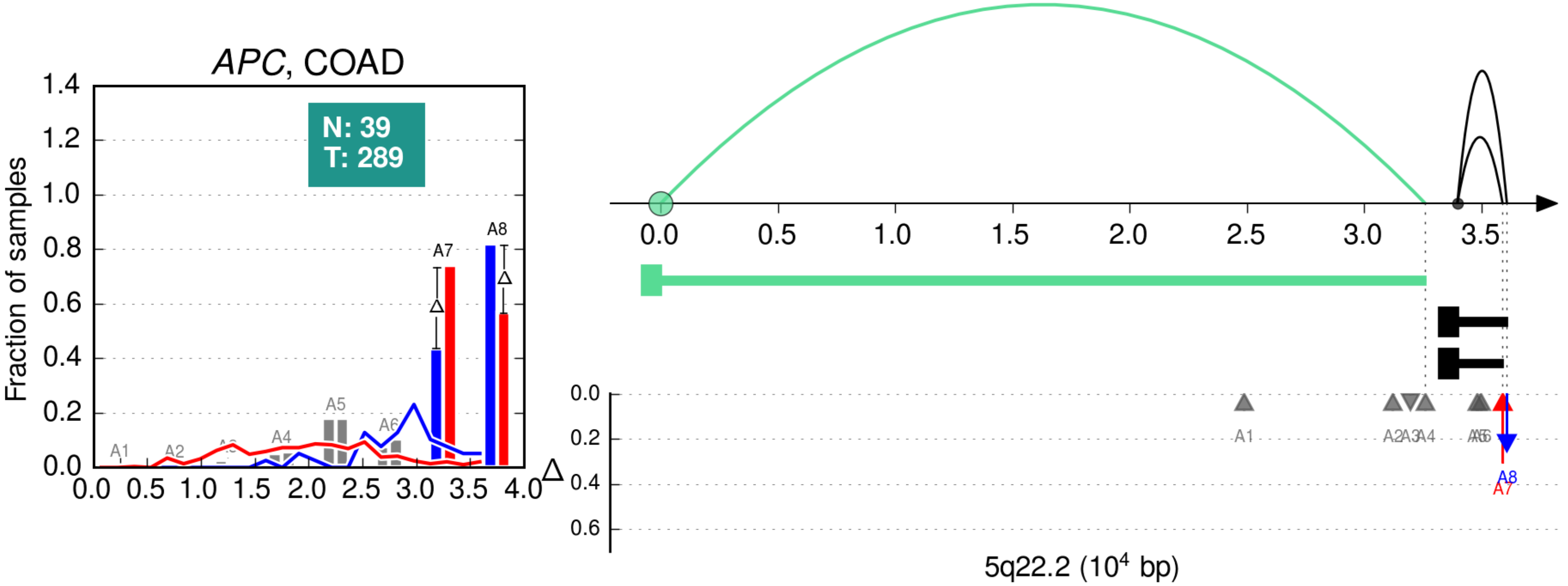

APC, COAD
N: 39
T: 289
Fraction of samples
A1
A2
A4
A5
A6
A7
A8
Δ
5q22.2 ($10^4$ bp)

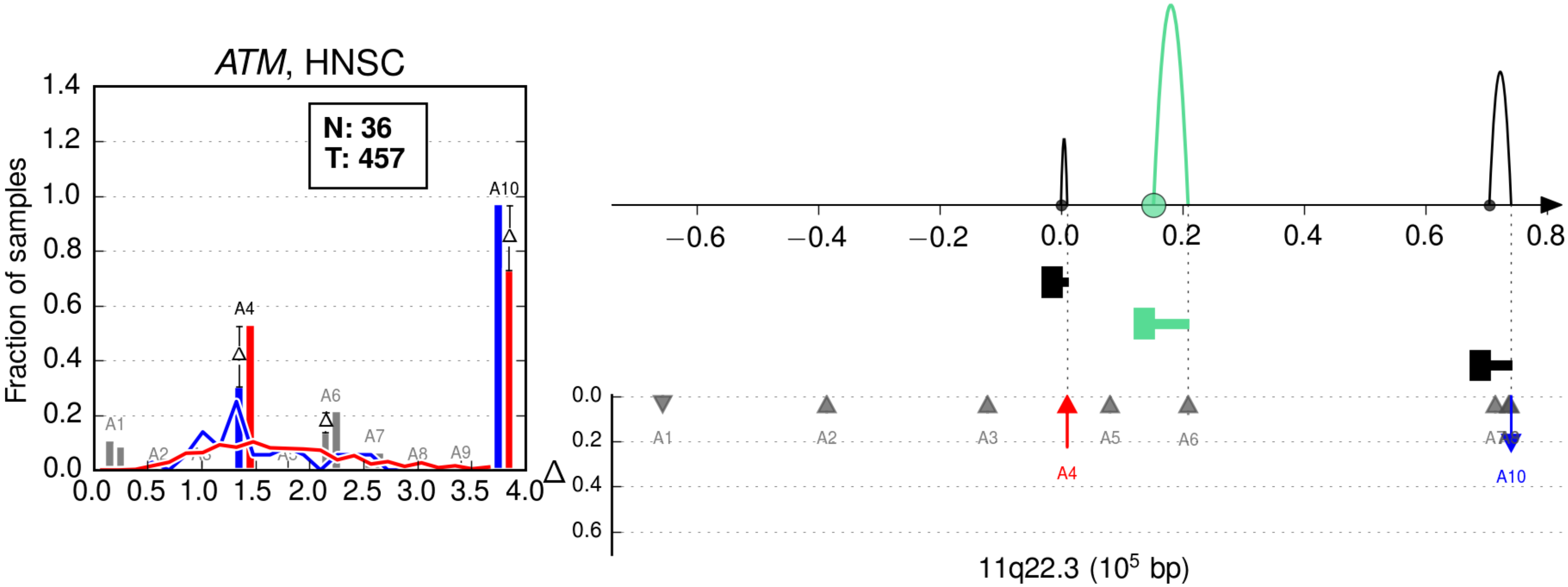

ATM, HNSC
N: 36
T: 457
Fraction of samples
1.4
1.2
1.0
0.8
0.6
0.4
0.2
0.0
0.0 0.5 1.0 1.5 2.0 2.5 3.0 3.5 4.0
Δ
A1 A2 A3 A4 A5 A6 A7 A8 A9 A10
−0.6 −0.4 −0.2 0.0 0.2 0.4 0.6 0.8
0.0
0.2
0.4
0.6
A1 A2 A3 A4 A5 A6 A7 A10
11q22.3 ($10^5$ bp)

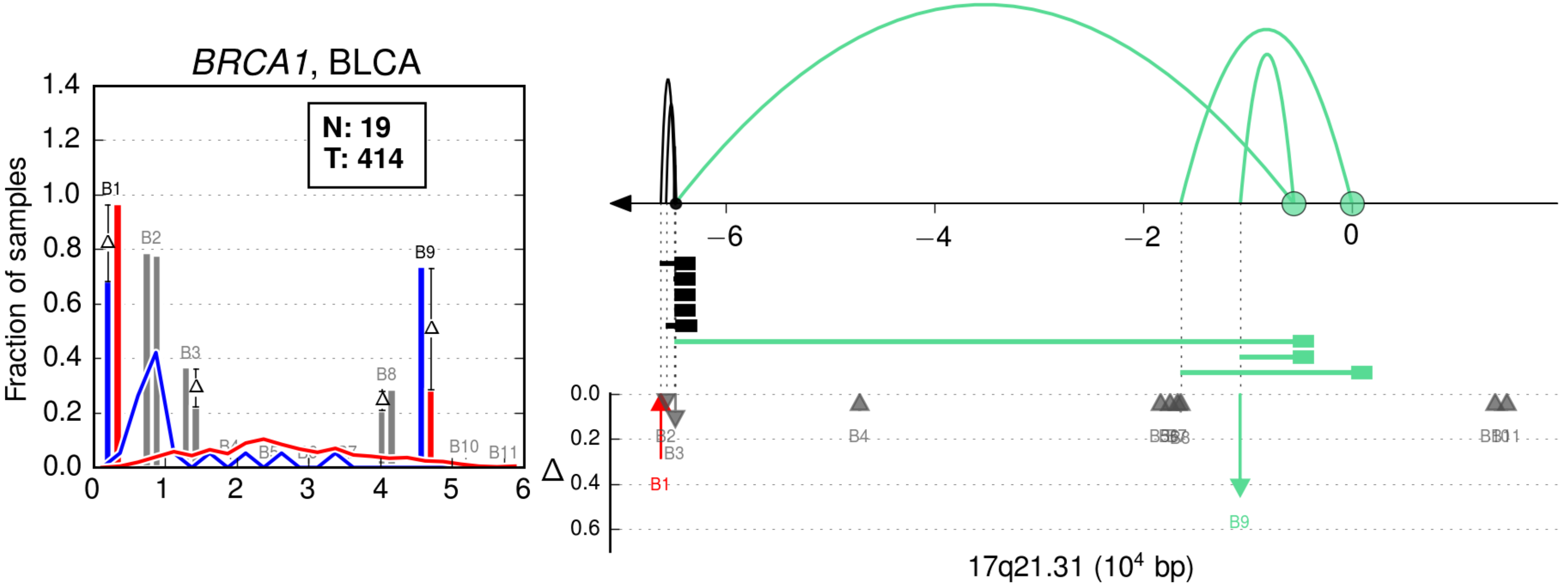

*BRCA1*, BLCA
N: 19
T: 414
Fraction of samples
B1
B2
B3
B8
B9
B10
B11
Δ
17q21.31 ($10^4$ bp)

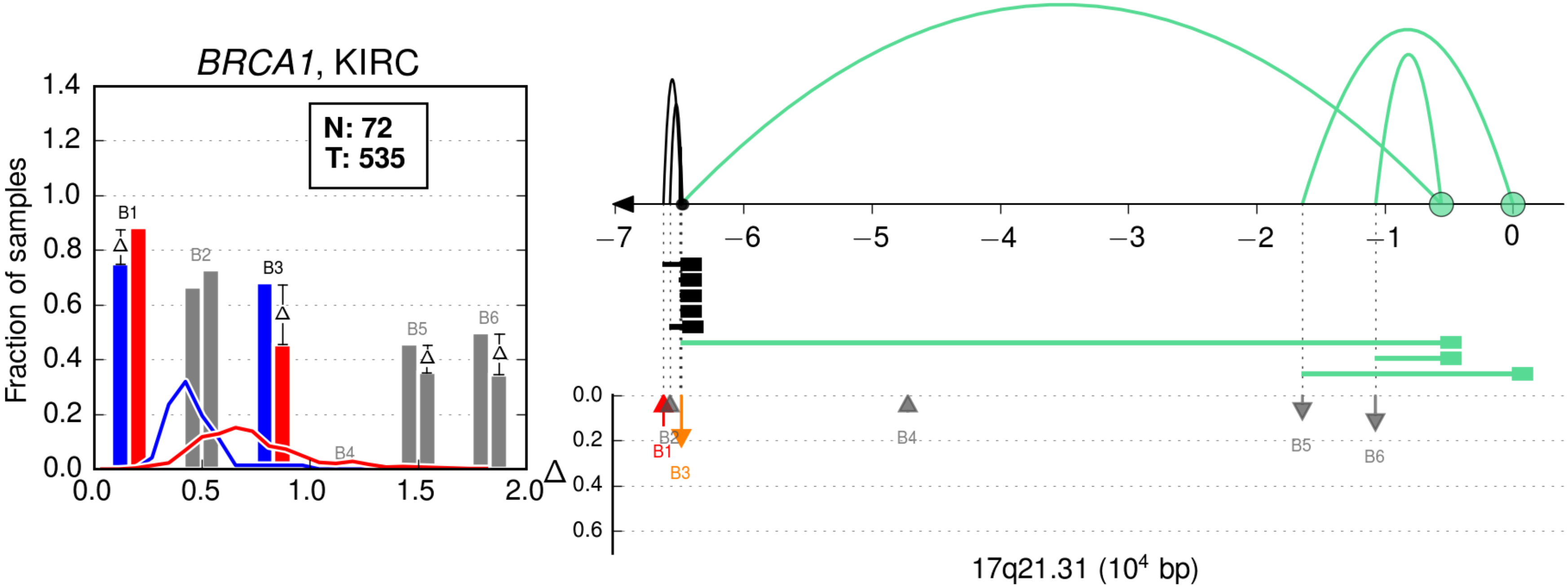

BRCA1, KIRC
N: 72
T: 535
Fraction of samples
B1
B2
B3
B4
B5
B6
Δ
17q21.31 ($10^4$ bp)

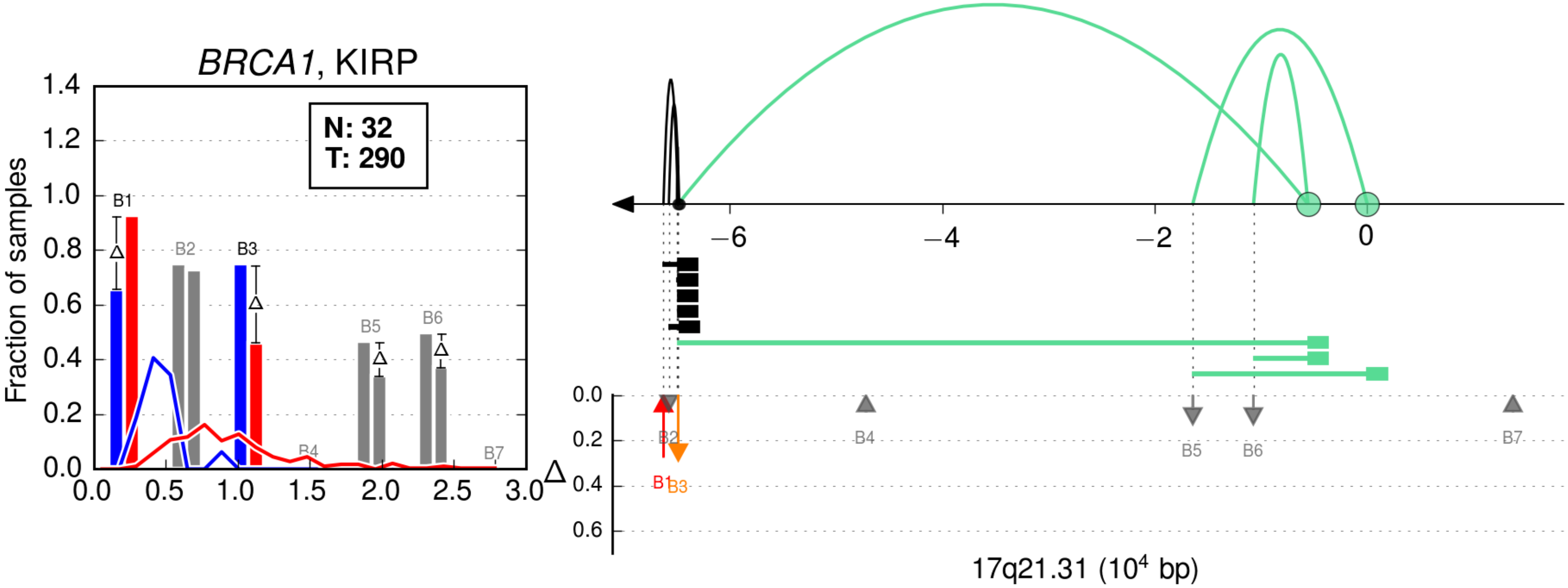

BRCA1, KIRP
N: 32
T: 290
Fraction of samples
B1
B2
B3
B4
B5
B6
B7
Δ
17q21.31 (10^4 bp)

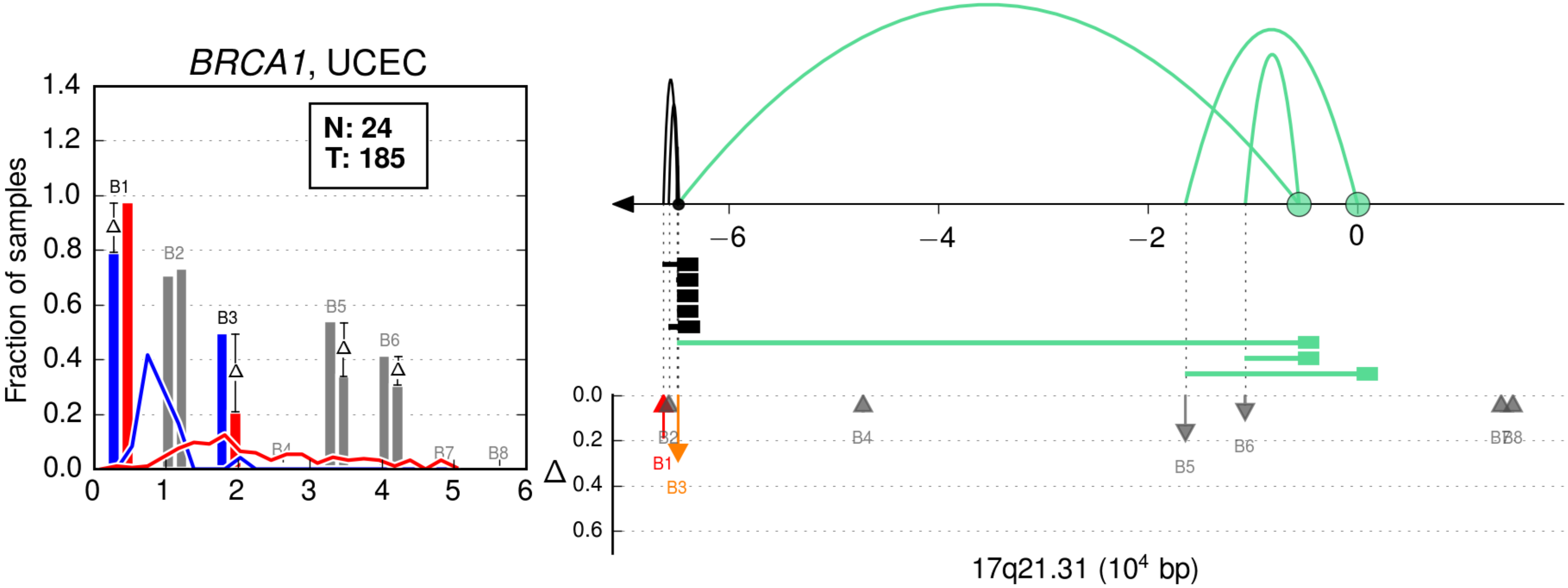
BRCA1, UCEC
N: 24
T: 185
Fraction of samples
B1
B2
B3
B4
B5
B6
B7
B8
Δ
17q21.31 ($10^4$ bp)

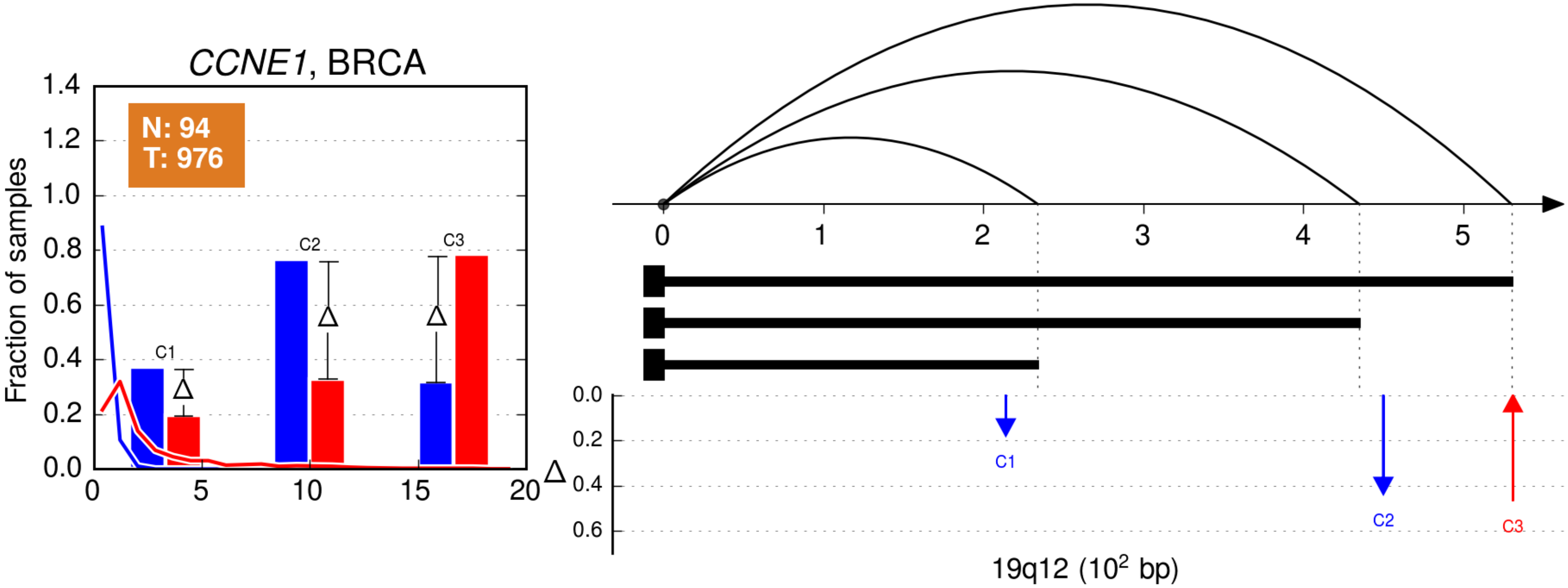

CCNE1, BRCA
N: 94
T: 976
Fraction of samples
1.4
1.2
1.0
0.8
0.6
0.4
0.2
0.0
0
5
10
15
20
C1
C2
C3
Δ
0
1
2
3
4
5
0.0
0.2
0.4
0.6
C1
C2
C3
19q12 (10² bp)

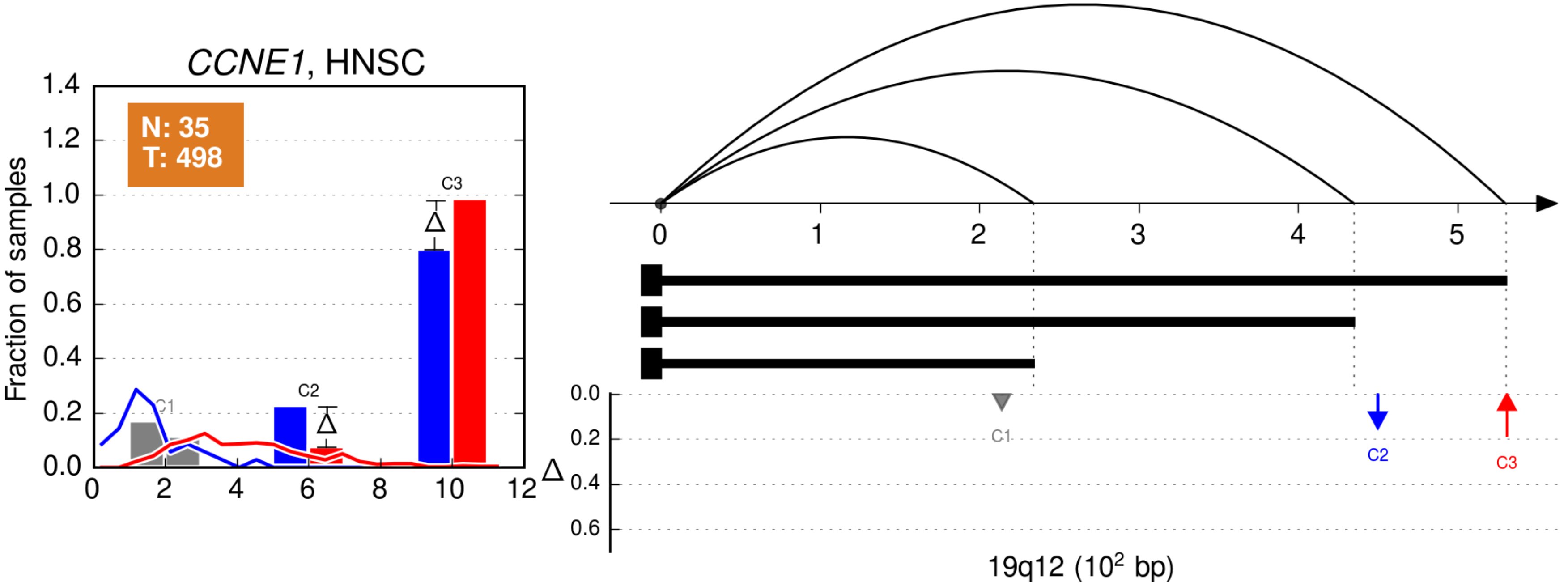

CCNE1, HNSC
N: 35
T: 498
Fraction of samples
1.4
1.2
1.0
0.8
0.6
0.4
0.2
0.0
0
2
4
6
8
10
12
C1
C2
C3
Δ
0
1
2
3
4
5
0.0
0.2
0.4
0.6
19q12 (10² bp)

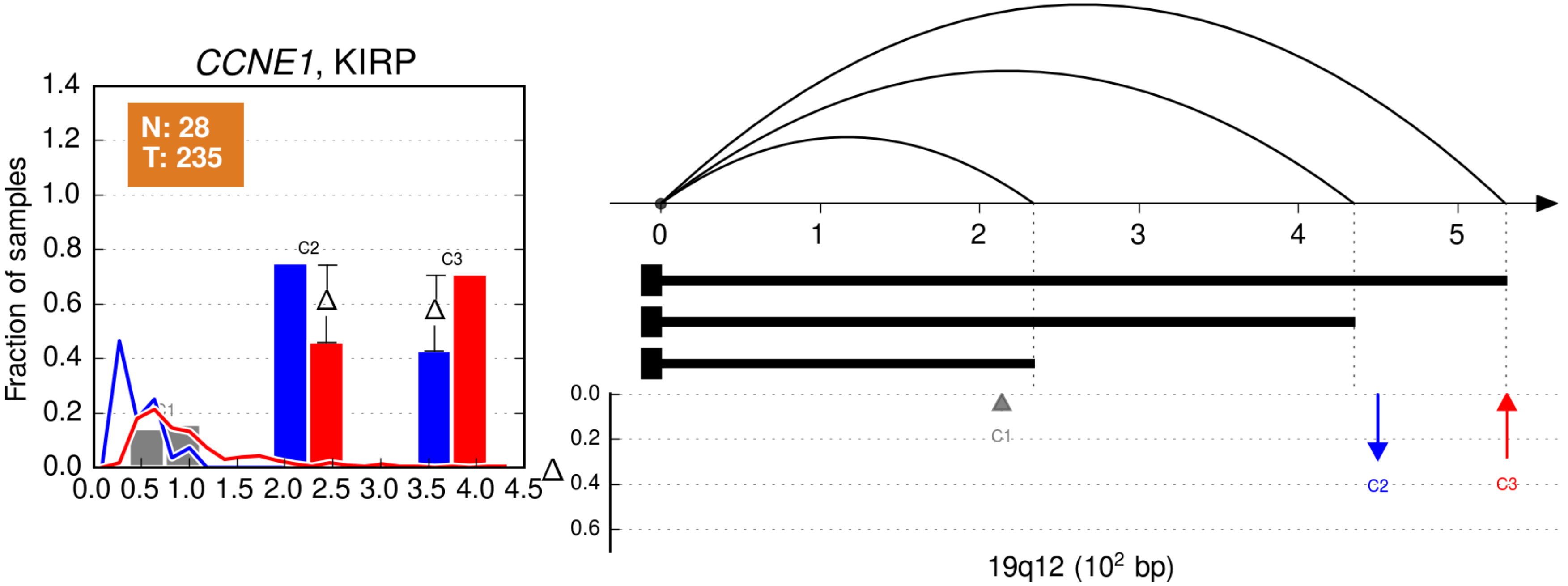

CCNE1, KIRP
N: 28
T: 235
Fraction of samples
1.4
1.2
1.0
0.8
0.6
0.4
0.2
0.0
C1
C2
C3
0.0 0.5 1.0 1.5 2.0 2.5 3.0 3.5 4.0 4.5
Δ
0 1 2 3 4 5
0.0
0.2
0.4
0.6
C1
C2
C3
19q12 (10^2 bp)

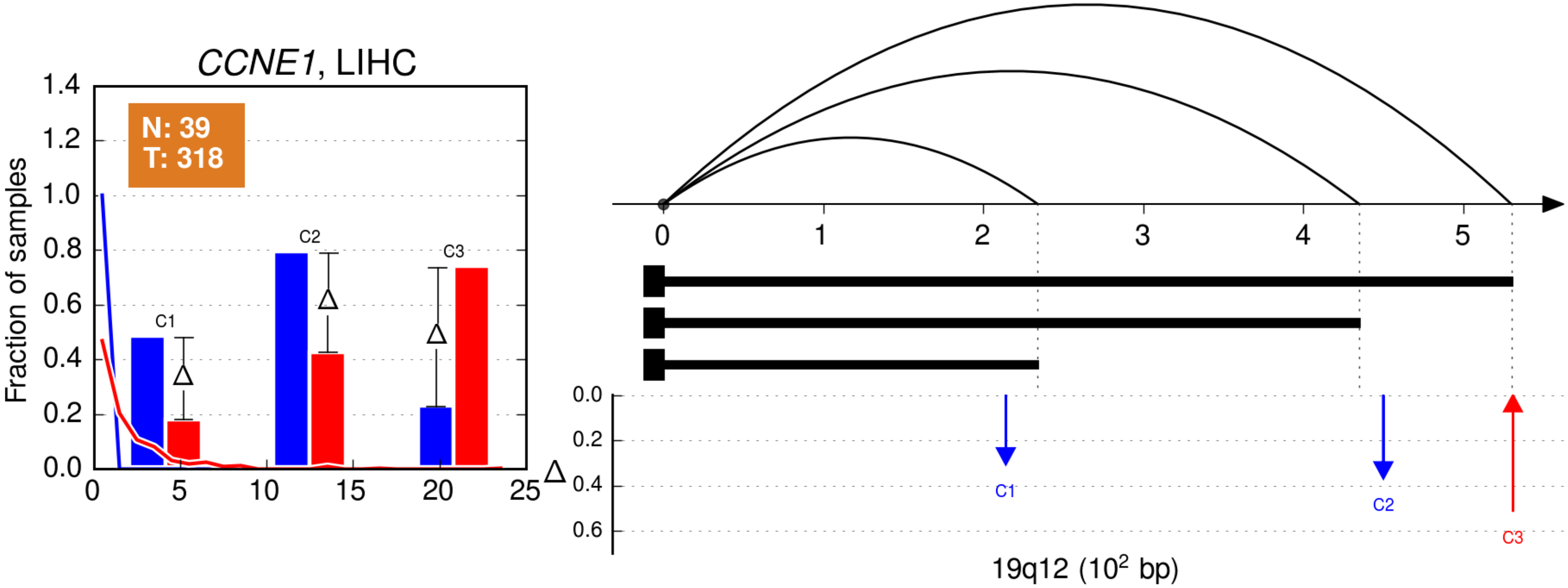
CCNE1, LIHC
N: 39
T: 318
Fraction of samples
C1
C2
C3
Δ
0
1
2
3
4
5
19q12 (10^2 bp)

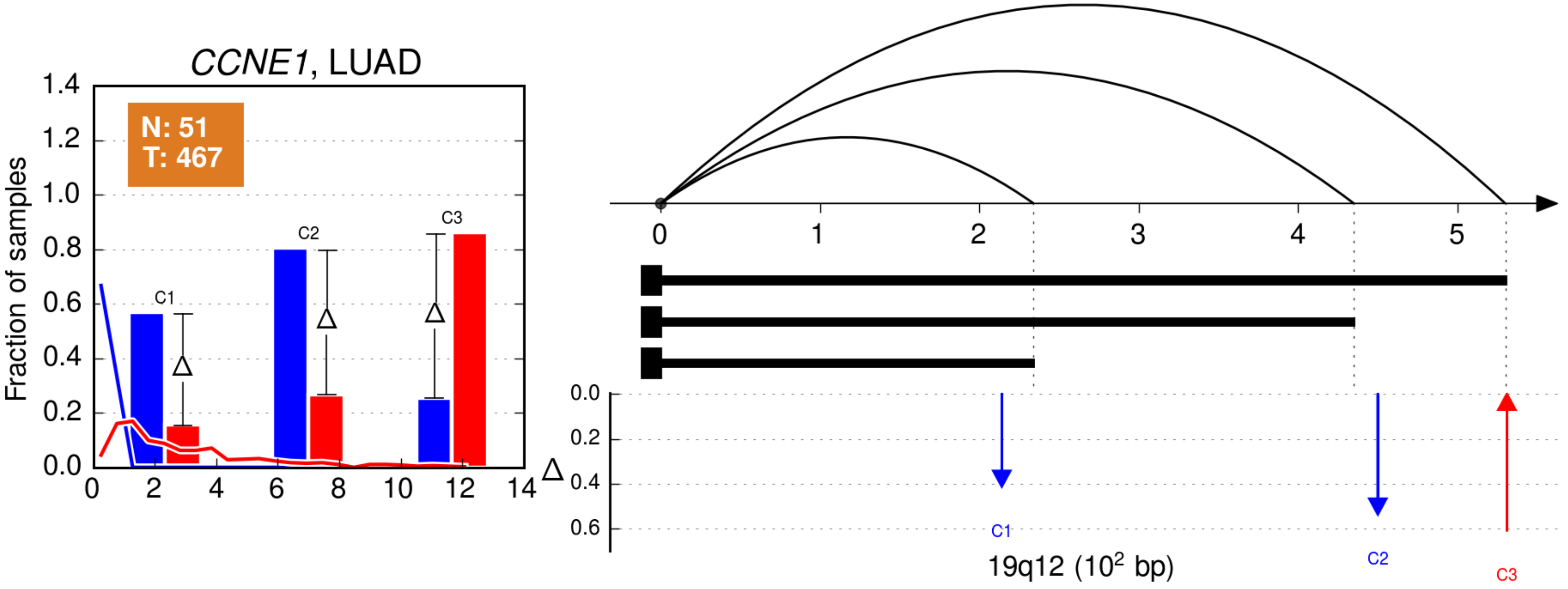

CCNE1, LUAD
N: 51
T: 467
Fraction of samples
1.4
1.2
1.0
0.8
0.6
0.4
0.2
0.0
0
2
4
6
8
10
12
14
C1
C2
C3
Δ
0
1
2
3
4
5
0.0
0.2
0.4
0.6
C1
C2
C3
19q12 (10² bp)

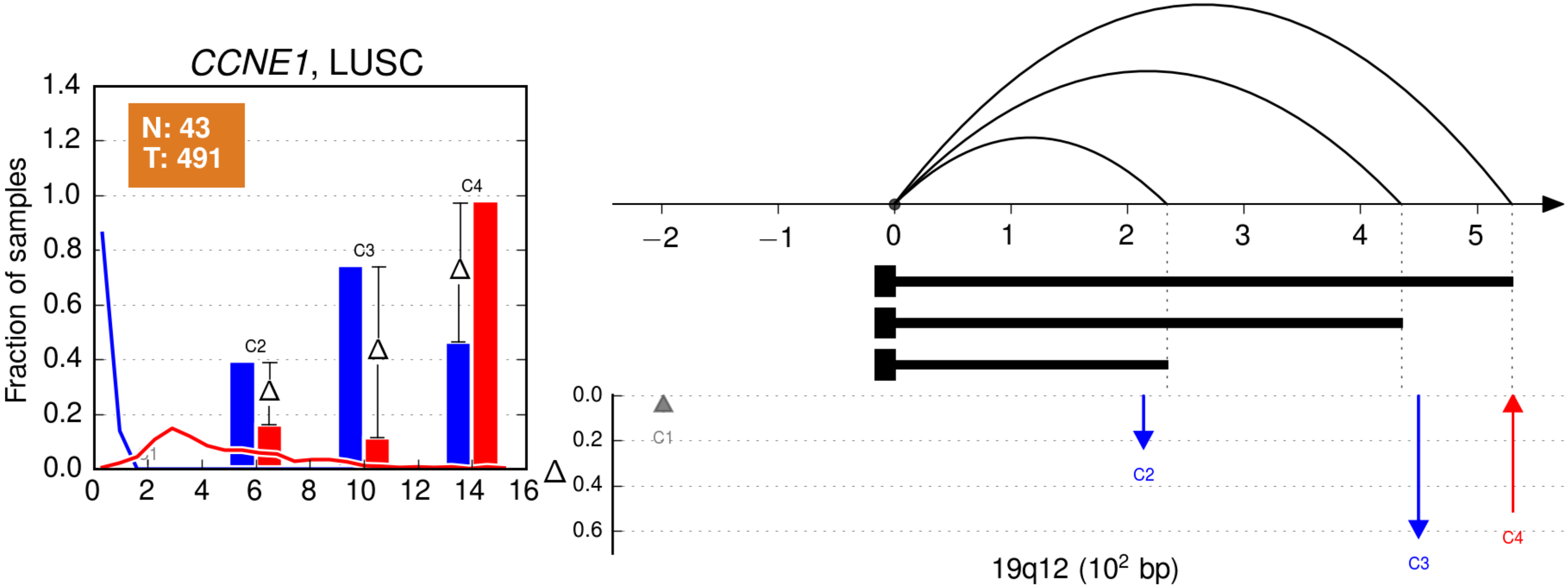

CCNE1, LUSC
N: 43
T: 491
Fraction of samples
C1
C2
C3
C4
Δ
19q12 ($10^2$ bp)

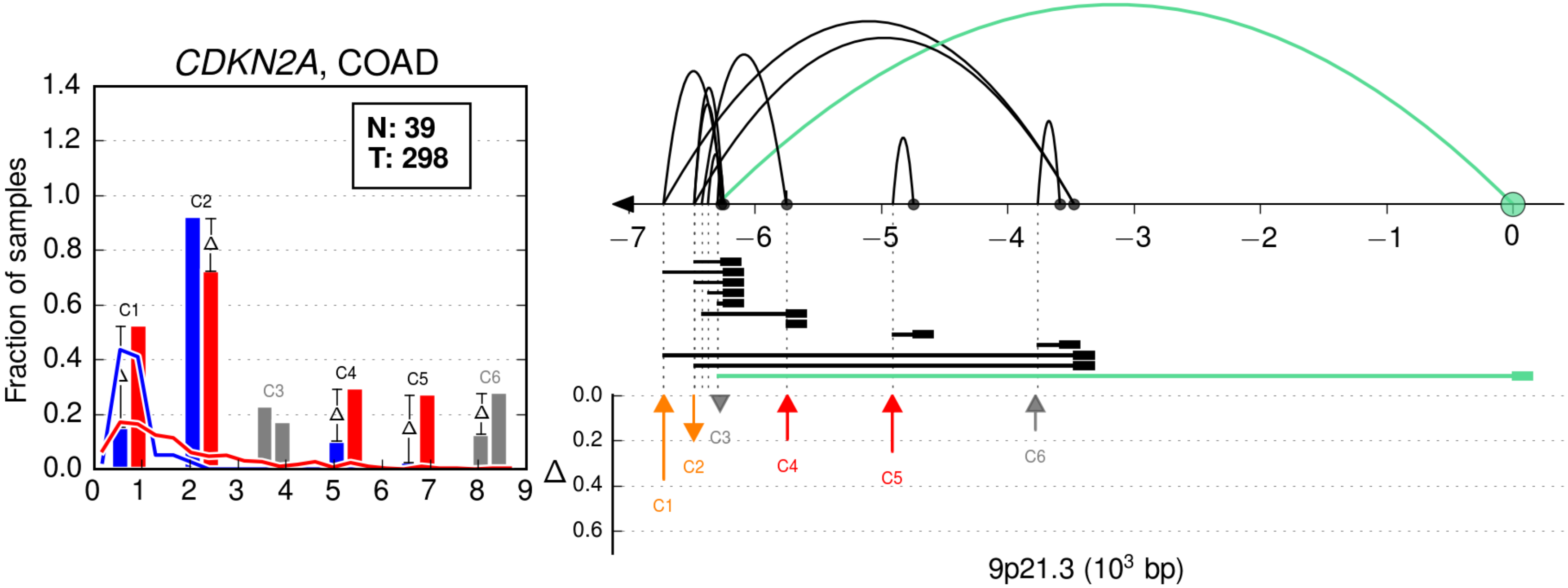

CDKN2A, COAD
N: 39
T: 298
Fraction of samples
C1
C2
C3
C4
C5
C6
Δ
9p21.3 (10^3 bp)

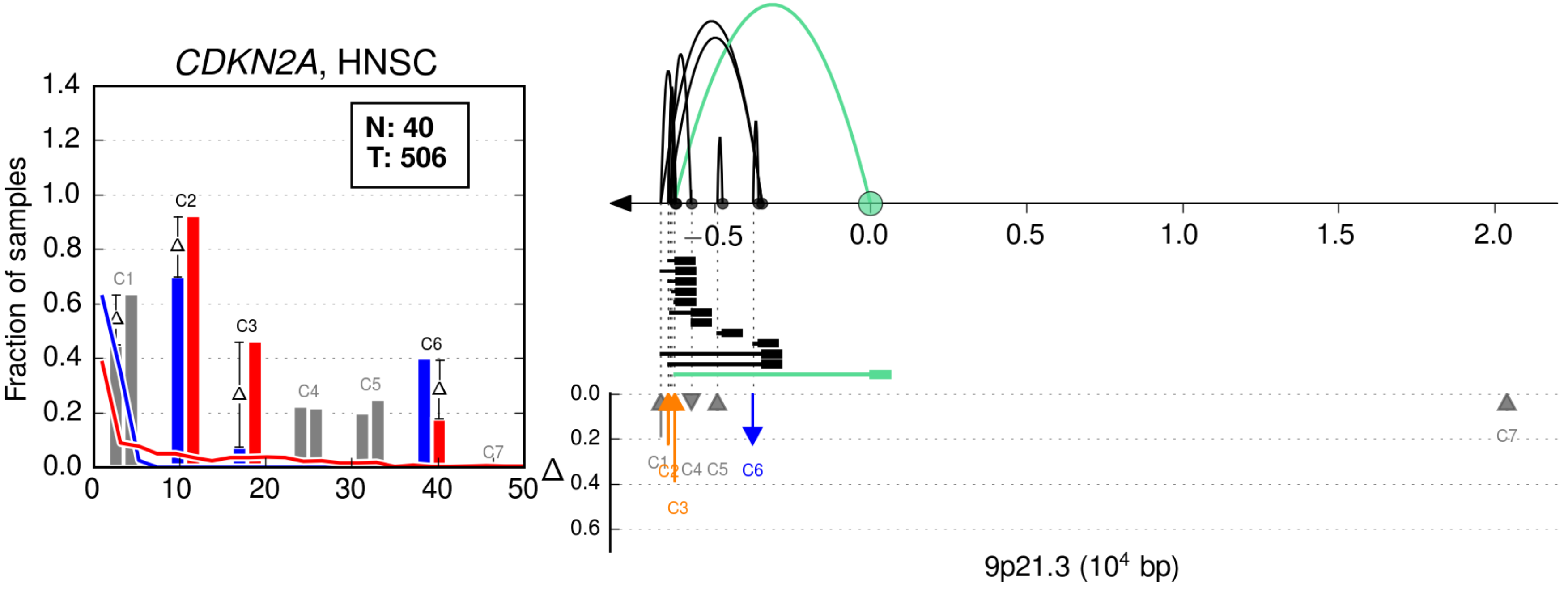

CDKN2A, HNSC
N: 40
T: 506
Fraction of samples
C1
C2
C3
C4
C5
C6
C7
Δ
9p21.3 (10^4 bp)

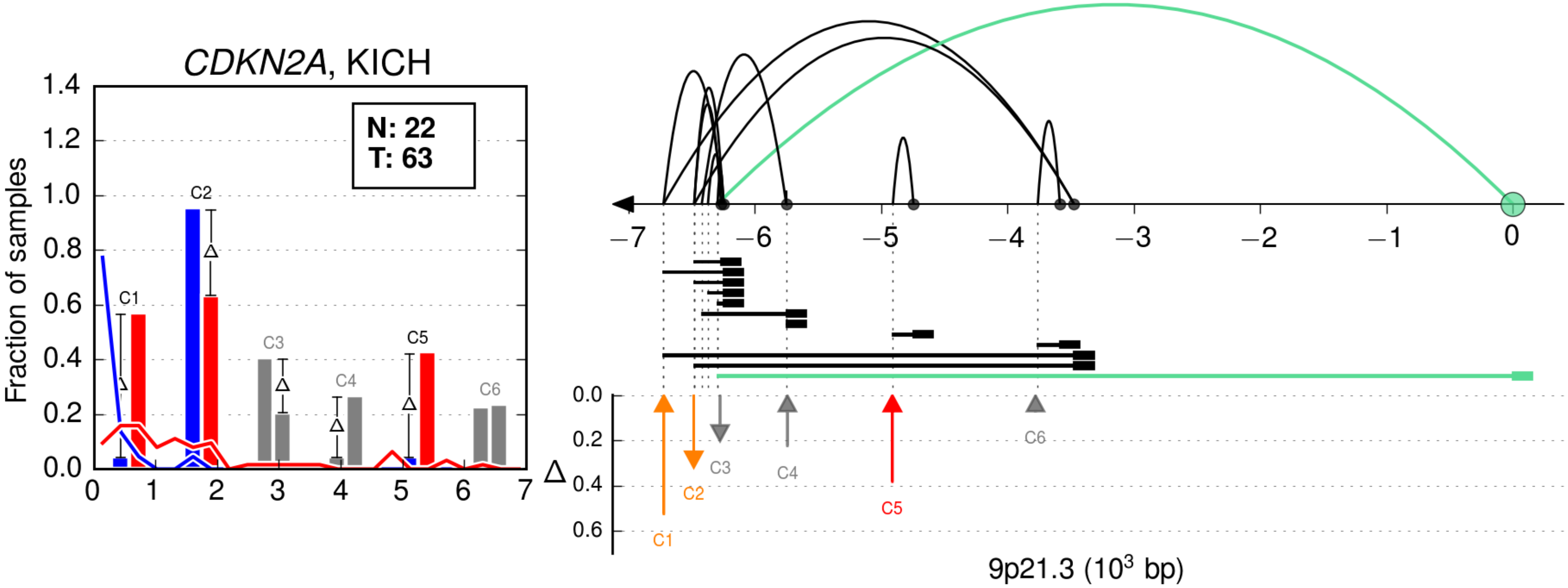

CDKN2A, KICH
N: 22
T: 63
Fraction of samples
C1
C2
C3
C4
C5
C6
Δ
9p21.3 (10³ bp)

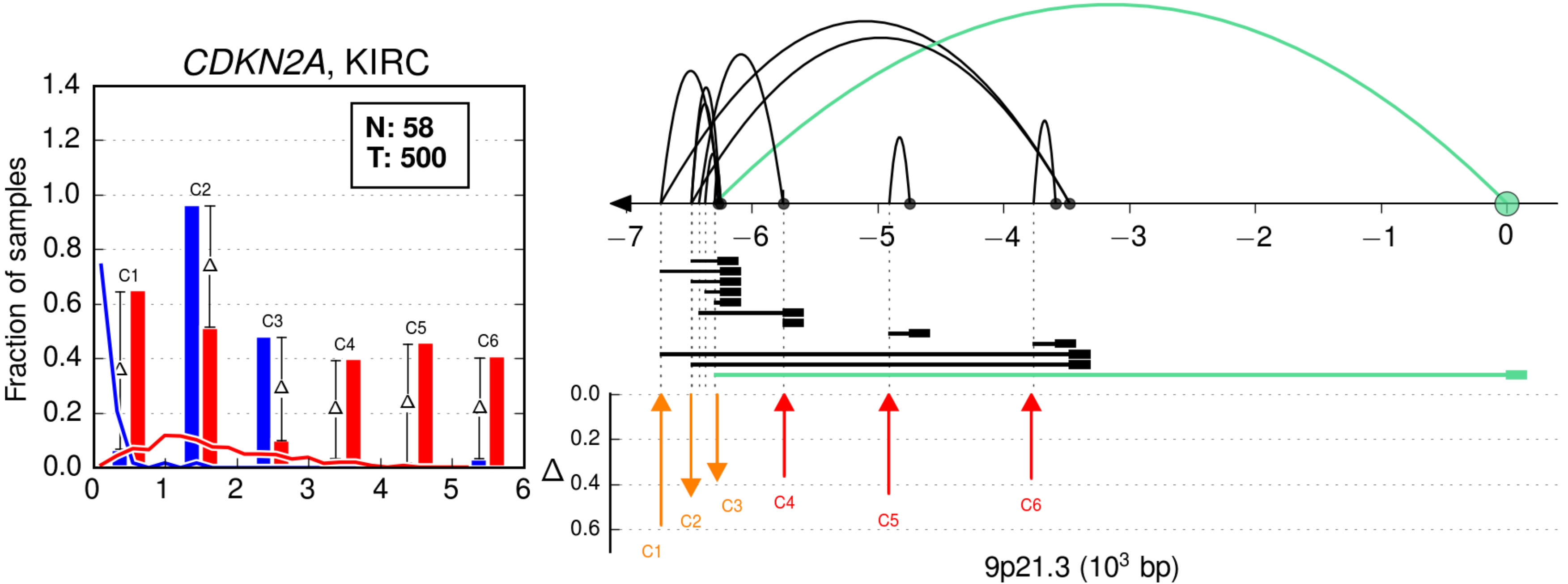

CDKN2A, KIRC
N: 58
T: 500
Fraction of samples
C1
C2
C3
C4
C5
C6
Δ
9p21.3 ($10^3$ bp)

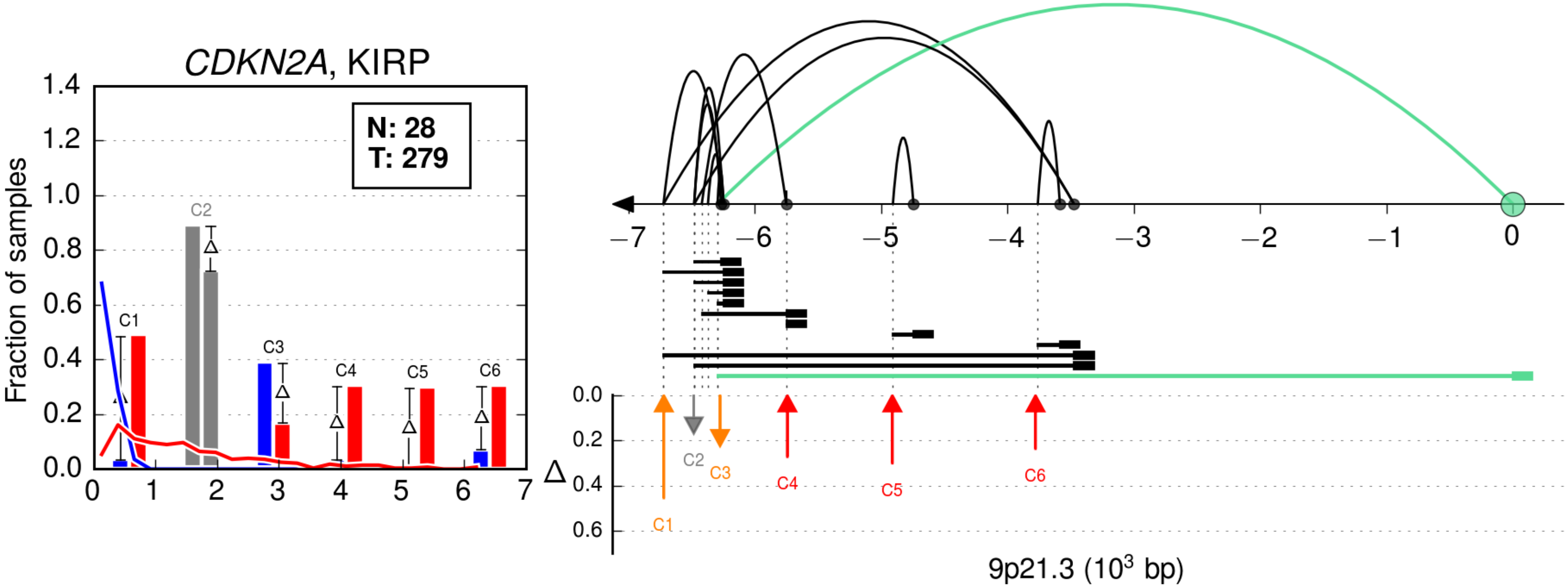
CDKN2A, KIRP
N: 28
T: 279
Fraction of samples
C1
C2
C3
C4
C5
C6
Δ
9p21.3 (10^3 bp)

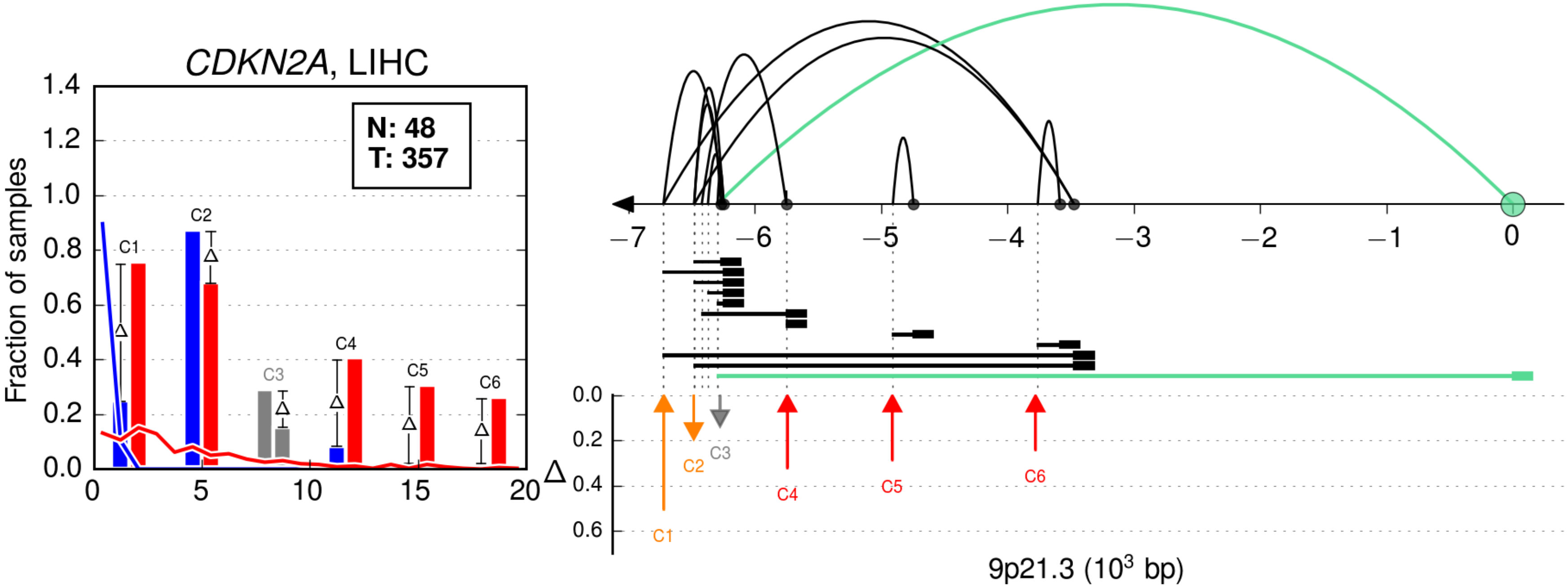

CDKN2A, LIHC
N: 48
T: 357
Fraction of samples
C1
C2
C3
C4
C5
C6
Δ
9p21.3 ($10^3$ bp)

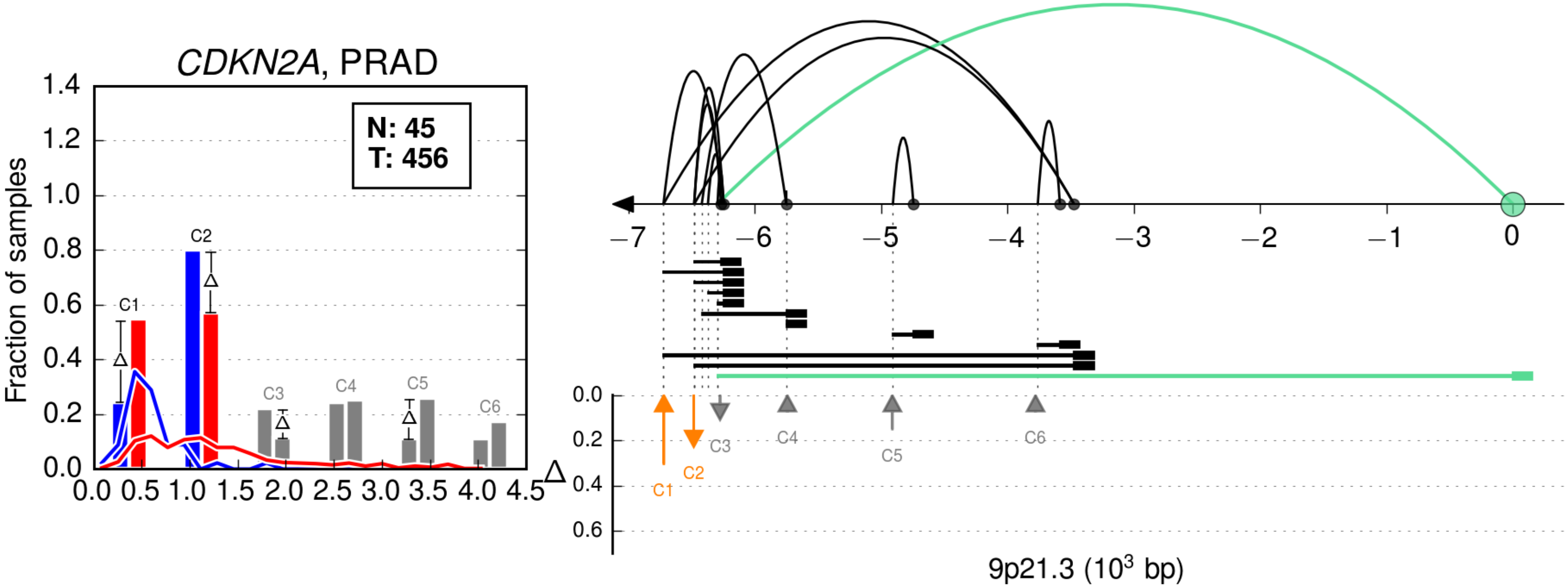

CDKN2A, PRAD
N: 45
T: 456
Fraction of samples
C1
C2
C3
C4
C5
C6
Δ
9p21.3 (10³ bp)

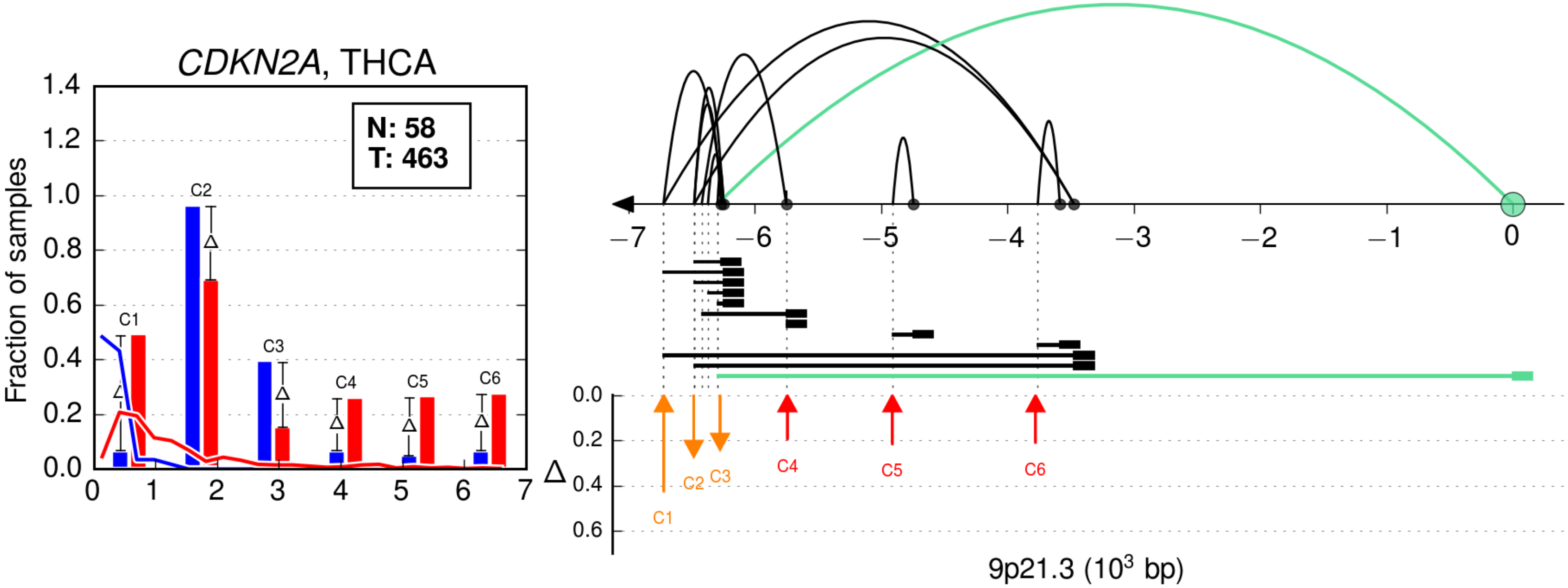

CDKN2A, THCA
N: 58
T: 463
Fraction of samples
C1
C2
C3
C4
C5
C6
Δ
9p21.3 ($10^3$ bp)

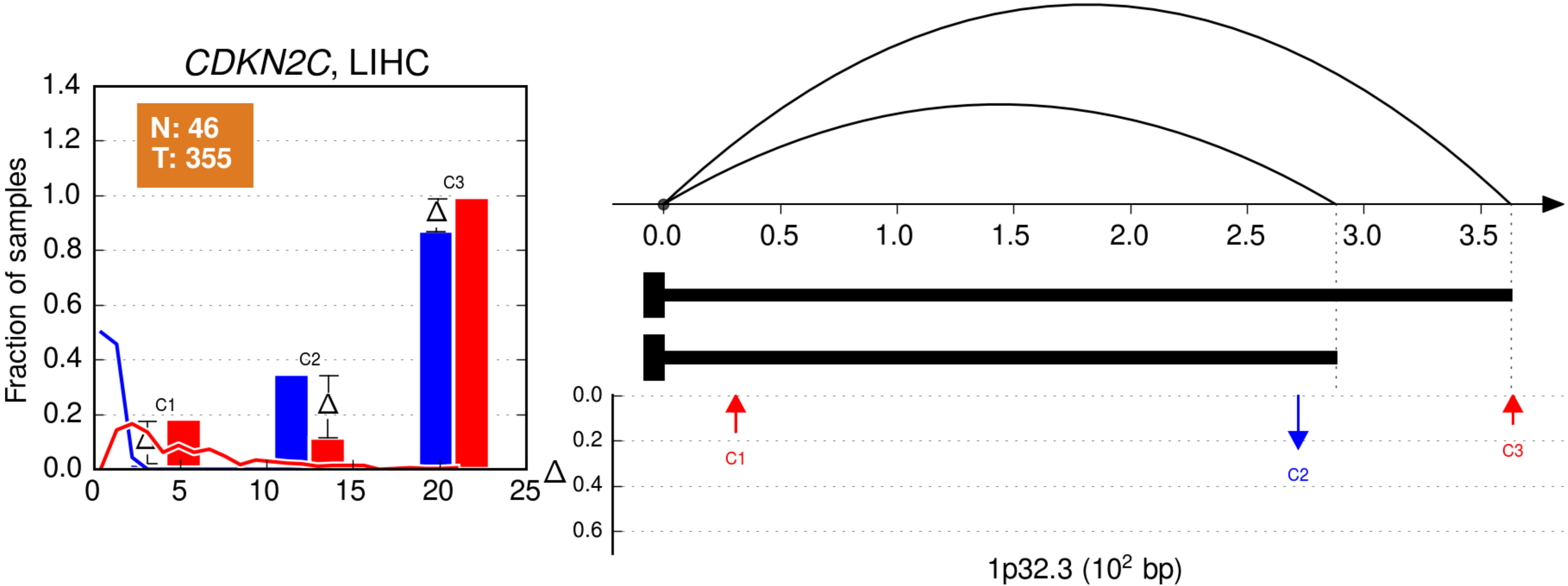

CDKN2C, LIHC
N: 46
T: 355
Fraction of samples
C1
C2
C3
Δ
1p32.3 ($10^2$ bp)
C1
C2
C3

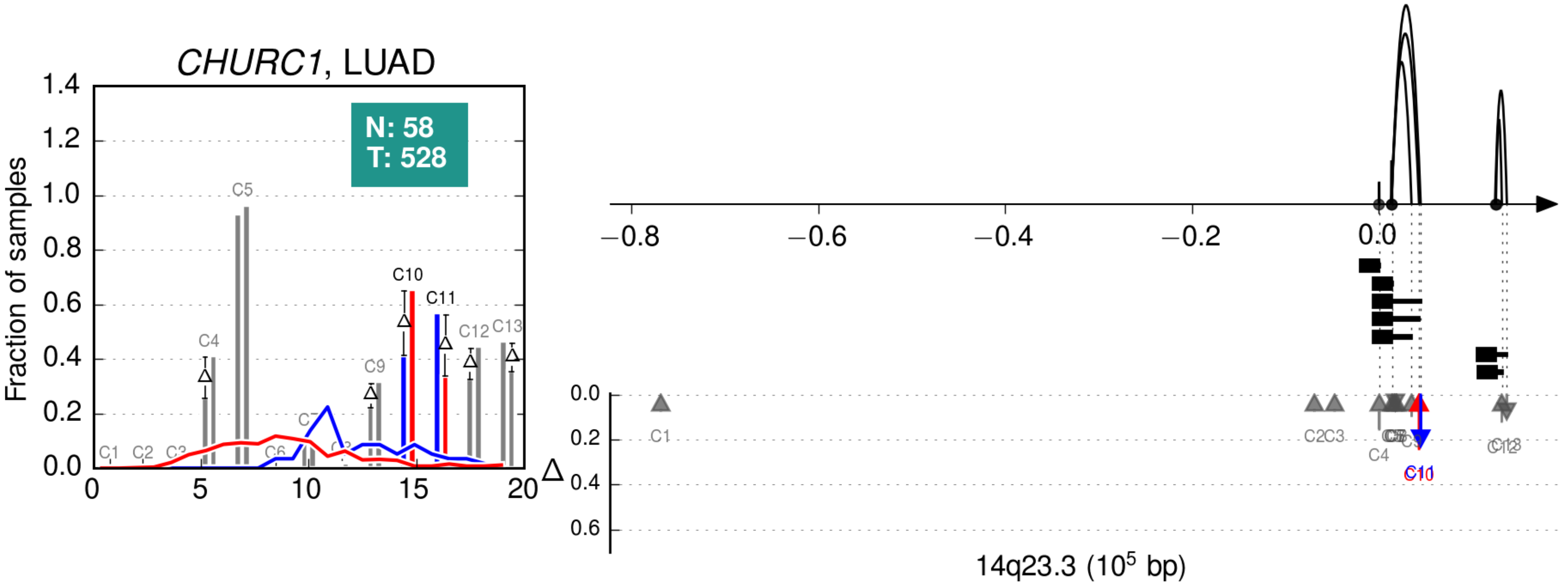

CHURC1, LUAD
N: 58
T: 528
Fraction of samples
1.4
1.2
1.0
0.8
0.6
0.4
0.2
0.0
0
5
10
15
20
C1
C2
C4
C5
C9
C10
C11
C12
C13
Δ
−0.8
−0.6
−0.4
−0.2
0.0
0.2
0.4
0.6
C2C3
C4
14q23.3 ($10^5$ bp)

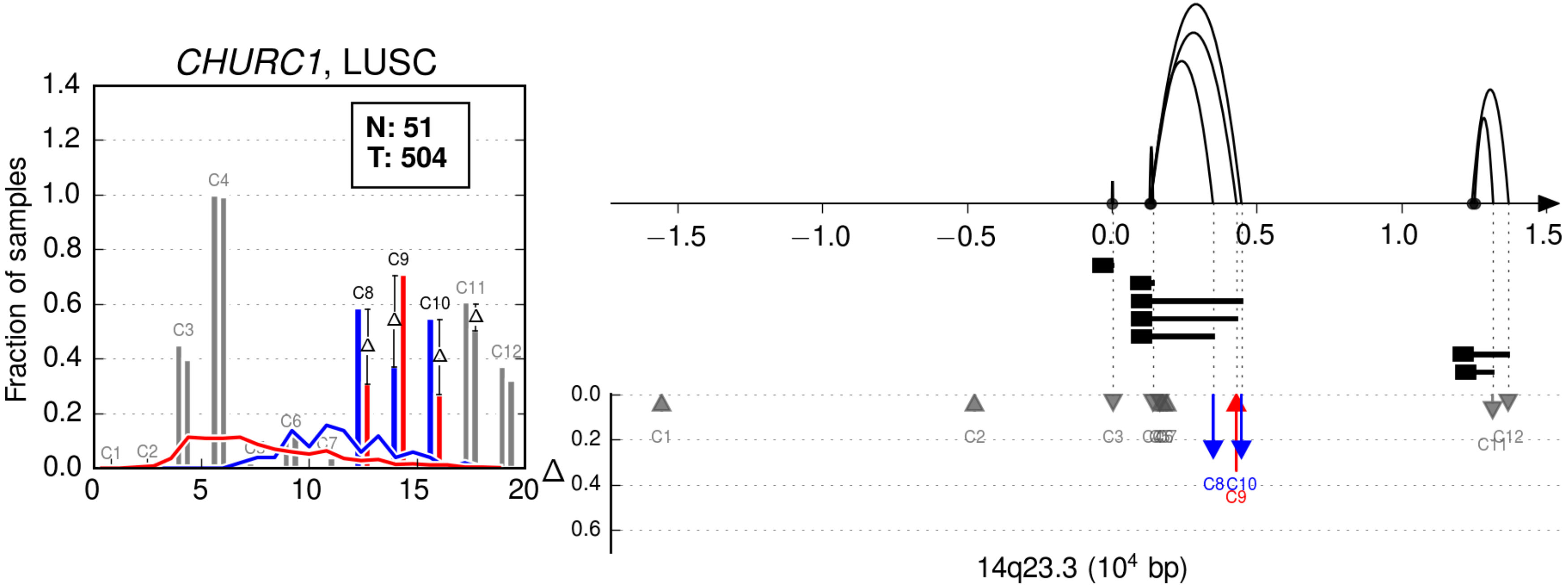

CHURC1, LUSC
N: 51
T: 504
Fraction of samples
Δ
14q23.3 (10^4 bp)

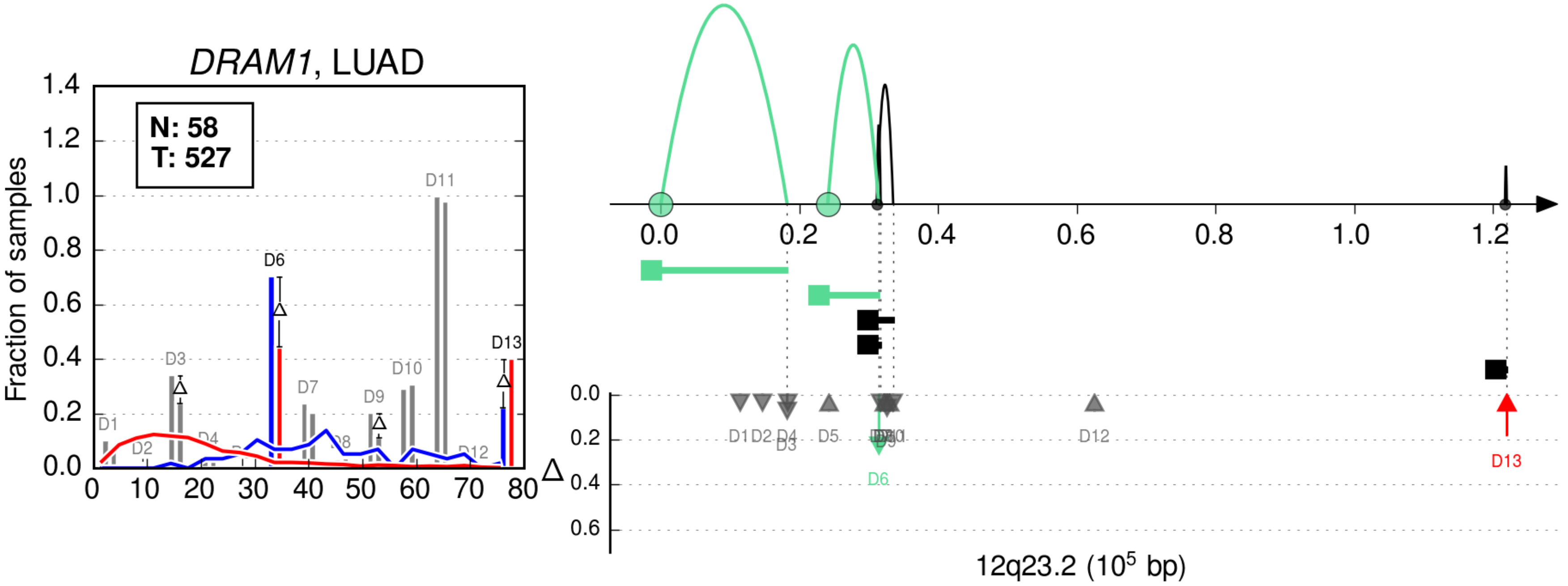

DRAM1, LUAD
N: 58
T: 527
Fraction of samples
D1
D2
D3
D4
D6
D7
D9
D10
D11
D12
D13
Δ
12q23.2 ($10^5$ bp)
D5

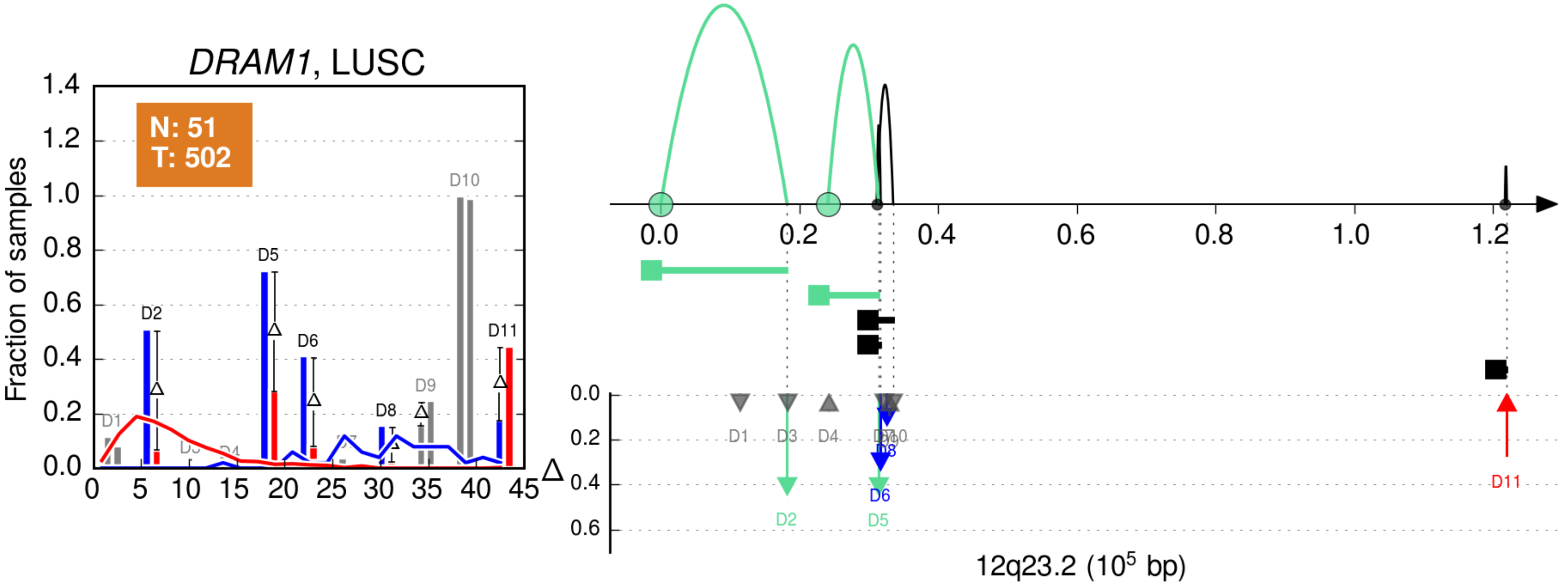

DRAM1, LUSC
N: 51
T: 502
Fraction of samples
D1
D2
D5
D6
D8
D9
D10
D11
Δ
0.0
0.2
0.4
0.6
0.8
1.0
1.2
1.4
0
5
10
15
20
25
30
35
40
45
D1
D3
D4
D2
D5
D6
D11
12q23.2 (10^5 bp)

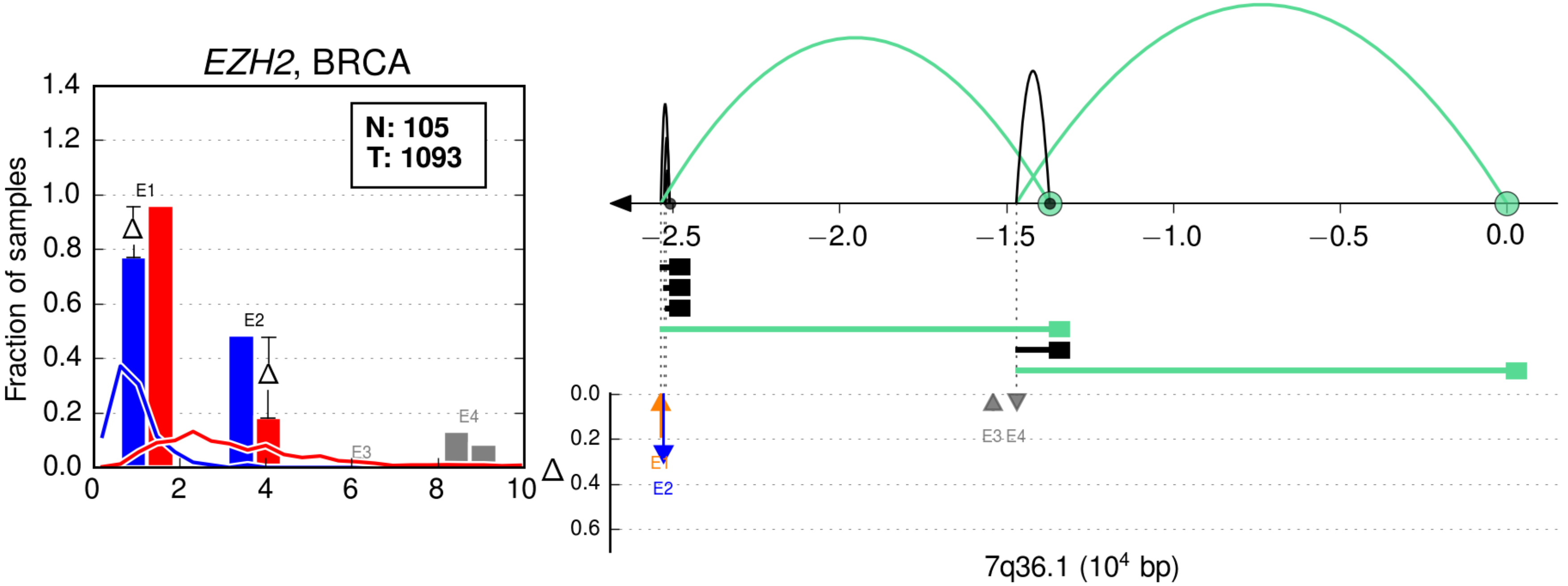

EZH2, BRCA
N: 105
T: 1093
Fraction of samples
1.4
1.2
1.0
0.8
0.6
0.4
0.2
0.0
0
2
4
6
8
10
E1
E2
E3
E4
Δ
−2.5
−2.0
−1.5
−1.0
−0.5
0.0
0.2
0.4
0.6
7q36.1 (10^4 bp)

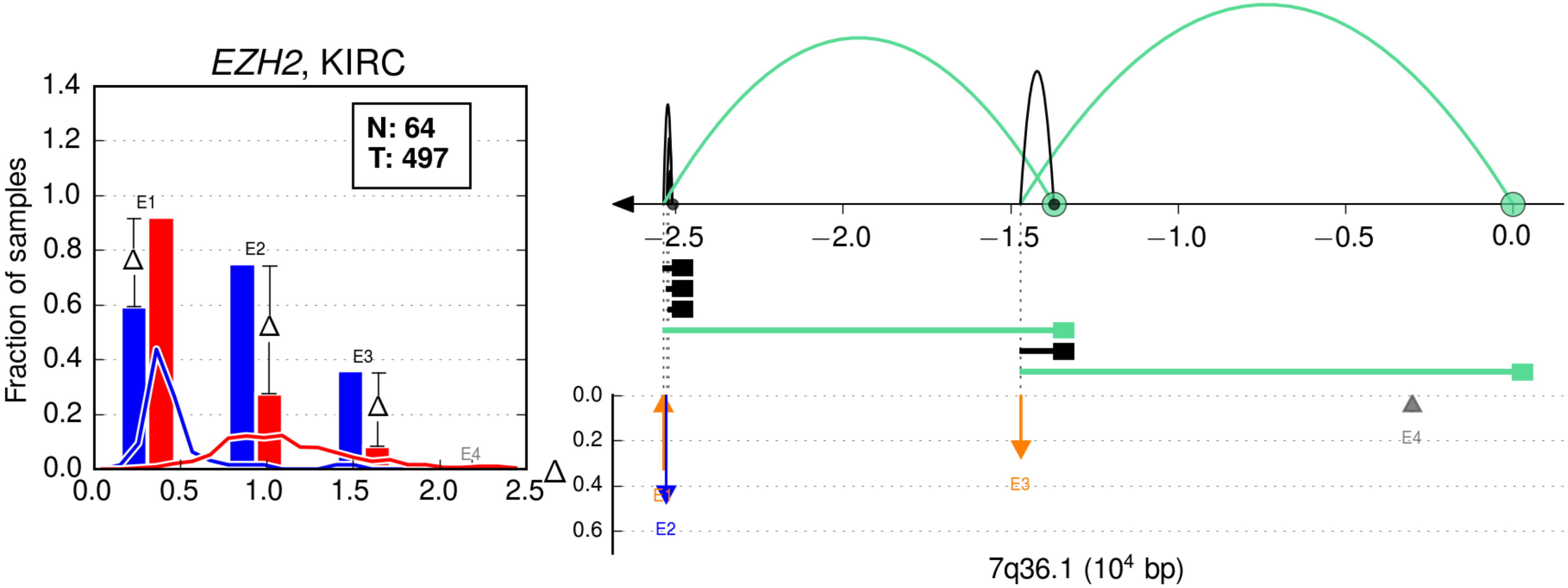

EZH2, KIRC
N: 64
T: 497
Fraction of samples
E1
E2
E3
E4
Δ
7q36.1 (10^4 bp)

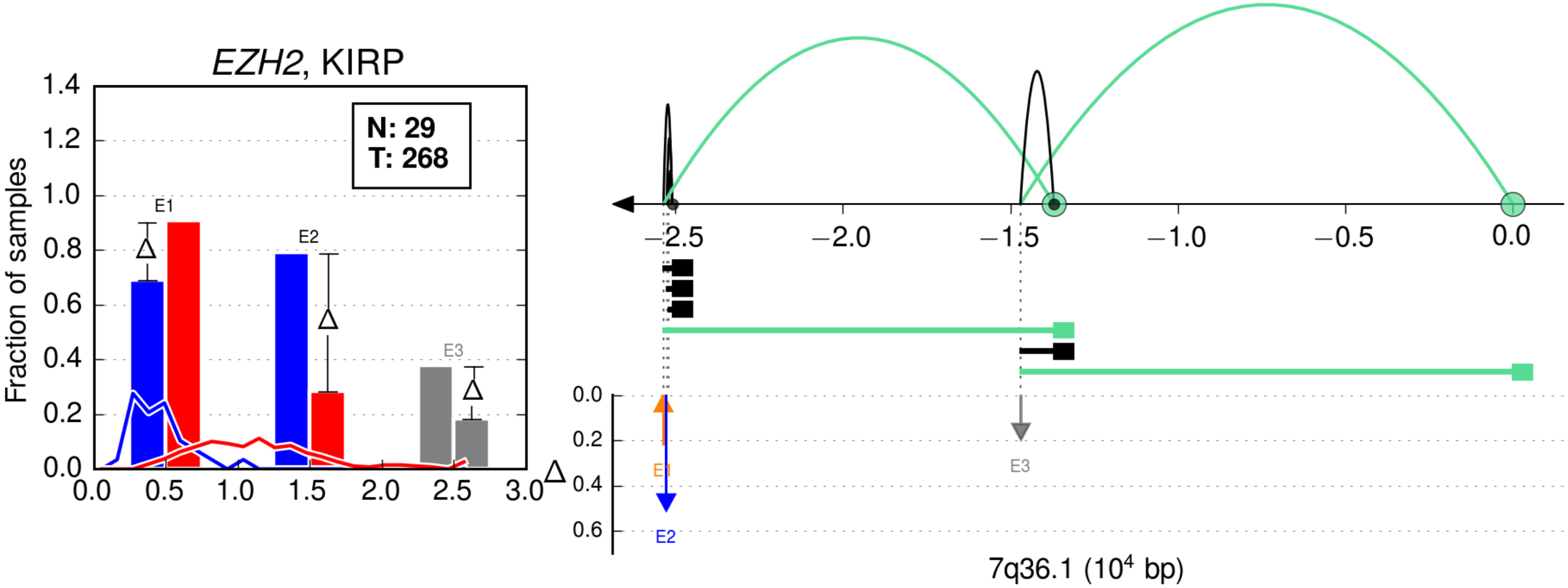

EZH2, KIRP
N: 29
T: 268
Fraction of samples
E1
E2
E3
Δ
7q36.1 ($10^4$ bp)

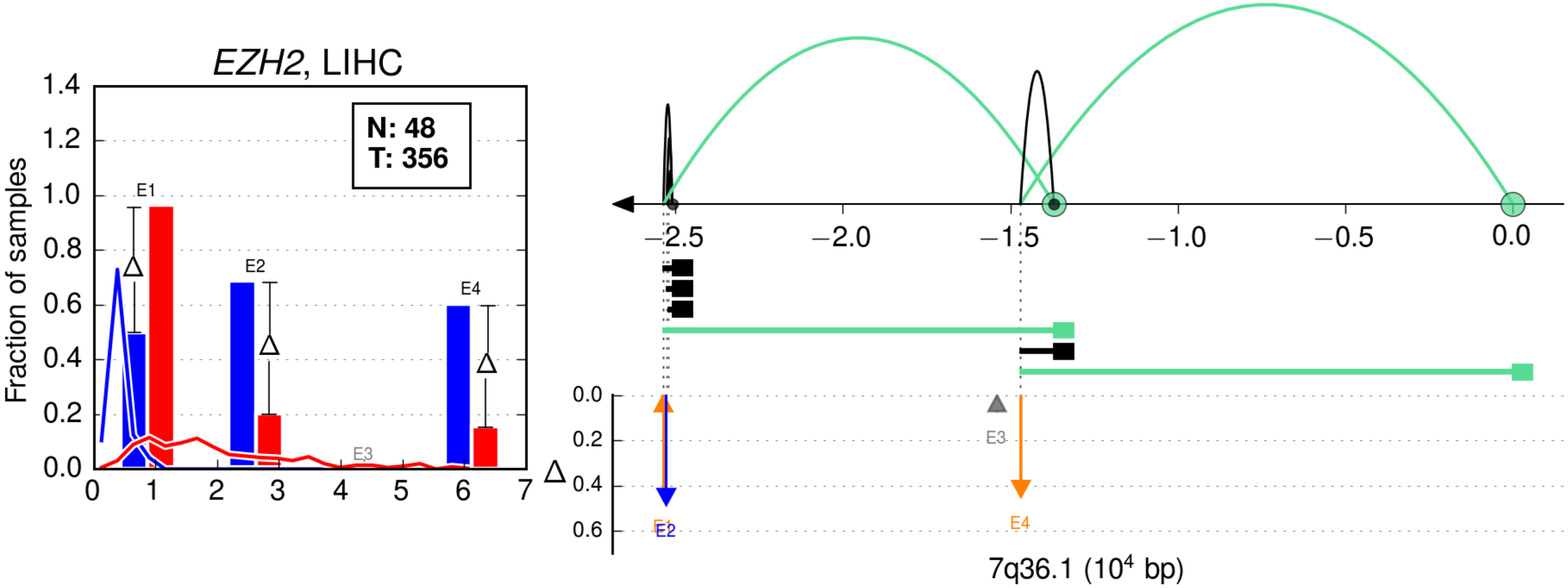

EZH2, LIHC
N: 48
T: 356
Fraction of samples
E1
E2
E3
E4
Δ
7q36.1 (10⁴ bp)

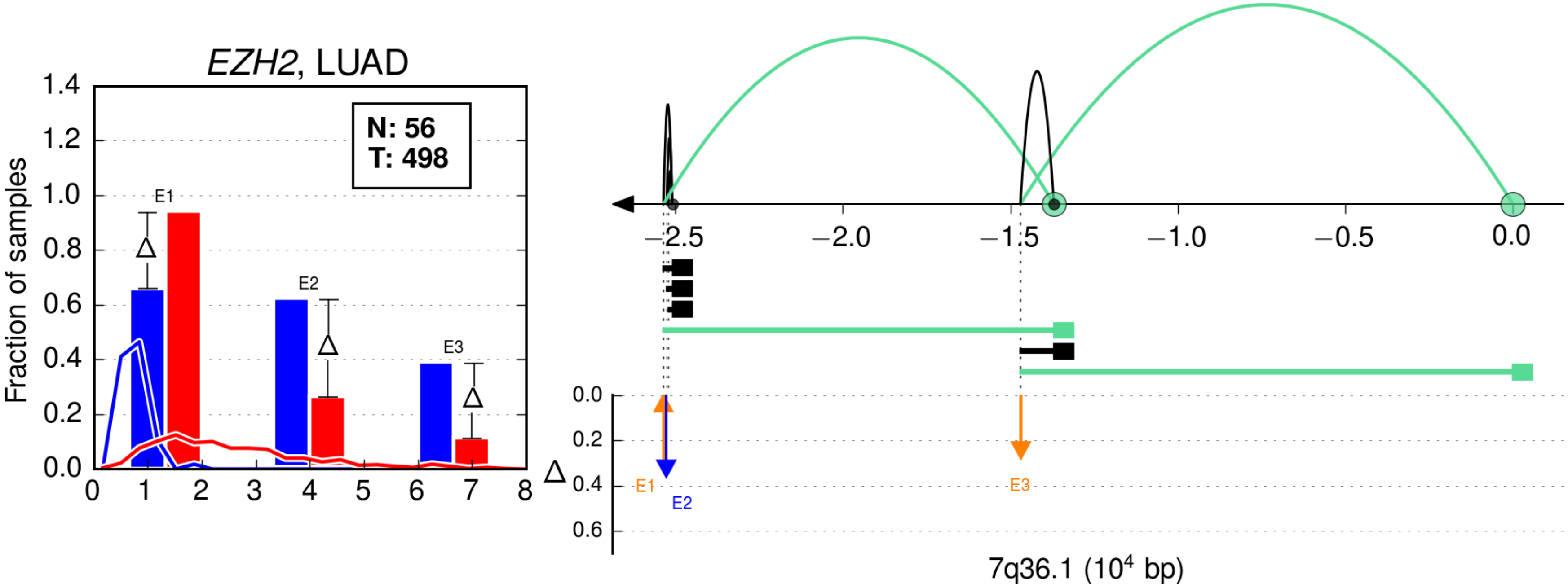

EZH2, LUAD
N: 56
T: 498
Fraction of samples
1.4
1.2
1.0
0.8
0.6
0.4
0.2
0.0
0
1
2
3
4
5
6
7
8
E1
E2
E3
Δ
−2.5
−2.0
−1.5
−1.0
−0.5
0.0
0.0
0.2
0.4
0.6
E1
E2
E3
7q36.1 (10⁴ bp)

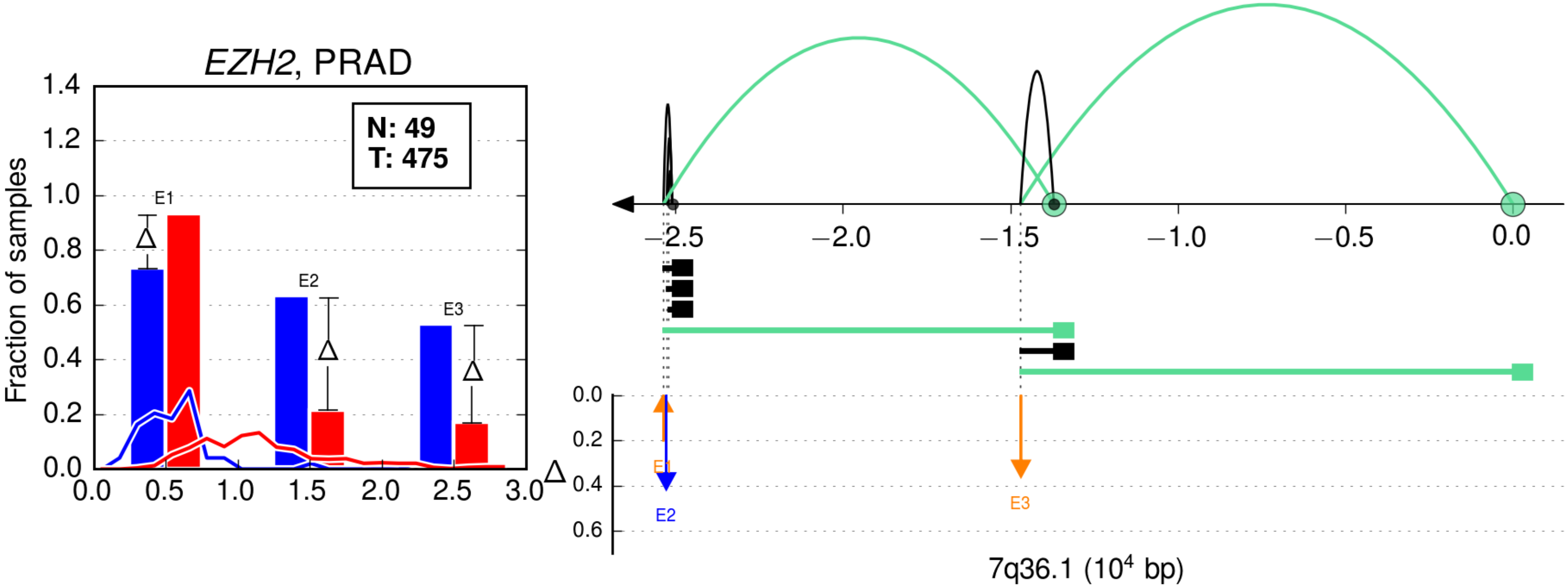

EZH2, PRAD
N: 49
T: 475
Fraction of samples
E1
E2
E3
Δ
7q36.1 ($10^4$ bp)

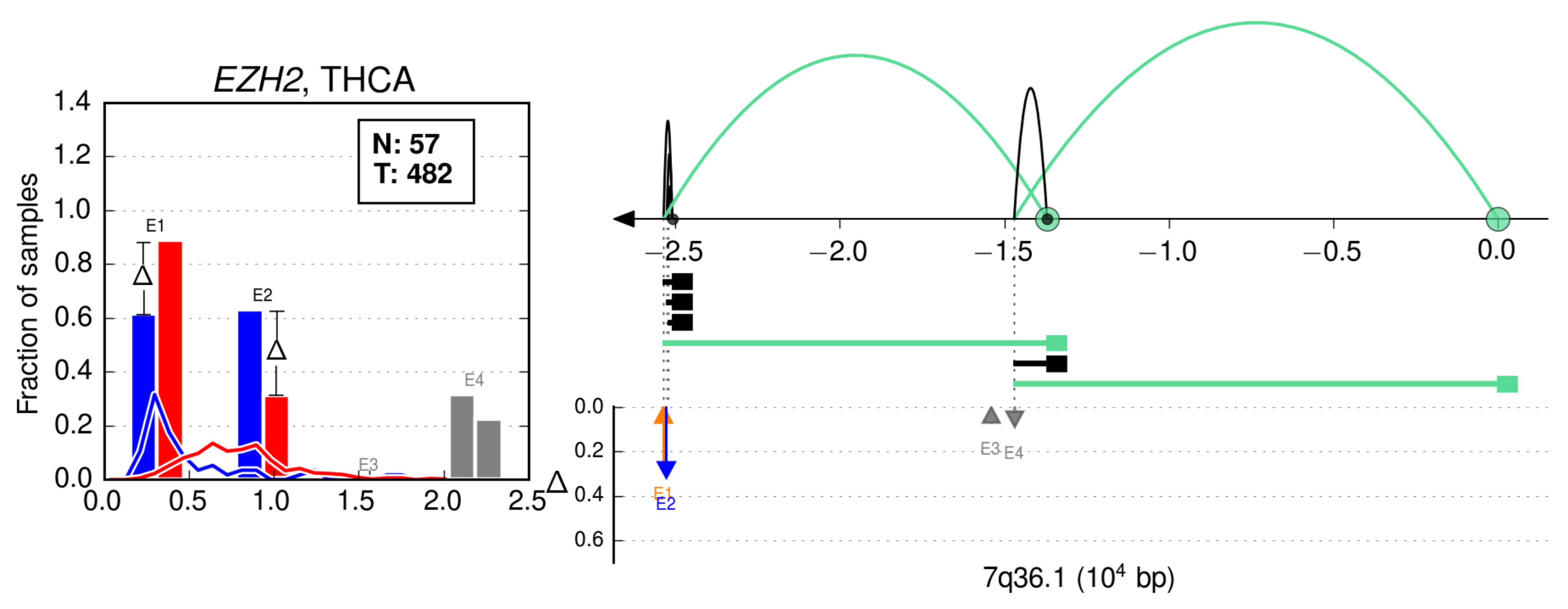

EZH2, THCA
N: 57
T: 482
Fraction of samples
E1
E2
E3
E4
Δ
7q36.1 (10^4 bp)

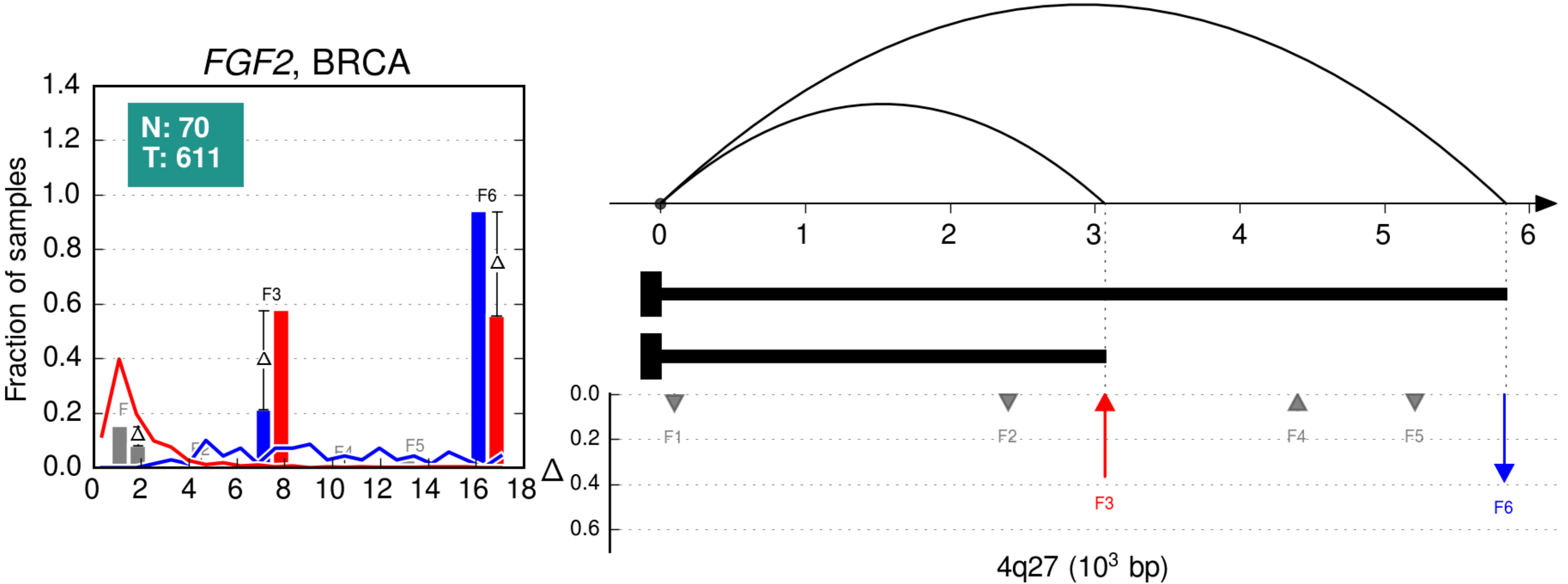

FGF2, BRCA
N: 70
T: 611
Fraction of samples
F3
F6
F5
Δ
4q27 ($10^3$ bp)
F1
F2
F3
F4
F5
F6

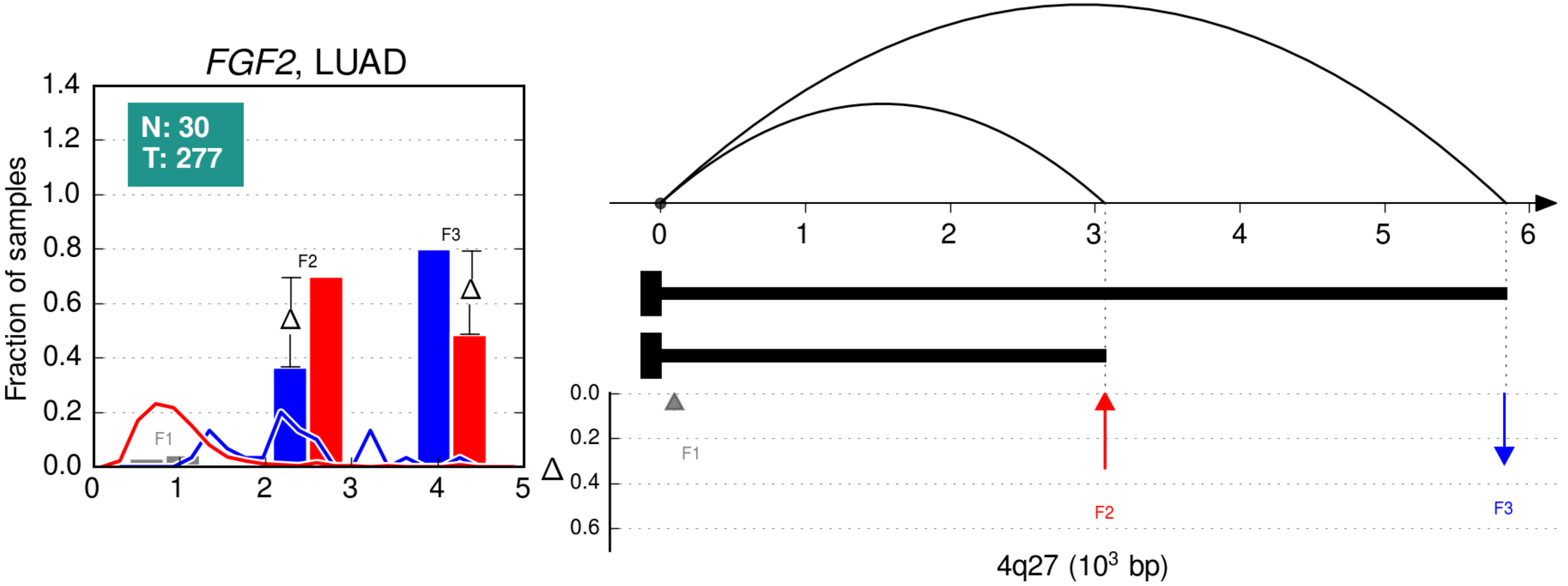
FGF2, LUAD
N: 30
T: 277
Fraction of samples
1.4
1.2
1.0
0.8
0.6
0.4
0.2
0.0
0
1
2
3
4
5
F1
F2
F3
Δ
0
1
2
3
4
5
6
0.0
0.2
0.4
0.6
F1
F2
F3
4q27 (10³ bp)

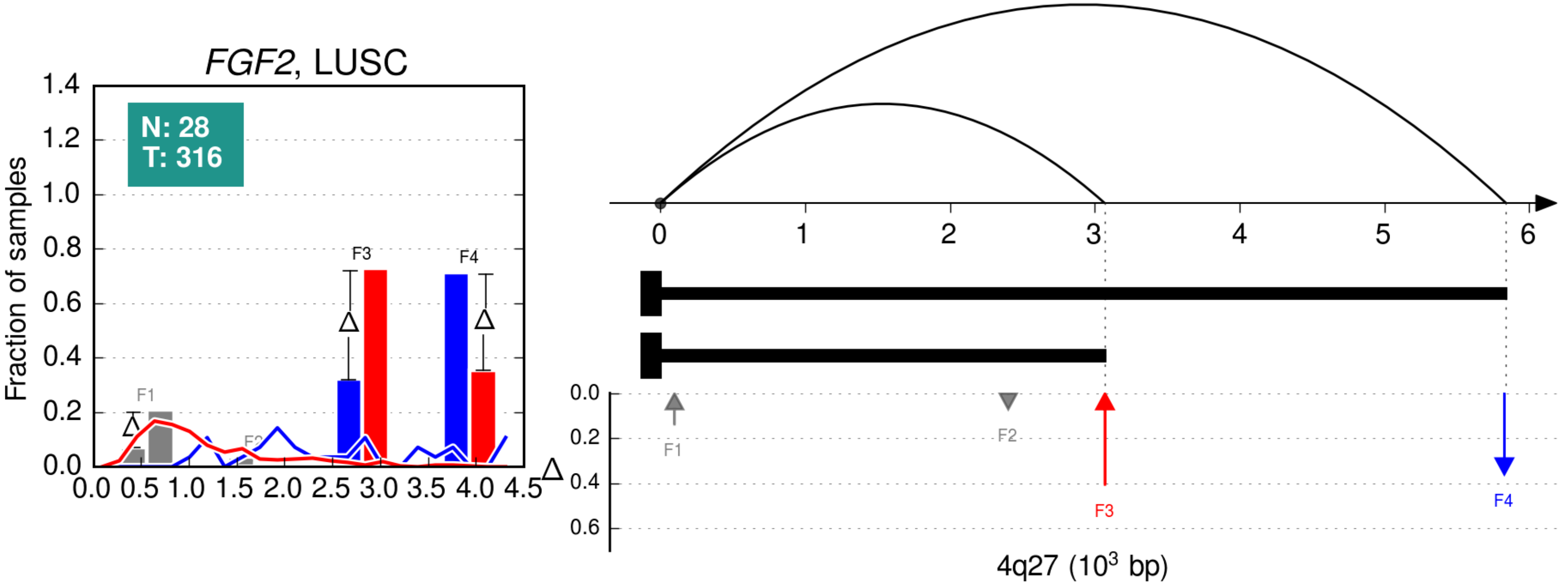

FGF2, LUSC
N: 28
T: 316
Fraction of samples
F1
F2
F3
F4
Δ
0.0 0.5 1.0 1.5 2.0 2.5 3.0 3.5 4.0 4.5
0.0 0.2 0.4 0.6 0.8 1.0 1.2 1.4
0 1 2 3 4 5 6
0.0 0.2 0.4 0.6
4q27 ($10^3$ bp)

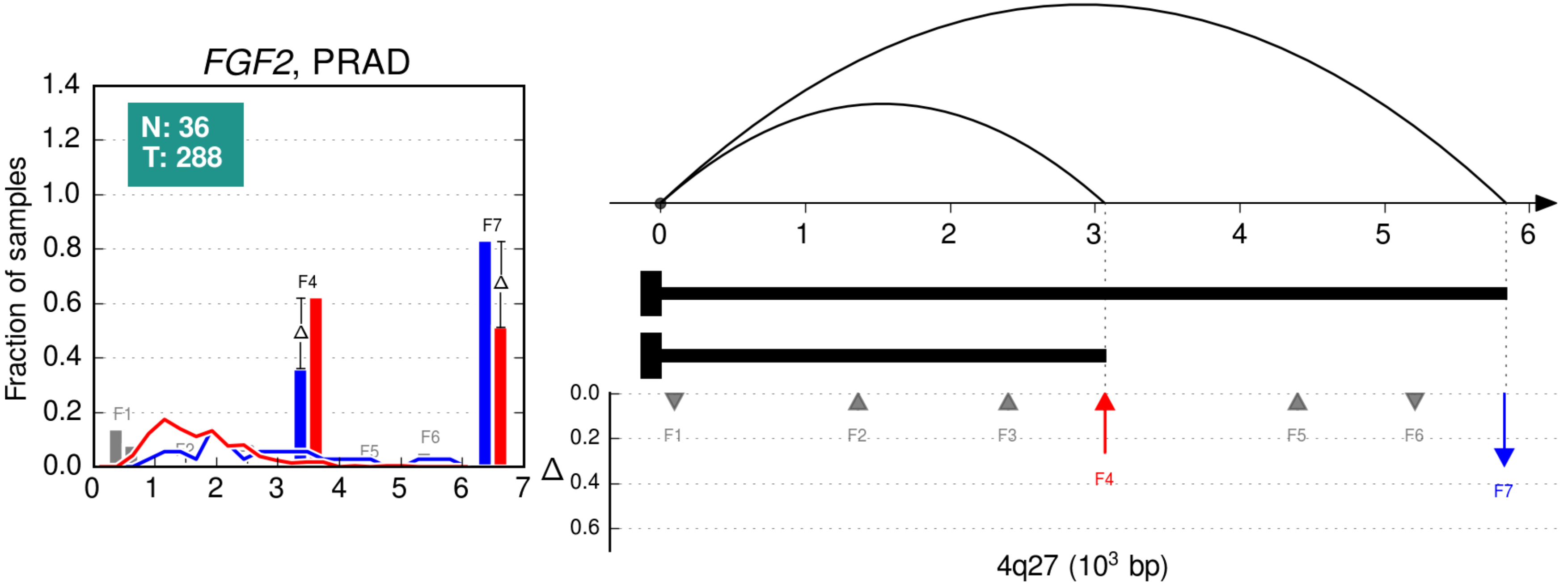

FGF2, PRAD
N: 36
T: 288
Fraction of samples
1.4
1.2
1.0
0.8
0.6
0.4
0.2
0.0
0 1 2 3 4 5 6 7
F1
F4
F5
F6
F7
Δ
0 1 2 3 4 5 6
Δ
0.0
0.2
0.4
0.6
F1
F2
F3
F4
F5
F6
F7
4q27 (10³ bp)

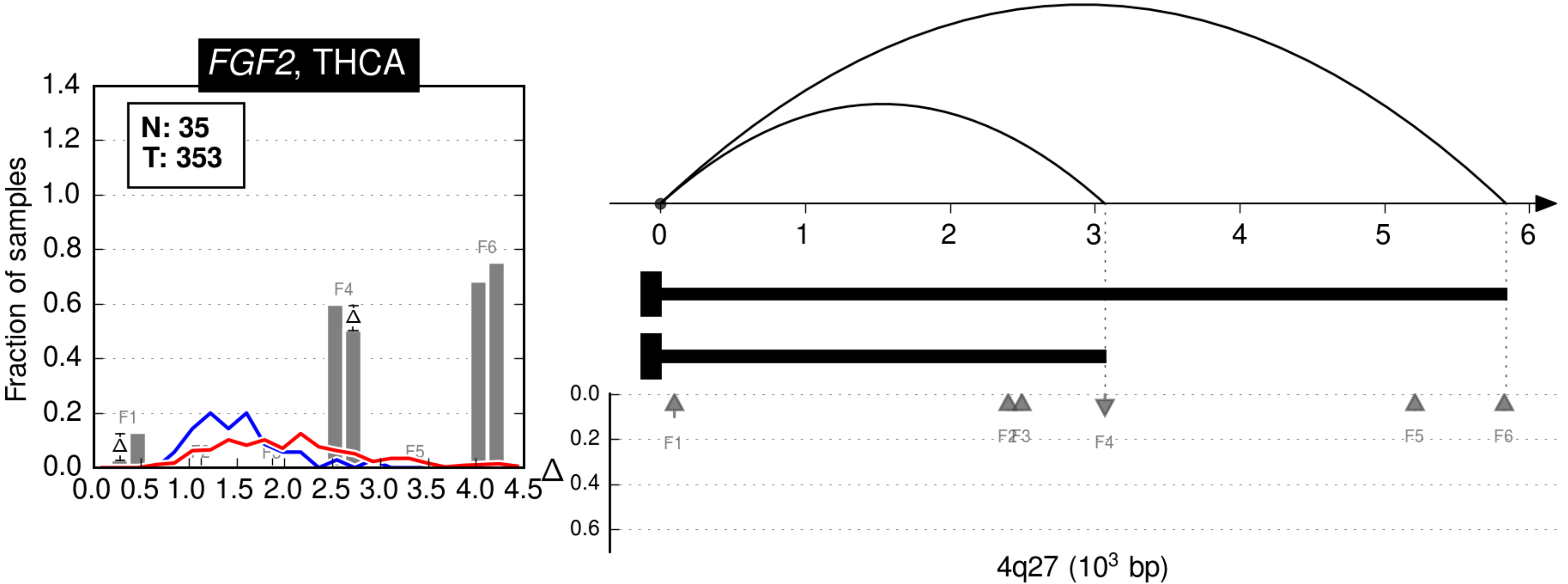

FGF2, THCA
N: 35
T: 353
Fraction of samples
F1
F4
F5
F6
Δ
4q27 (10³ bp)
F1
F2 F3
F4
F5
F6

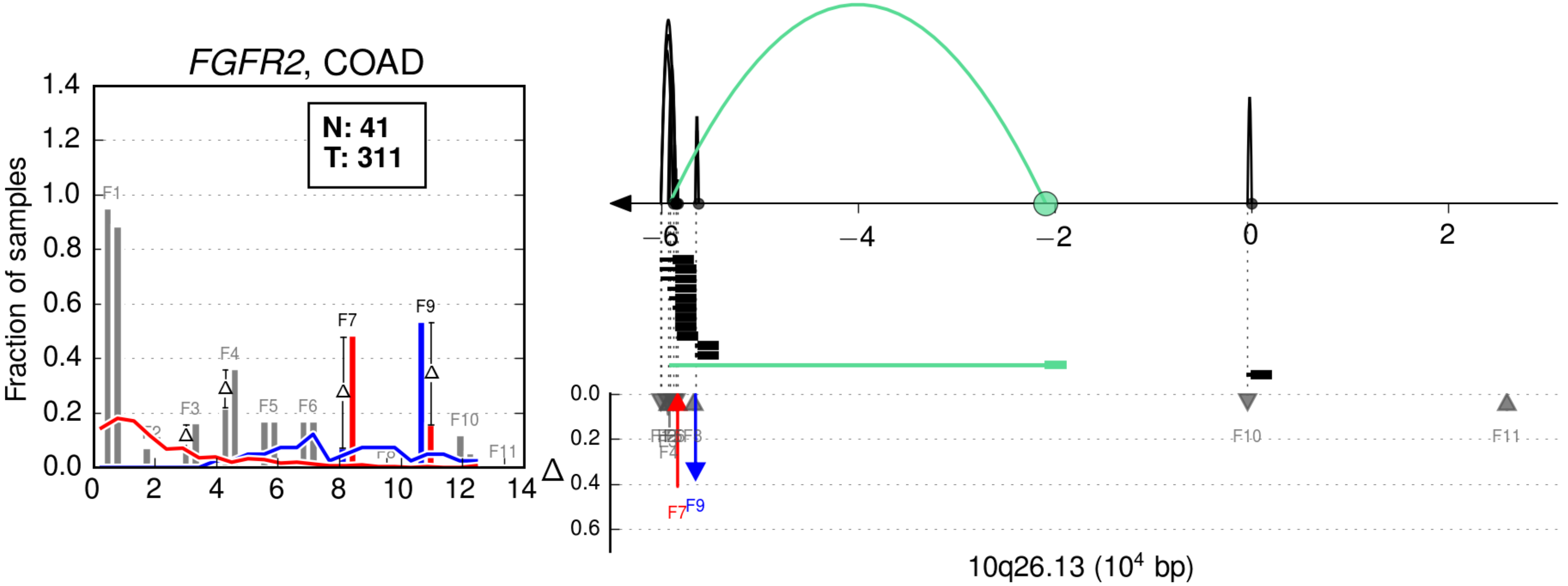

FGFR2, COAD
N: 41
T: 311
Fraction of samples
F1
F3
F4
F5
F6
F7
F9
F10
F11
10q26.13 ($10^4$ bp)
Δ

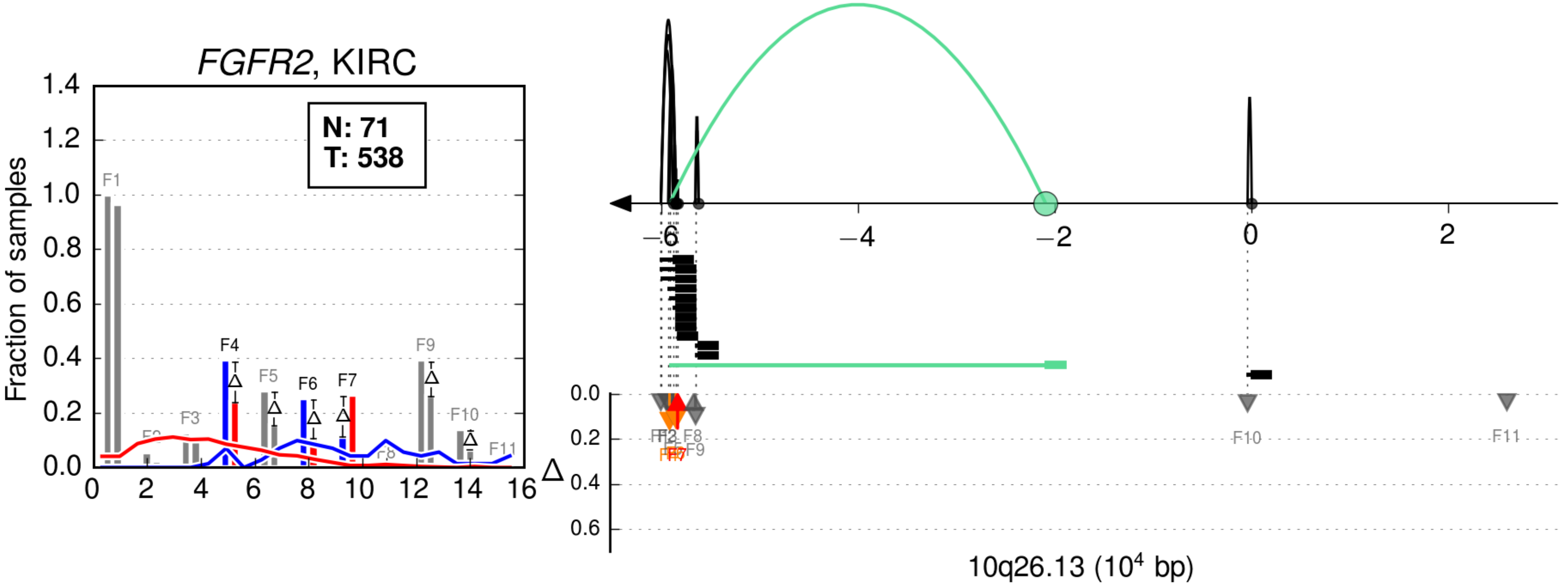

FGFR2, KIRC
N: 71
T: 538
Fraction of samples
F1
F3
F4
F5
F6
F7
F9
F10
F11
Δ
10q26.13 (10^4 bp)
F8
F9
F10
F11

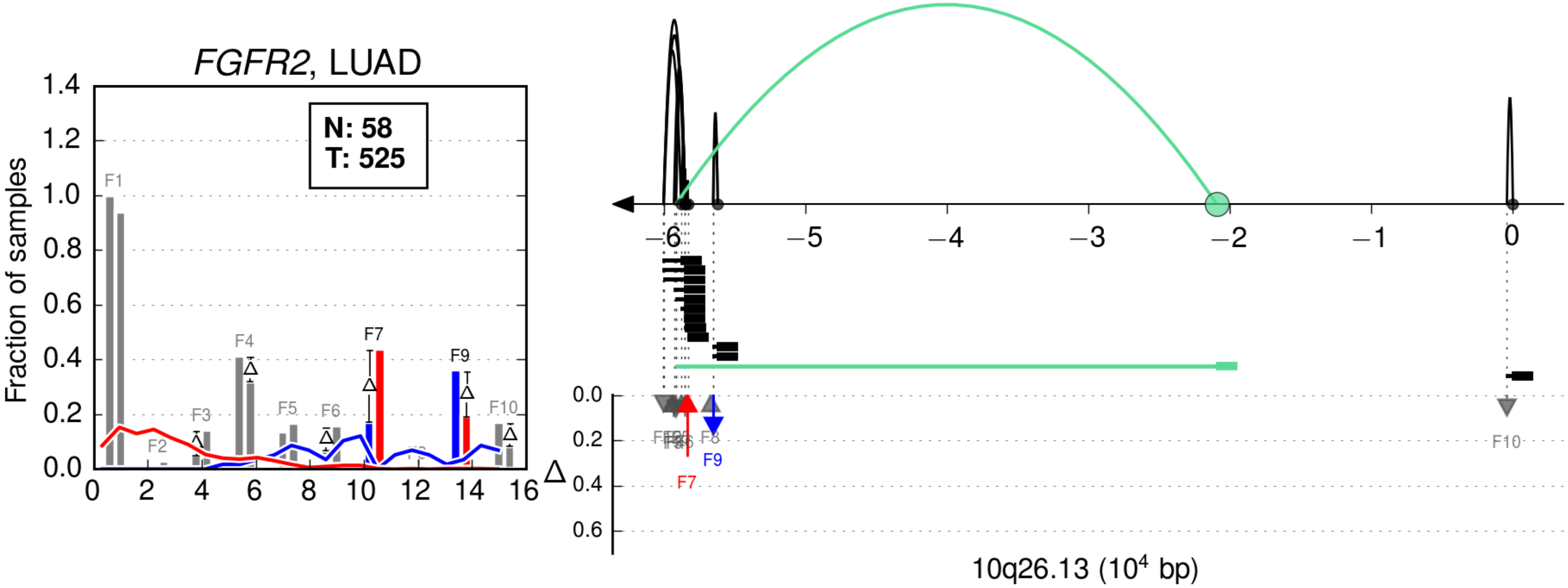

FGFR2, LUAD
N: 58
T: 525
Fraction of samples
F1
F2
F3
F4
F5
F6
F7
F9
F10
Δ
10q26.13 (10^4 bp)

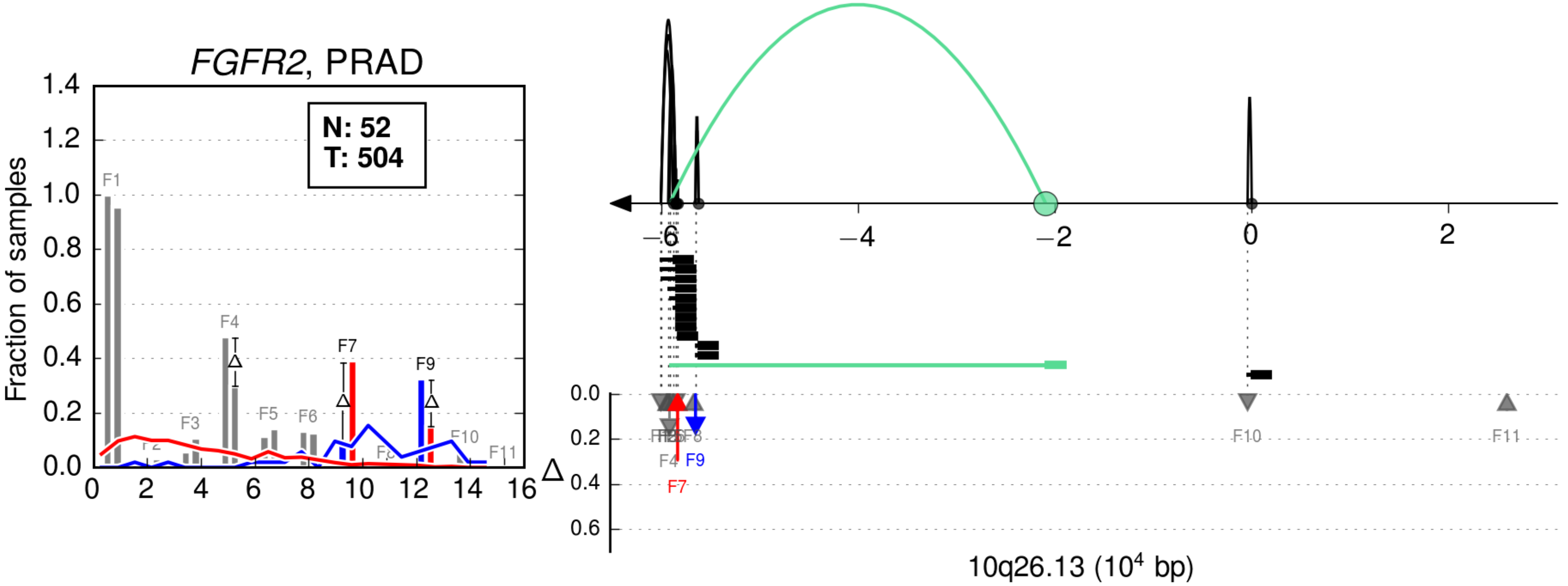

FGFR2, PRAD
N: 52
T: 504
Fraction of samples
F1
F3
F4
F5
F6
F7
F9
F10
F11
Δ
10q26.13 ($10^4$ bp)

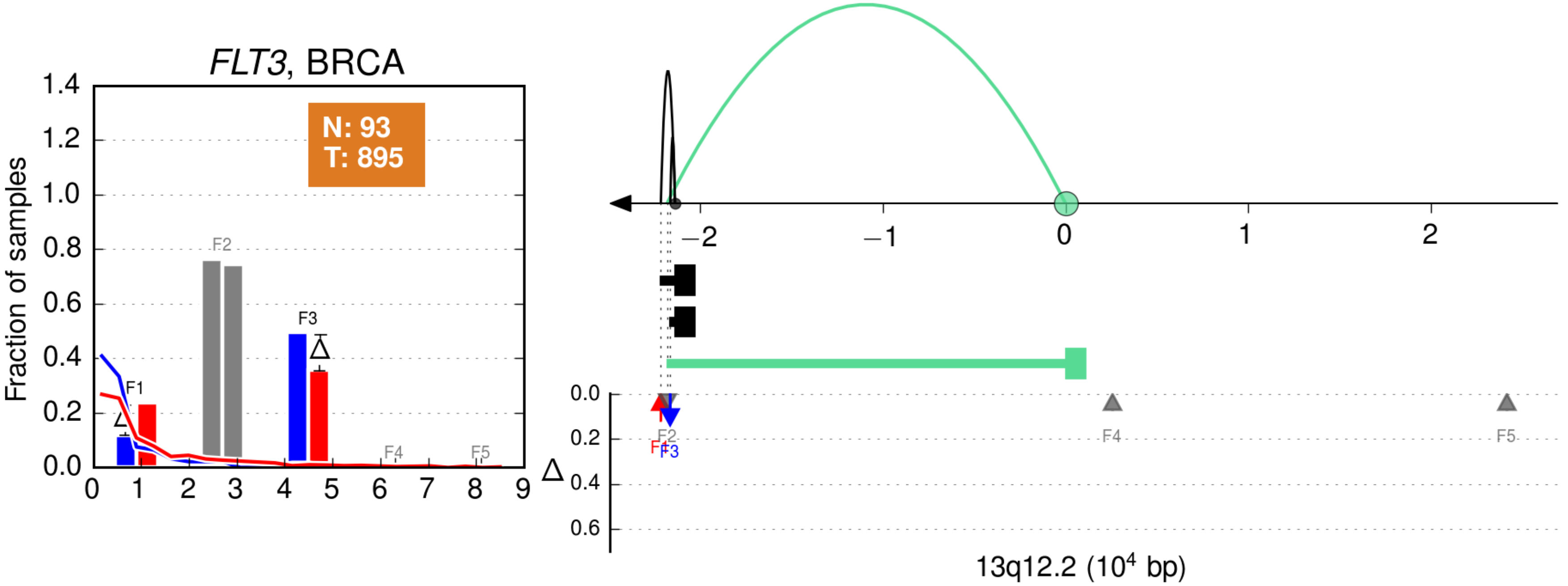

FLT3, BRCA
N: 93
T: 895
Fraction of samples
1.4
1.2
1.0
0.8
0.6
0.4
0.2
0.0
0 1 2 3 4 5 6 7 8 9
F1
F2
F3
F4
F5
Δ
−2 −1 0 1 2
0.0
0.2
0.4
0.6
F1
F2
F3
F4
F5
13q12.2 ($10^4$ bp)

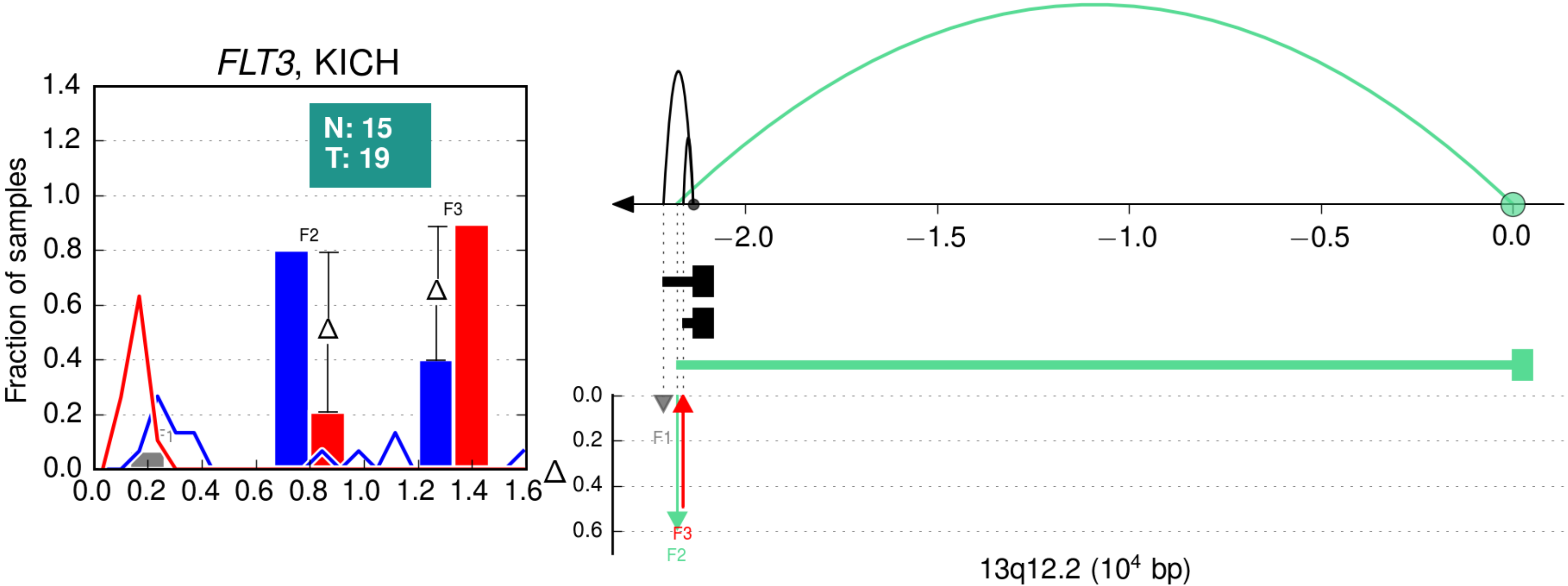

FLT3, KICH
N: 15
T: 19
Fraction of samples
F1
F2
F3
Δ
−2.0
−1.5
−1.0
−0.5
0.0
13q12.2 ($10^4$ bp)

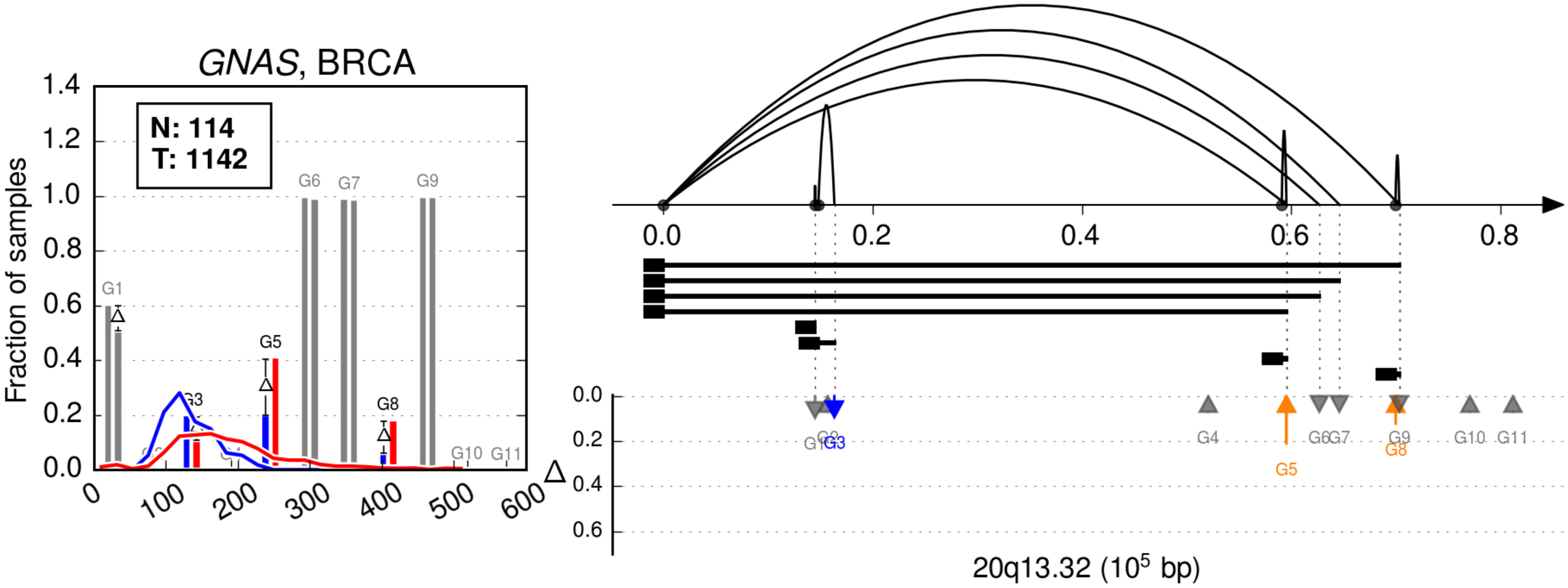

*GNAS*, BRCA
N: 114
T: 1142
Fraction of samples
G1
G3
G5
G6
G7
G8
G9
G10
G11
Δ
20q13.32 ($10^5$ bp)
G4
G6G7
G9
G8
G5
G10
G11
G3

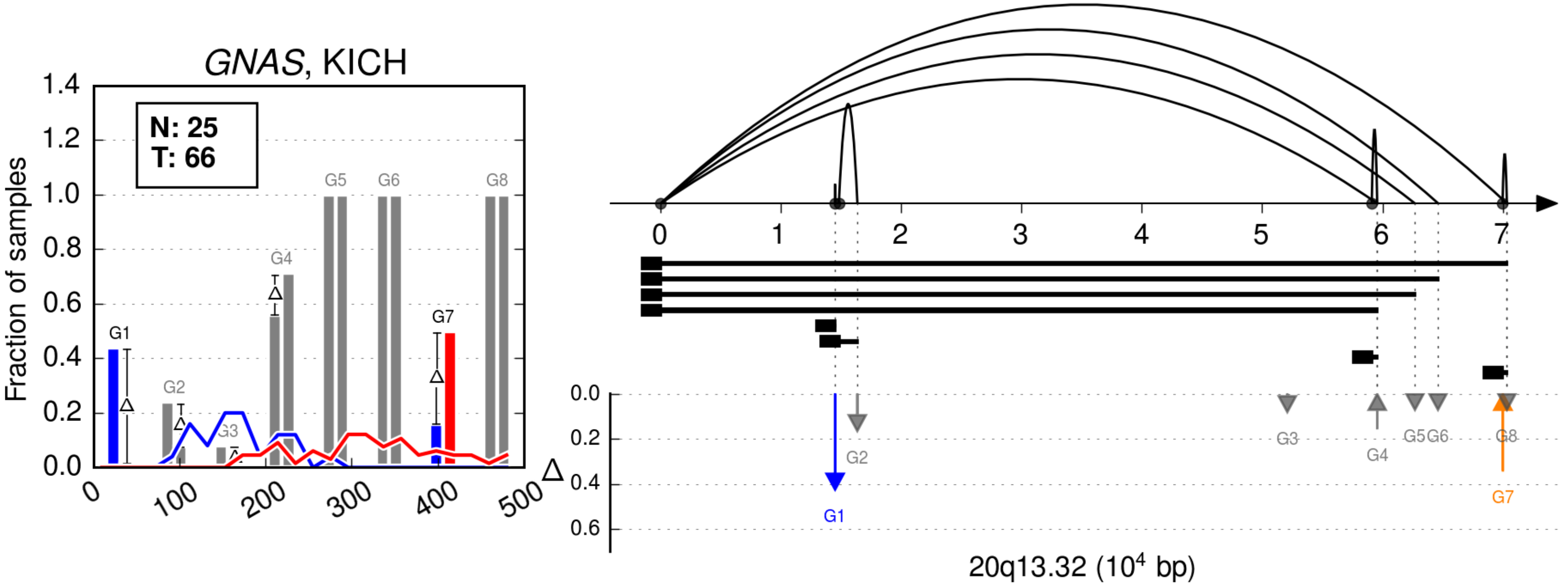

GNAS, KICH
N: 25
T: 66
Fraction of samples
G1
G2
G3
G4
G5
G6
G7
G8
Δ
20q13.32 (10⁴ bp)

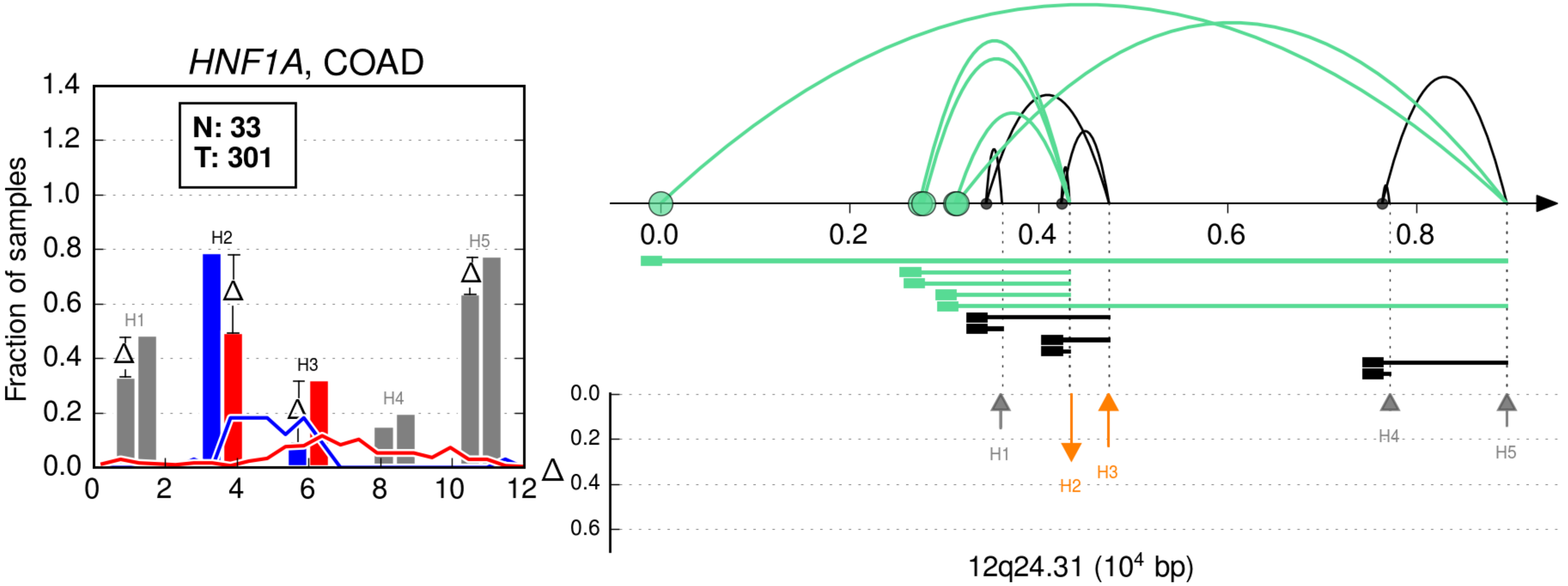

HNF1A, COAD
N: 33
T: 301
Fraction of samples
H1
H2
H3
H4
H5
Δ
12q24.31 (10⁴ bp)

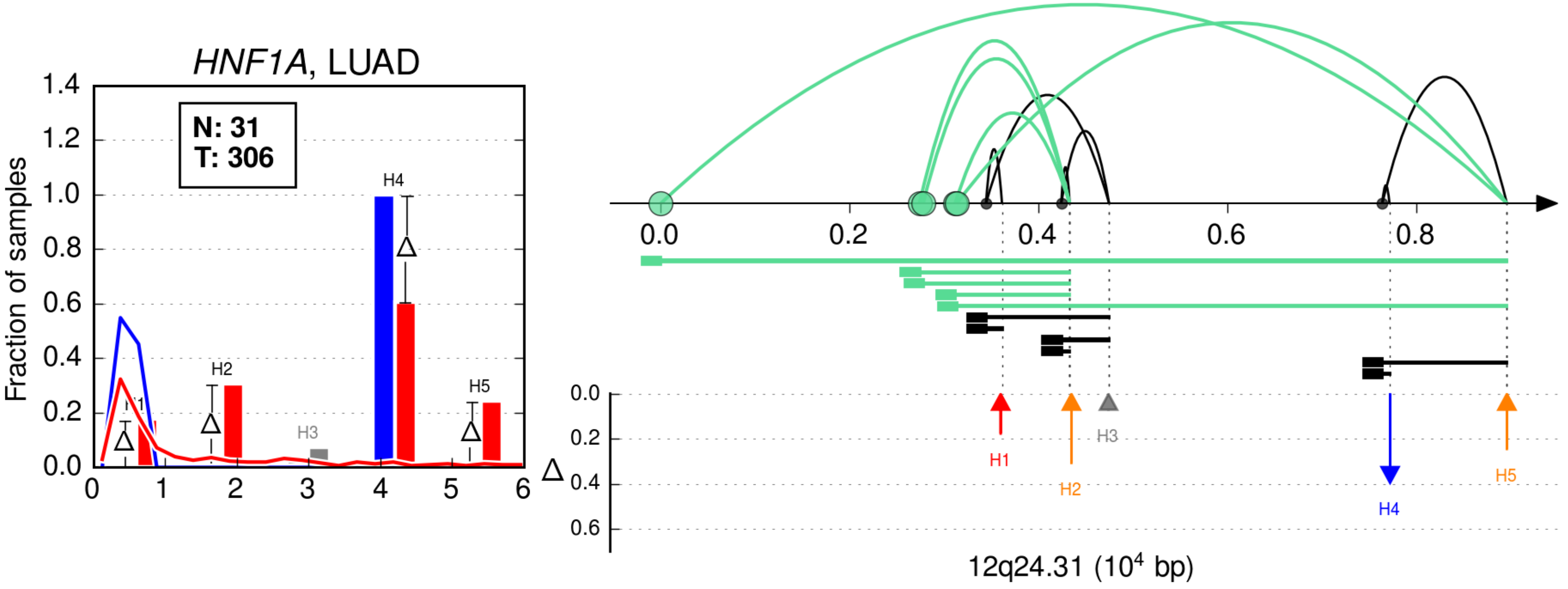
HNF1A, LUAD
N: 31
T: 306
Fraction of samples
H1
H2
H3
H4
H5
Δ
12q24.31 ($10^4$ bp)

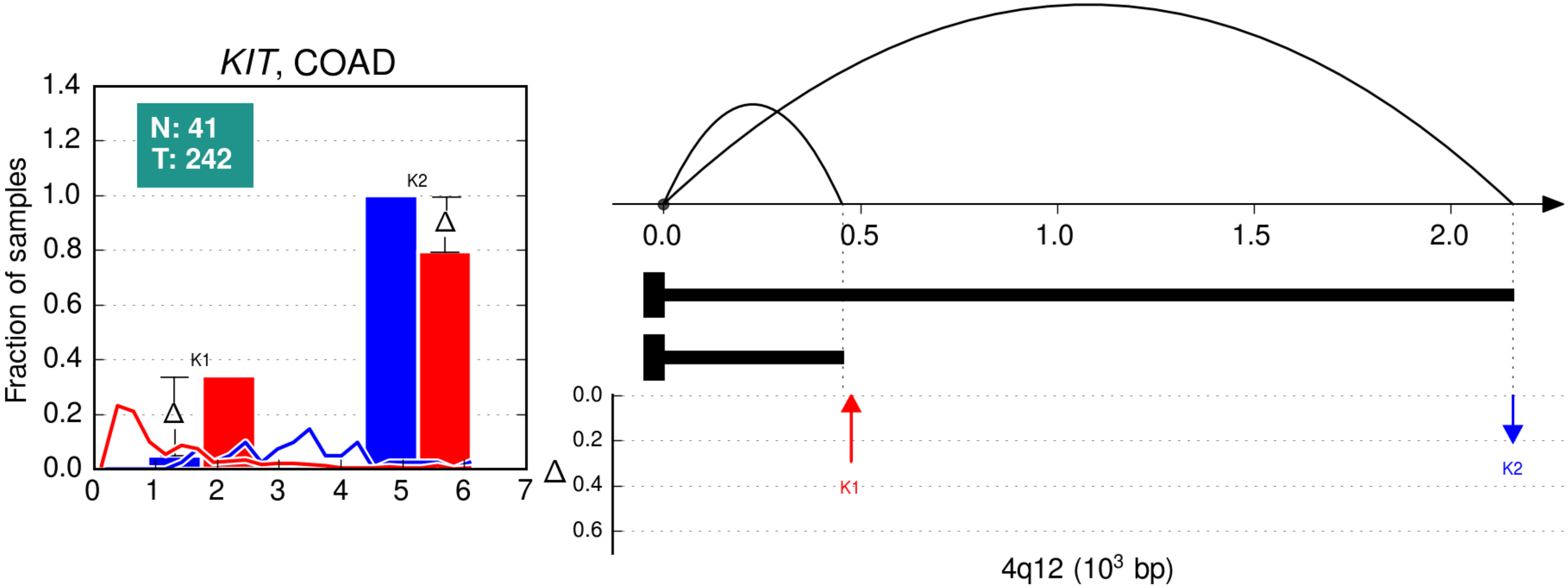

KIT, COAD
N: 41
T: 242
Fraction of samples
K1
K2
Δ
4q12 ($10^3$ bp)

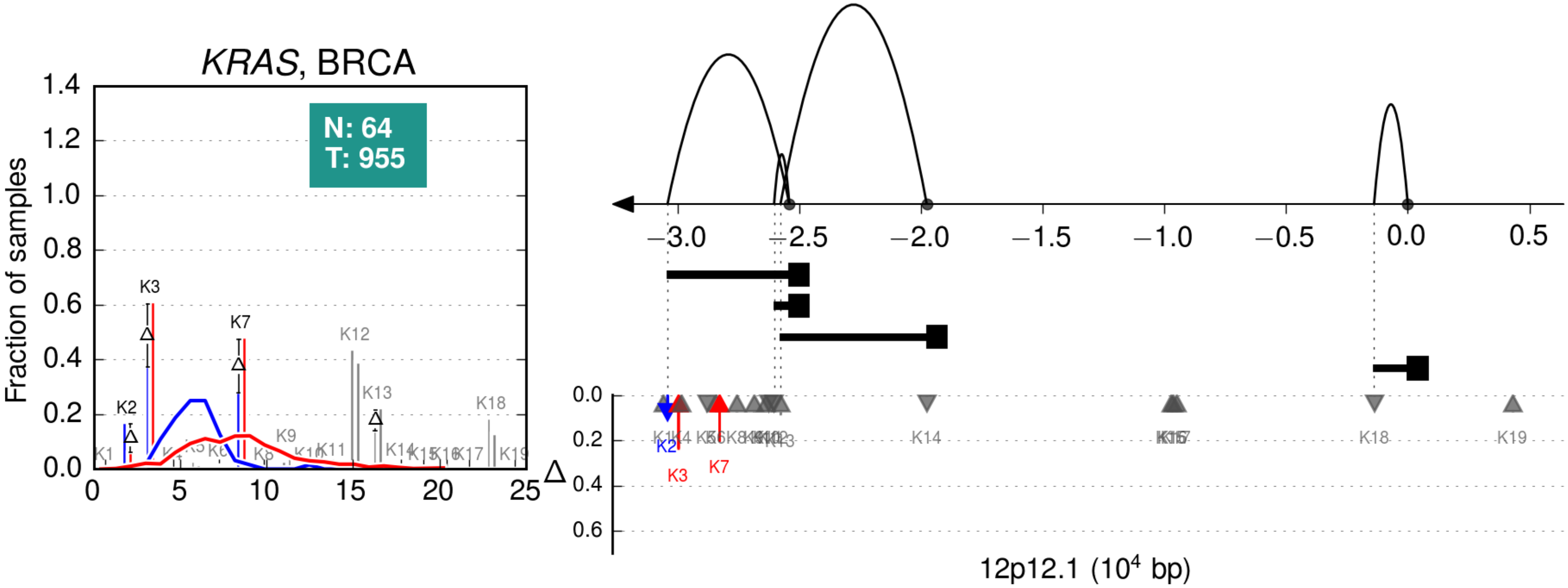

KRAS, BRCA
N: 64
T: 955
Fraction of samples
K2
K3
K7
K9
K11
K12
K13
K18
K19
Δ
12p12.1 ($10^4$ bp)

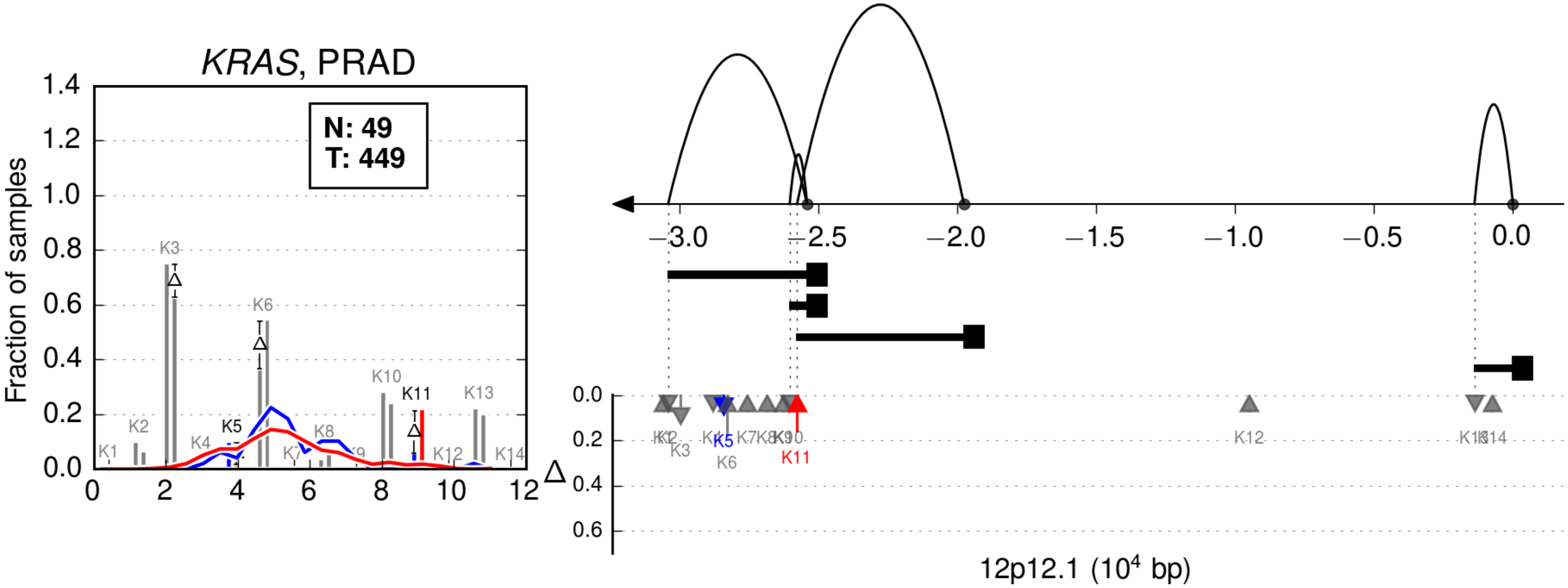

KRAS, PRAD
N: 49
T: 449
Fraction of samples
K1
K2
K3
K4
K5
K6
K7
K8
K10
K11
K12
K13
K14
Δ
12p12.1 (10^4 bp)

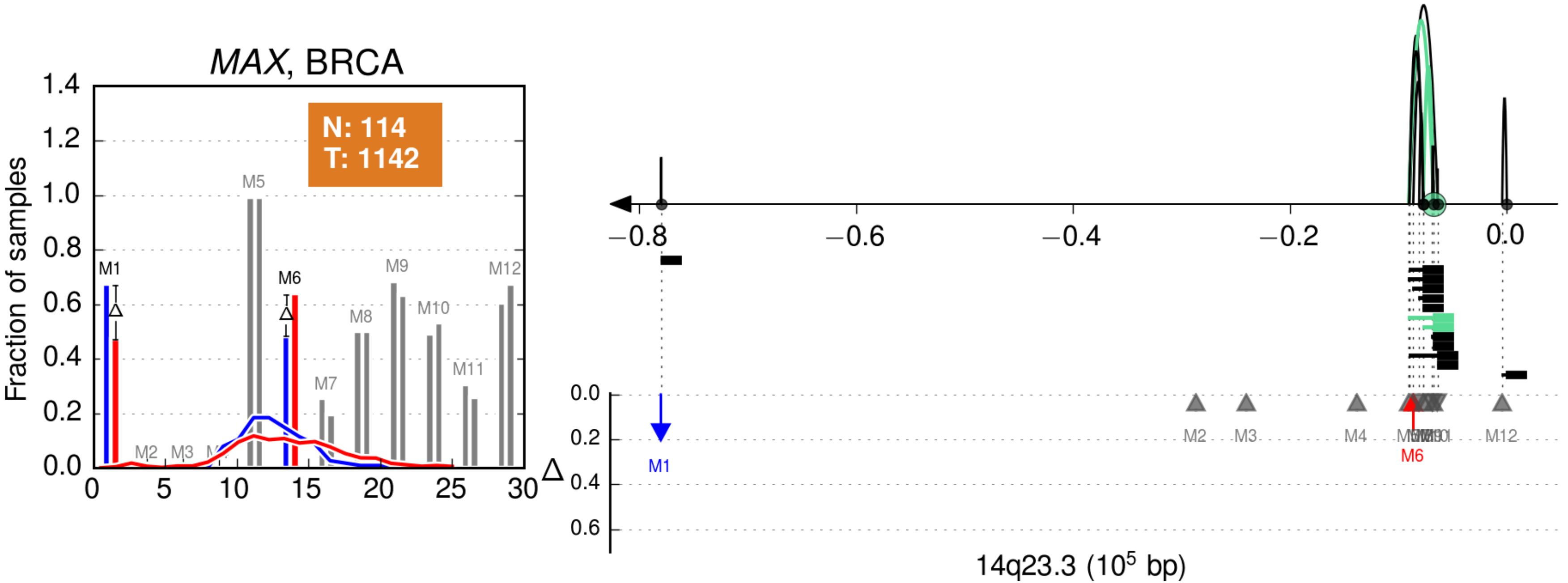

MAX, BRCA
N: 114
T: 1142
Fraction of samples
M1
M2
M3
M5
M6
M7
M8
M9
M10
M11
M12
Δ
M1
M6
M2
M3
M4
M12
14q23.3 ($10^5$ bp)

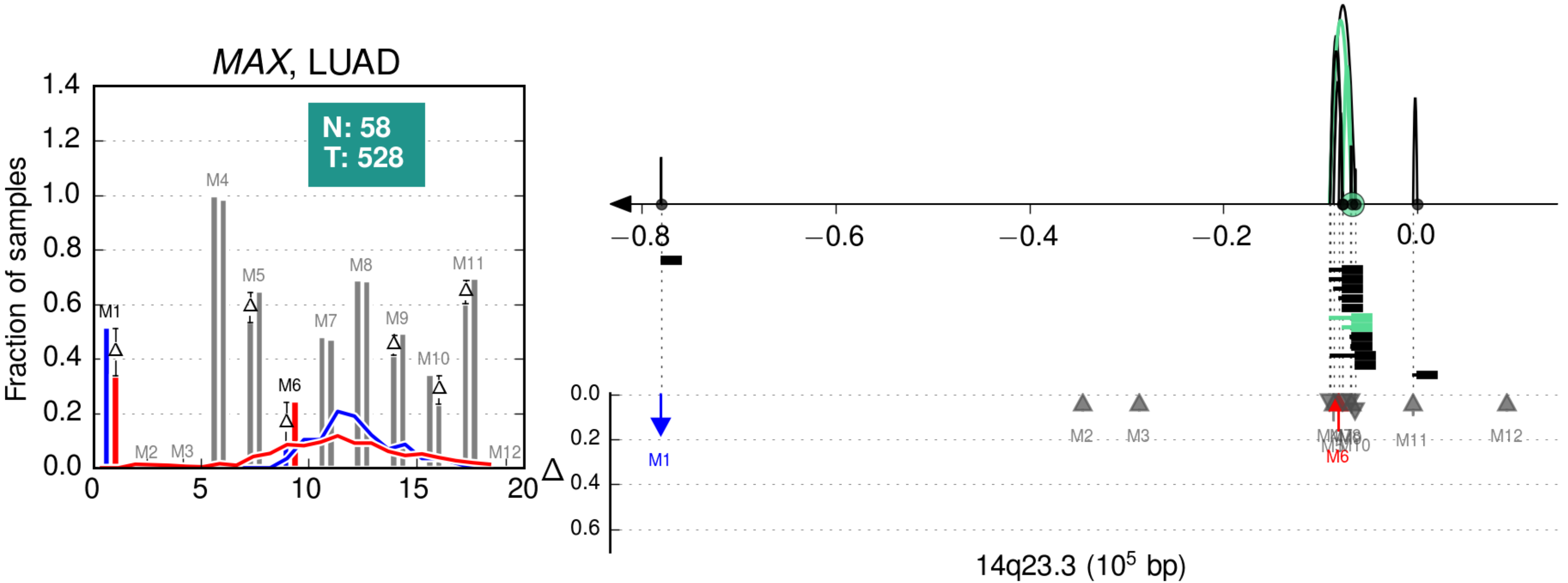

MAX, LUAD
N: 58
T: 528
Fraction of samples
M1
M2
M3
M4
M5
M6
M7
M8
M9
M10
M11
M12
Δ
14q23.3 ($10^5$ bp)

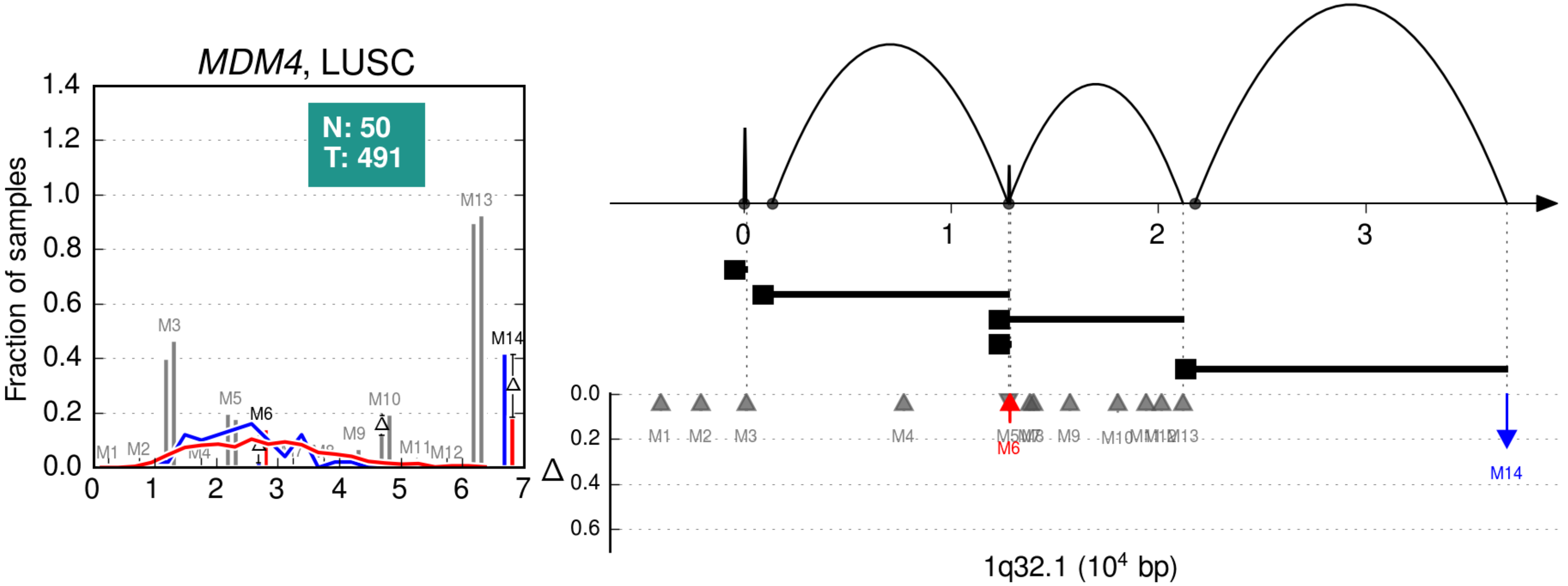

MDM4, LUSC
N: 50
T: 491
Fraction of samples
M1
M2
M3
M4
M5
M6
M9
M10
M11
M12
M13
M14
Δ
1q32.1 (10^4 bp)
M14

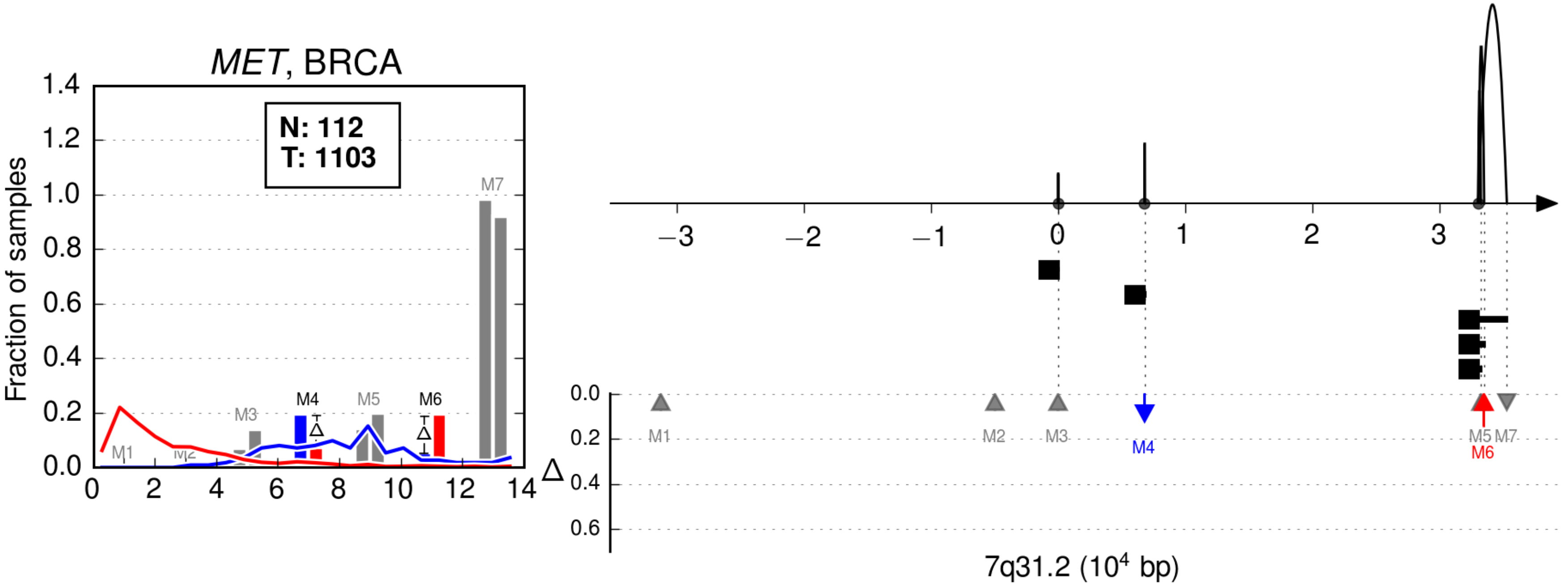

MET, BRCA
N: 112
T: 1103
Fraction of samples
1.4
1.2
1.0
0.8
0.6
0.4
0.2
0.0
0 2 4 6 8 10 12 14
M1
M2
M3
M4
M5
M6
M7
Δ
−3 −2 −1 0 1 2 3
0.0
0.2
0.4
0.6
7q31.2 (10^4 bp)

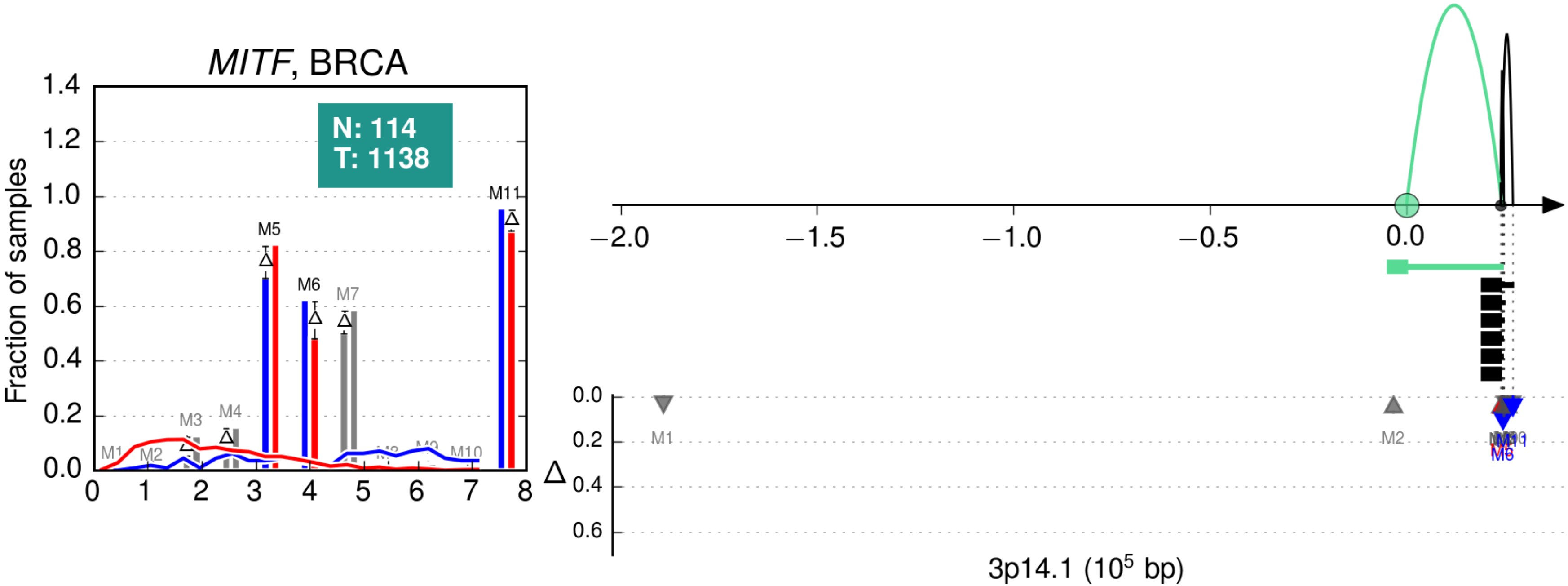

MITF, BRCA
N: 114
T: 1138
Fraction of samples
M1
M2
M3
M4
M5
M6
M7
M10
M11
3p14.1 (10^5 bp)

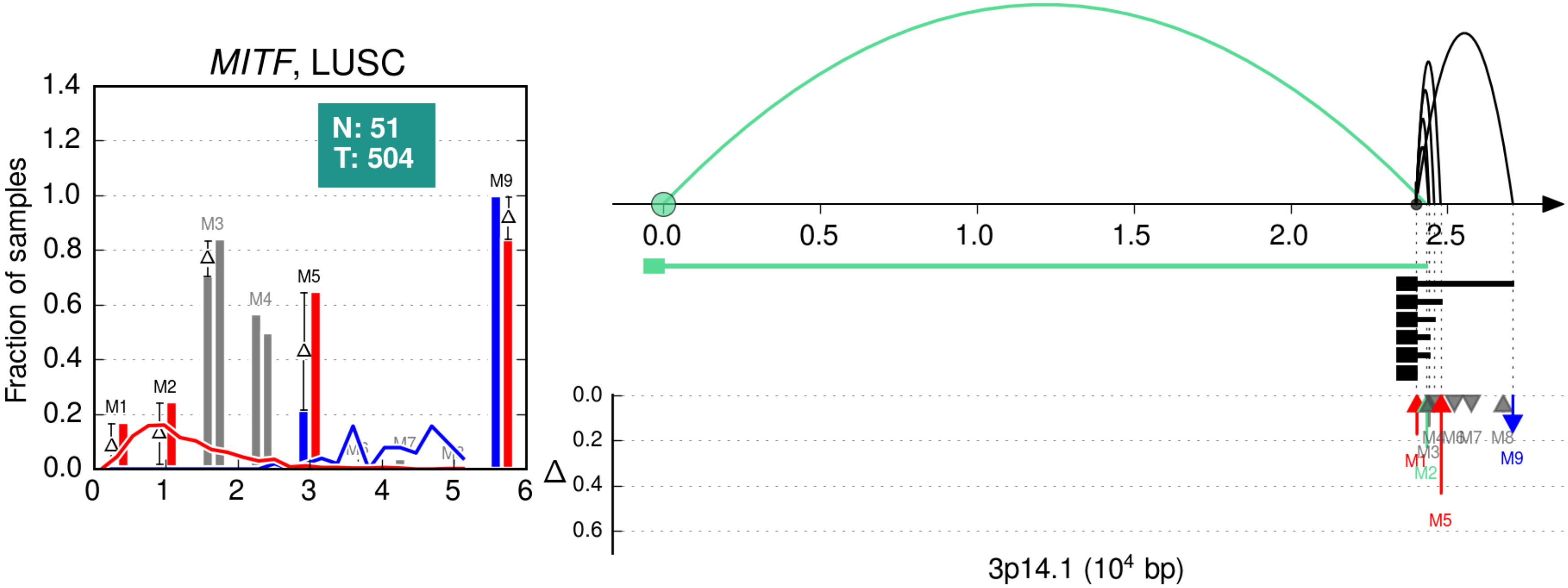

MITF, LUSC
N: 51
T: 504
Fraction of samples
1.4
1.2
1.0
0.8
0.6
0.4
0.2
0.0
0
1
2
3
4
5
6
M1
M2
M3
M4
M5
M7
M9
0.0
0.5
1.0
1.5
2.0
2.5
Δ
0.0
0.2
0.4
0.6
M4
M6
M7
M8
M3
M1
M2
M5
M9
3p14.1 ($10^4$ bp)

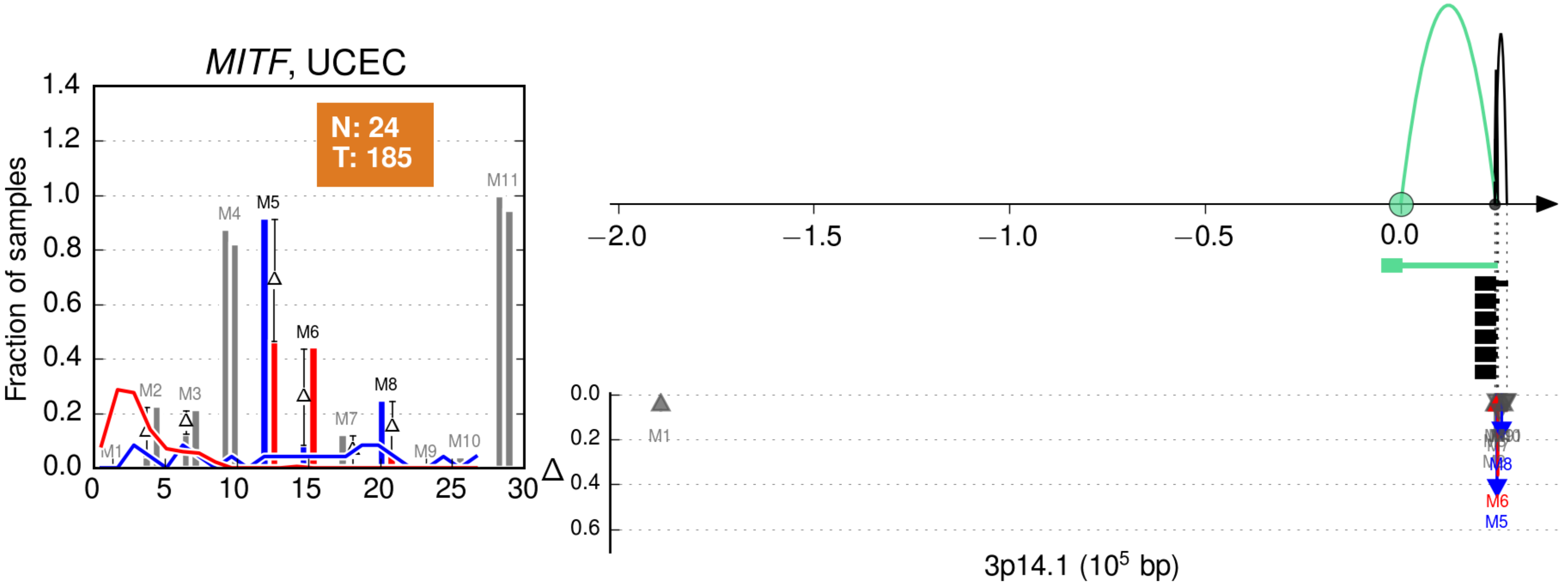

MITF, UCEC
N: 24
T: 185
Fraction of samples
M1
M2
M3
M4
M5
M6
M7
M8
M9
M10
M11
Δ
3p14.1 ($10^5$ bp)
M6
M5

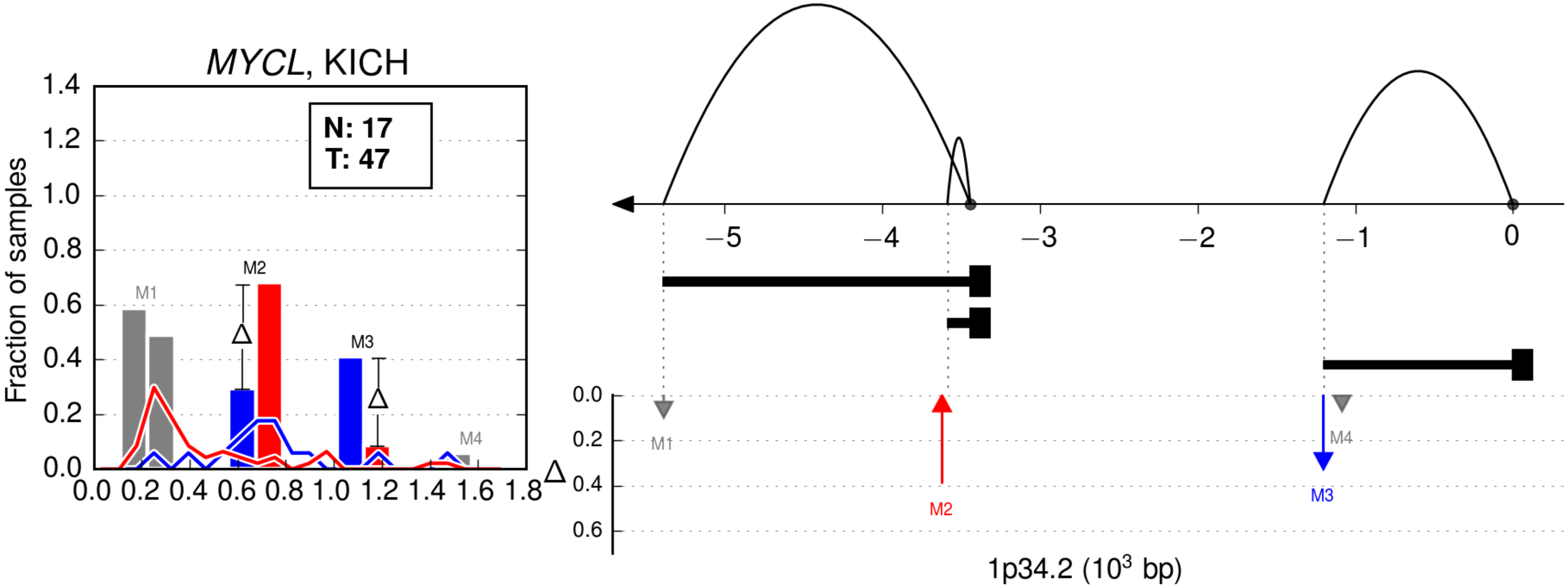

MYCL, KICH
N: 17
T: 47
Fraction of samples
1.4
1.2
1.0
0.8
0.6
0.4
0.2
0.0
0.0 0.2 0.4 0.6 0.8 1.0 1.2 1.4 1.6 1.8
Δ
M1
M2
M3
M4
−5 −4 −3 −2 −1 0
0.0
0.2
0.4
0.6
1p34.2 (10³ bp)

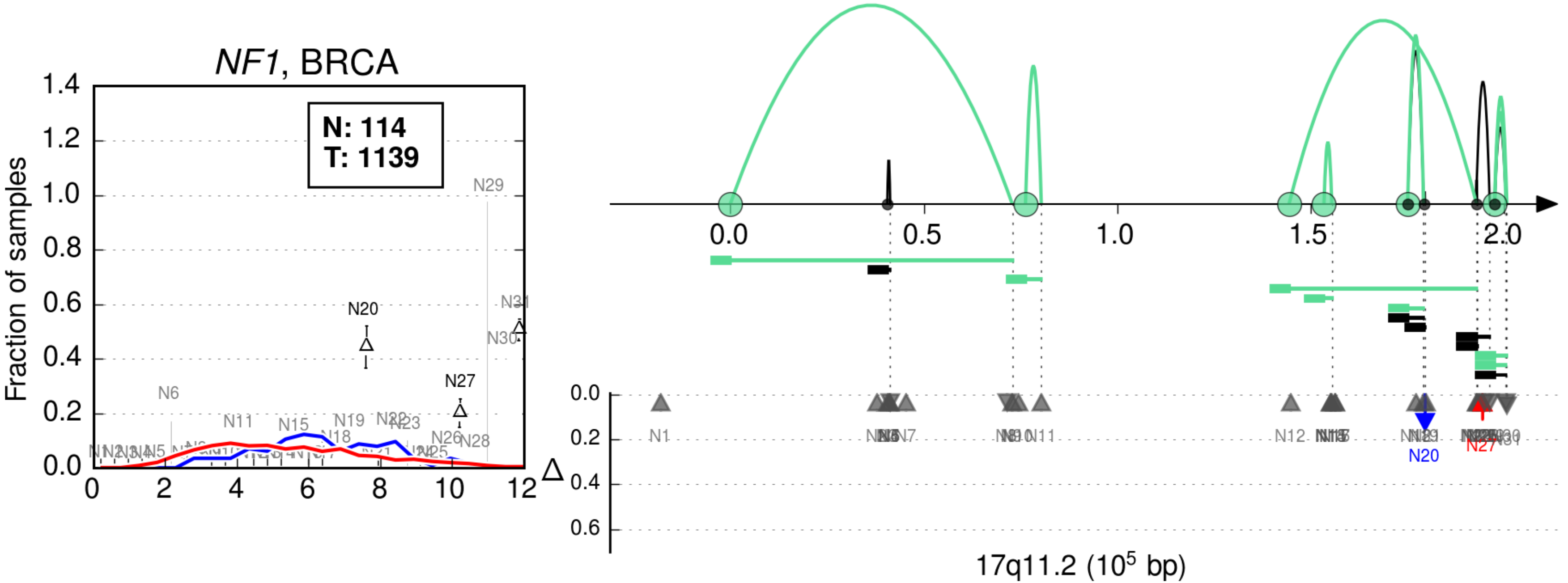
NF1, BRCA
N: 114
T: 1139
Fraction of samples
N20
N27
N29
N31
N30
N6
N11
N15
N19
N22
N23
N26
N28
Δ
17q11.2 ($10^5$ bp)
N1
N7
N11
N12
N20
N27
N31

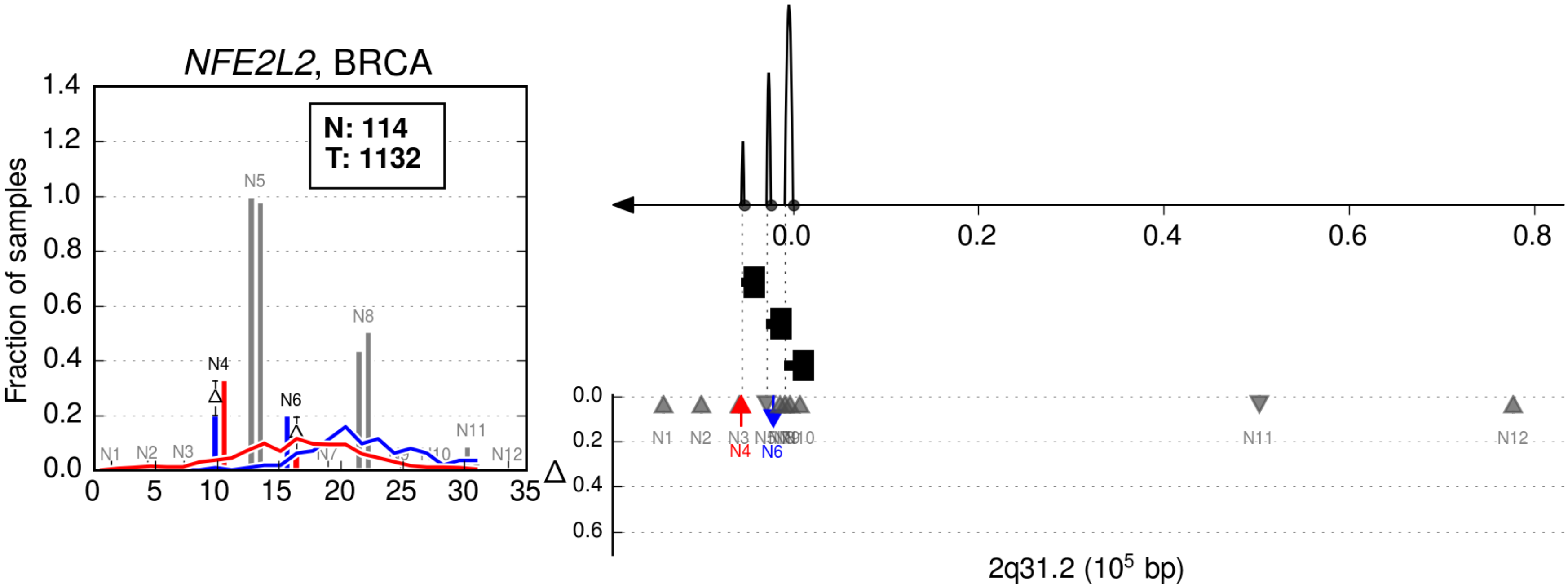
NFE2L2, BRCA
N: 114
T: 1132
Fraction of samples
N1
N2
N3
N4
N5
N6
N7
N8
N11
N12
2q31.2 ($10^5$ bp)
Δ

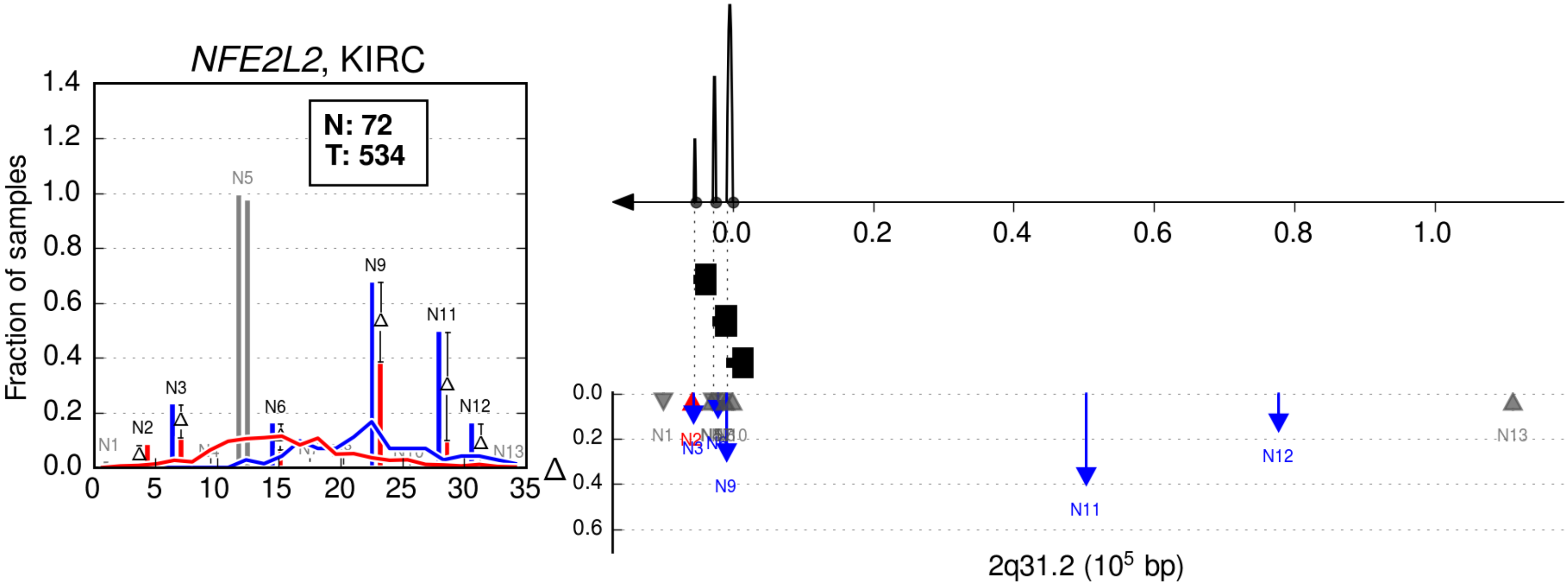

NFE2L2, KIRC
N: 72
T: 534
Fraction of samples
N1
N2
N3
N5
N6
N9
N11
N12
N13
Δ
2q31.2 ($10^5$ bp)
N10

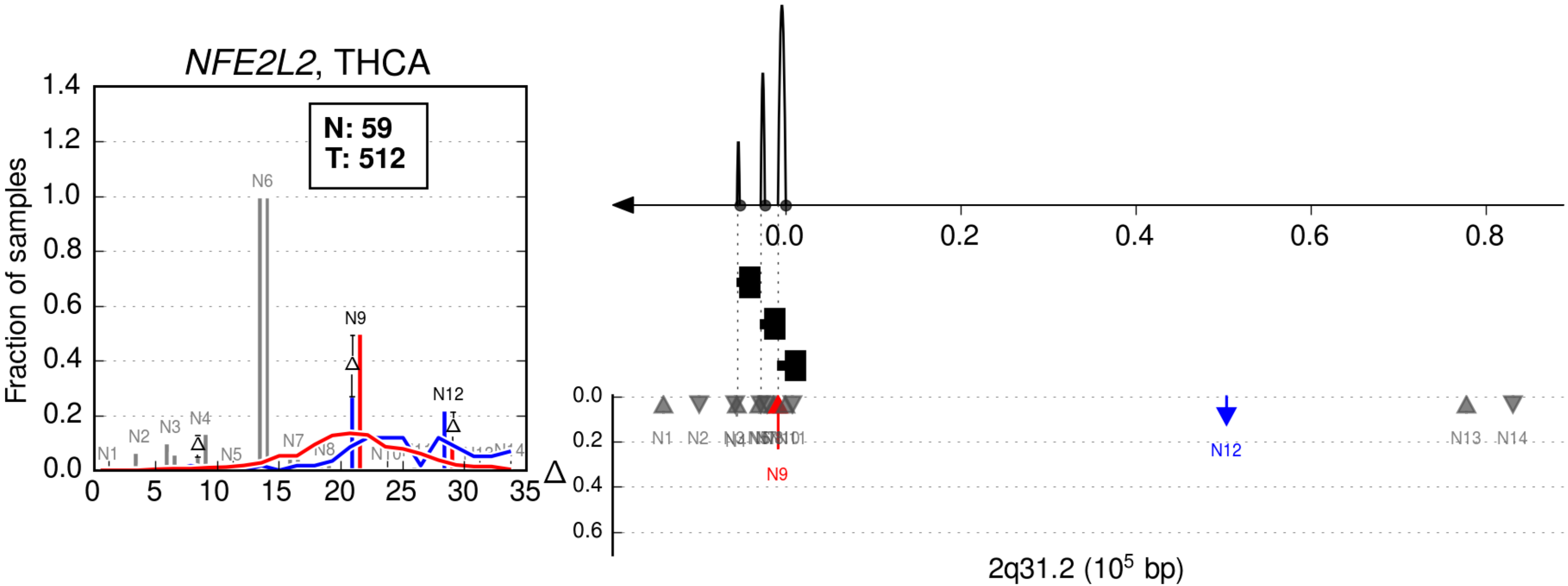
NFE2L2, THCA
N: 59
T: 512
Fraction of samples
N1
N2
N3
N4
N5
N6
N7
N8
N9
N12
Δ
2q31.2 (10^5 bp)
N13
N14

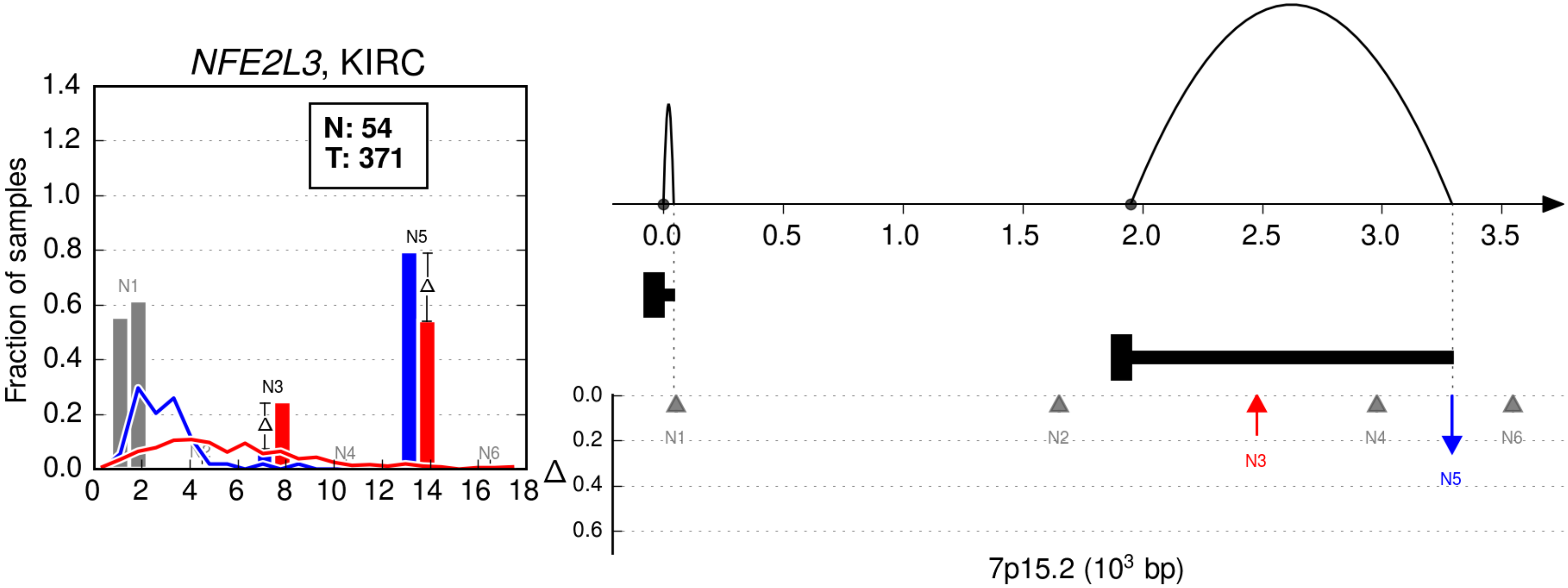
NFE2L3, KIRC
N: 54
T: 371
Fraction of samples
N1
N3
N5
N4
N6
Δ
7p15.2 (10³ bp)
N2

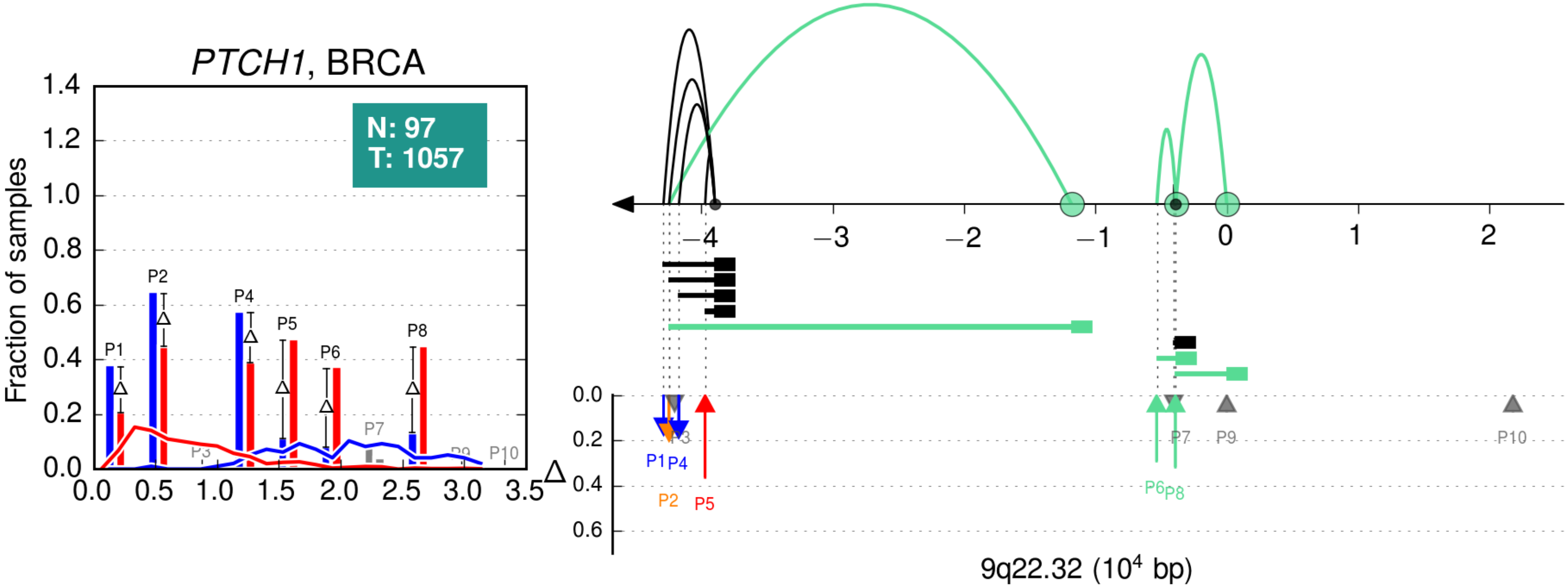

PTCH1, BRCA
N: 97
T: 1057
Fraction of samples
P1
P2
P3
P4
P5
P6
P7
P8
P9
P10
Δ
9q22.32 ($10^4$ bp)

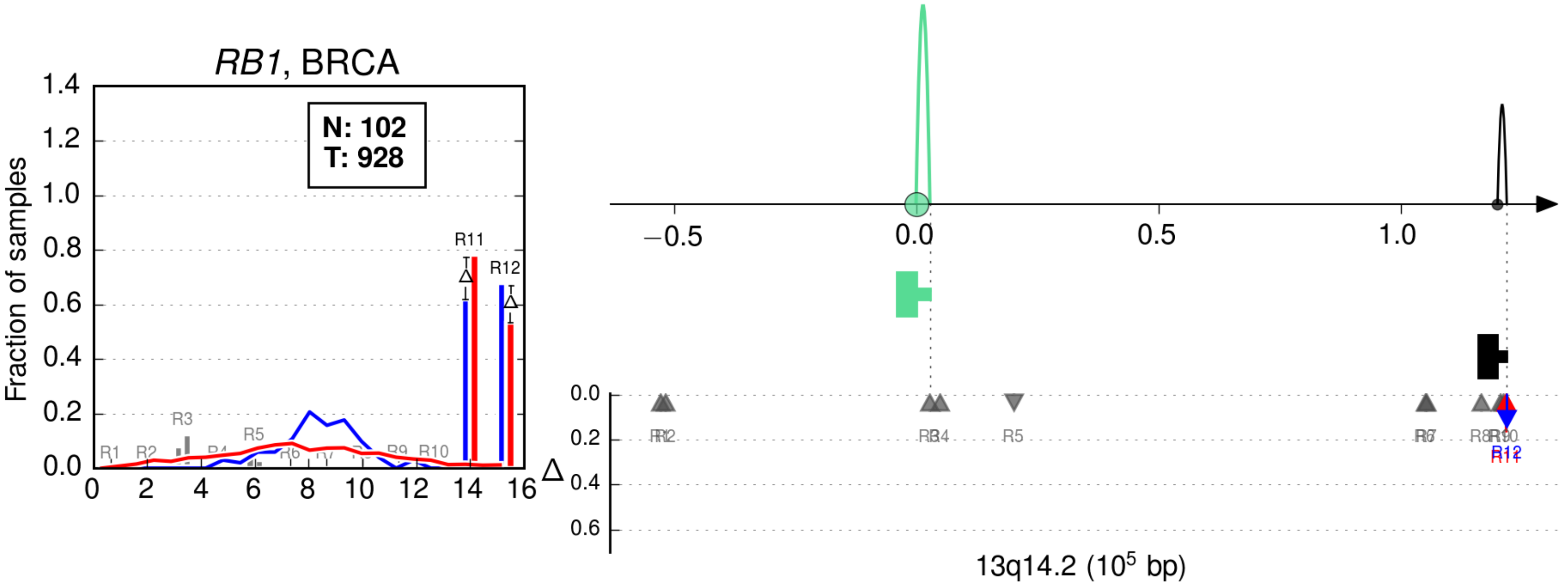

RB1, BRCA
N: 102
T: 928
Fraction of samples
R11
R12
R3
R5
R1
R2
R9
R10
Δ
13q14.2 (10^5 bp)

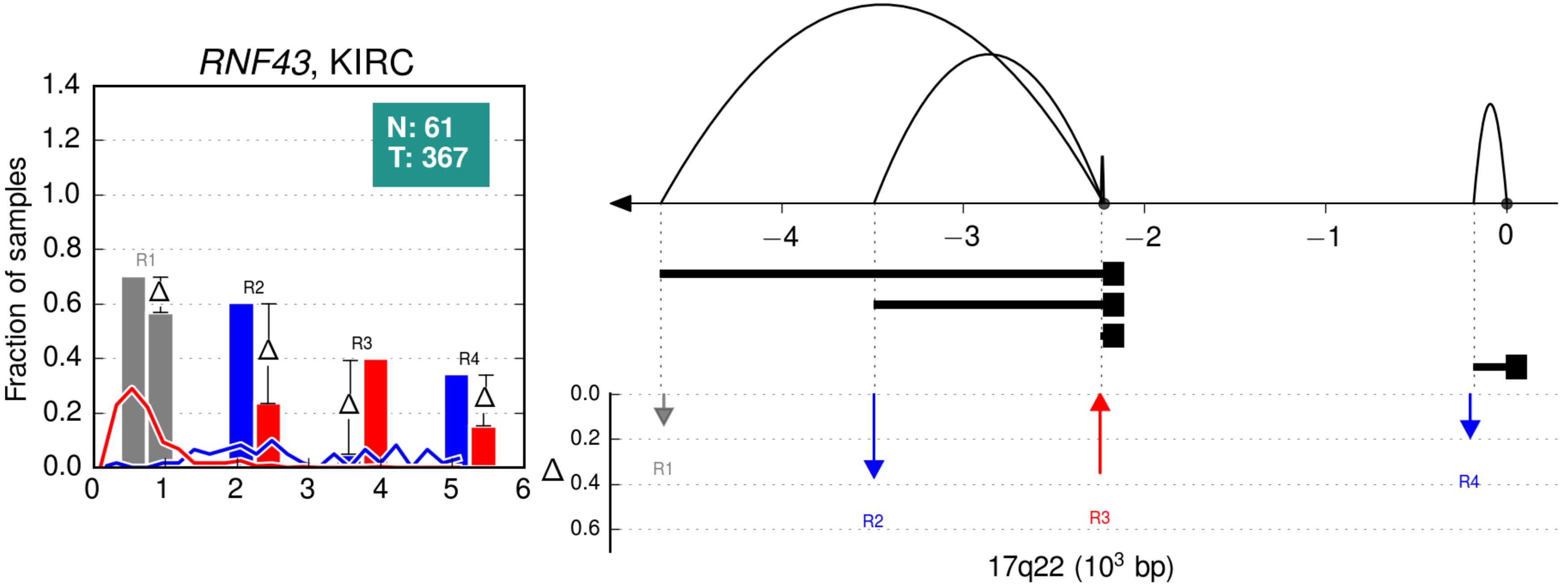

RNF43, KIRC
N: 61
T: 367
Fraction of samples
R1
R2
R3
R4
Δ
17q22 (10³ bp)

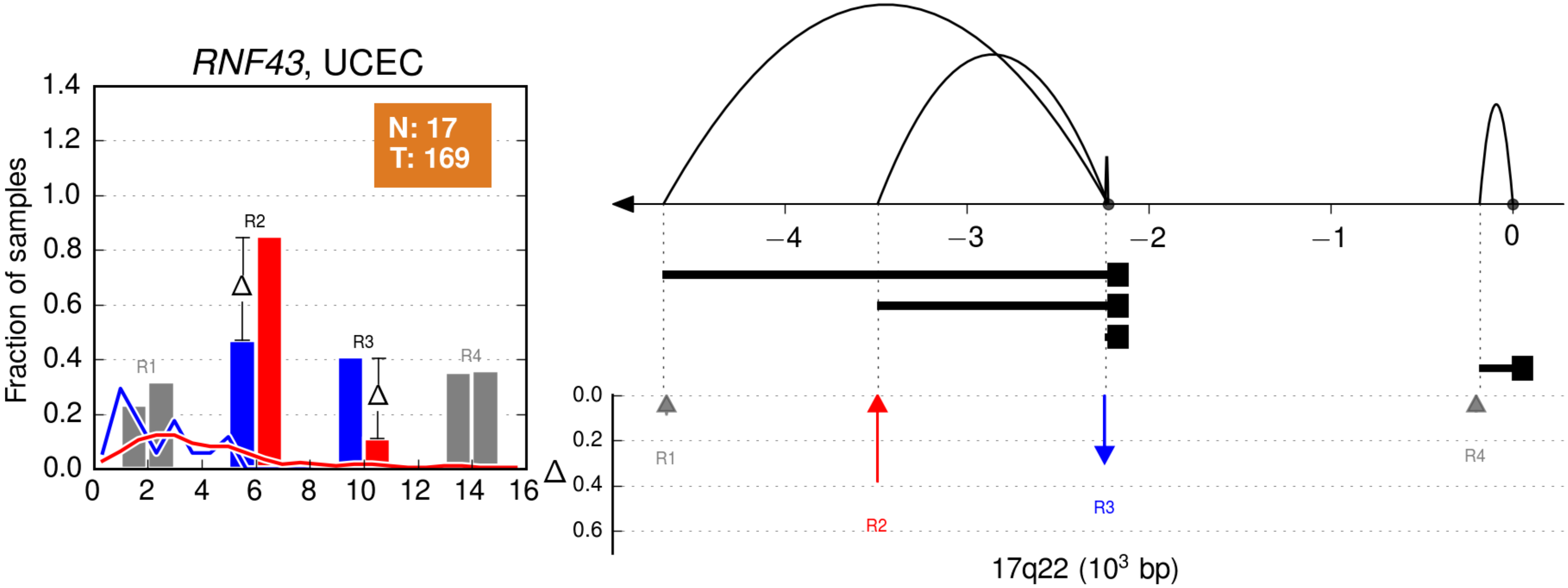
RNF43, UCEC
N: 17
T: 169
Fraction of samples
R1
R2
R3
R4
Δ
17q22 (10³ bp)

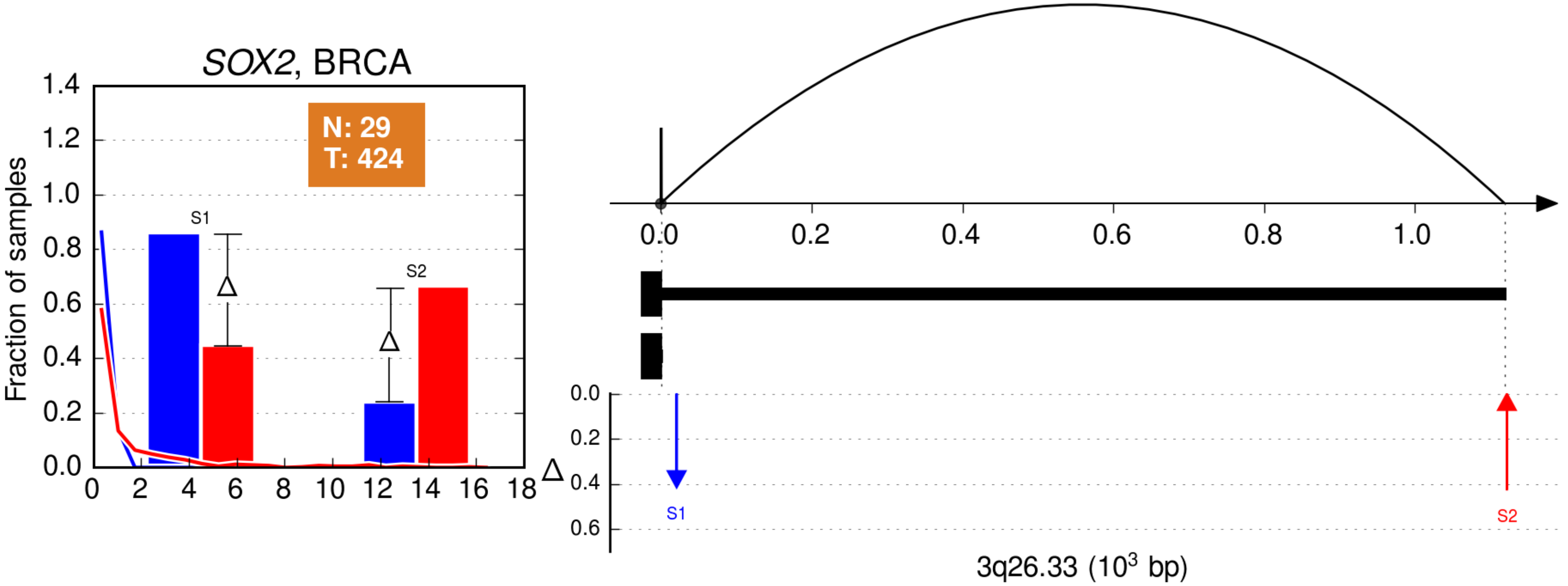

SOX2, BRCA
N: 29
T: 424
Fraction of samples
1.4
1.2
1.0
0.8
0.6
0.4
0.2
0.0
0
2
4
6
8
10
12
14
16
18
S1
S2
Δ
0.0
0.2
0.4
0.6
0.8
1.0
S1
S2
3q26.33 (10^3 bp)

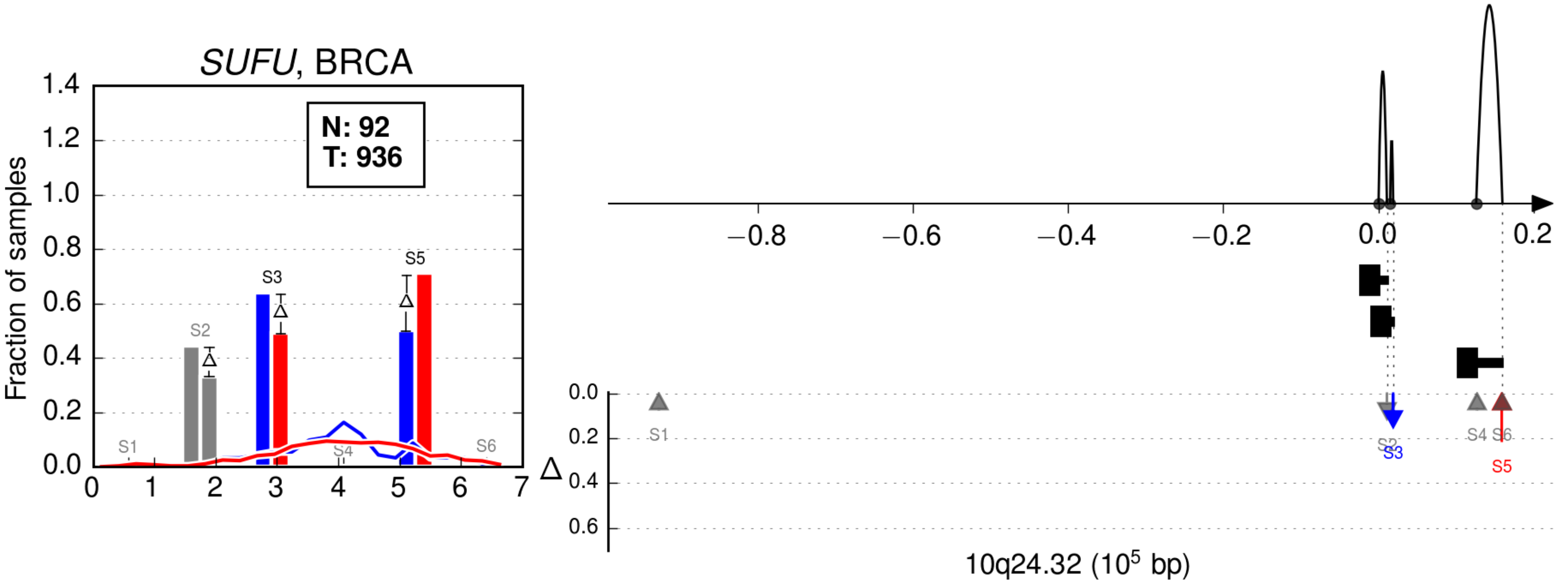

SUFU, BRCA
N: 92
T: 936
Fraction of samples
S1
S2
S3
S4
S5
S6
Δ
10q24.32 (10^5 bp)

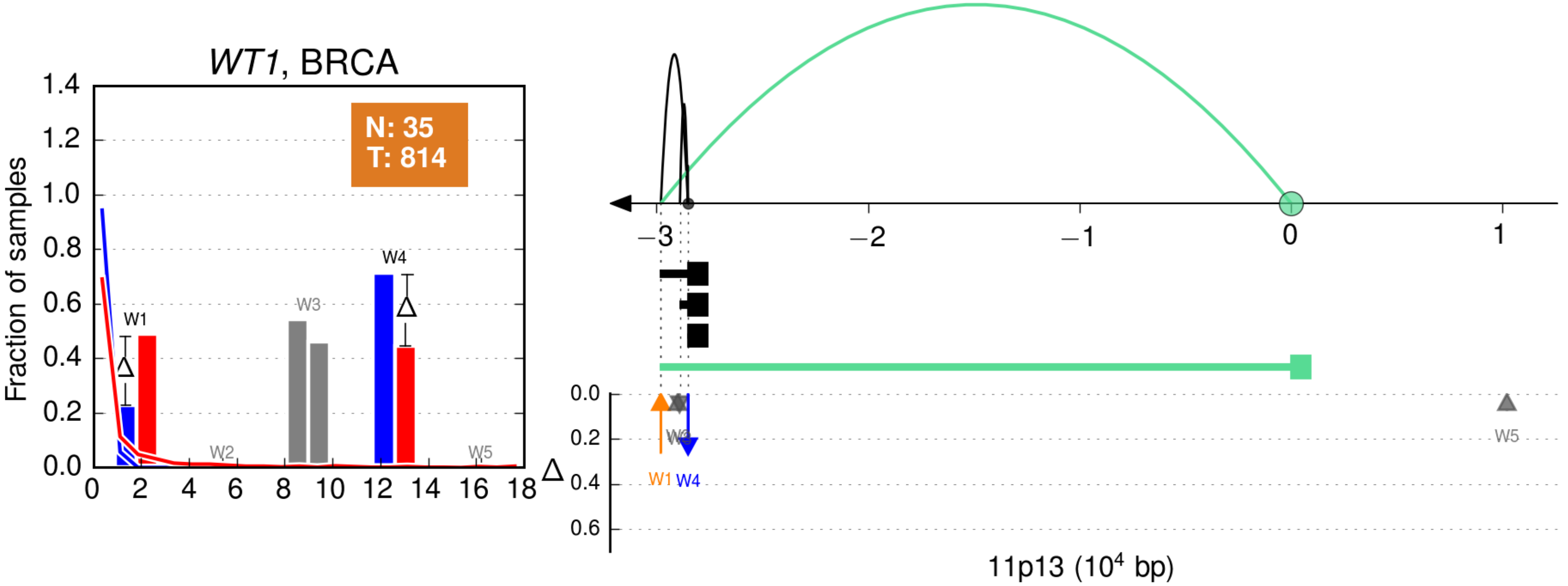

WT1, BRCA
N: 35
T: 814
Fraction of samples
1.4
1.2
1.0
0.8
0.6
0.4
0.2
0.0
0
2
4
6
8
10
12
14
16
18
W1
W2
W3
W4
W5
Δ
−3
−2
−1
0
1
0.0
0.2
0.4
0.6
W1
W4
W5
11p13 ($10^4$ bp)

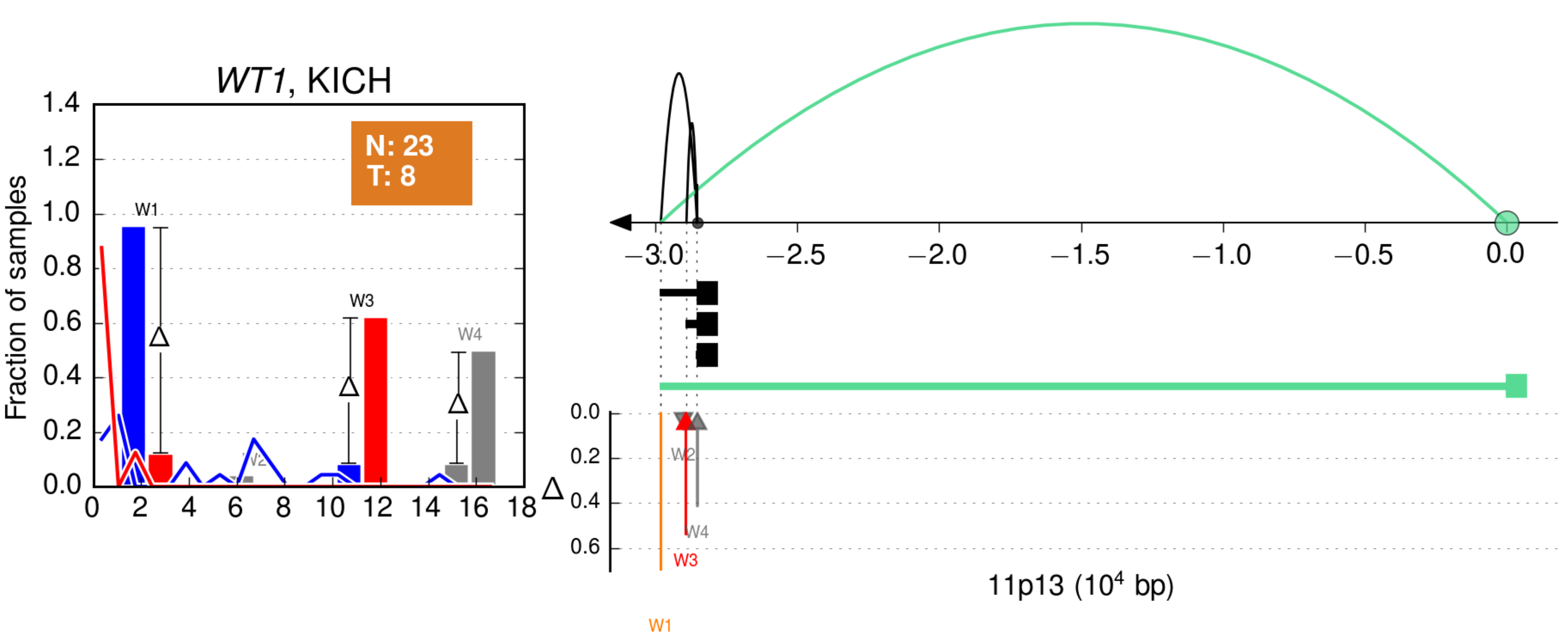

WT1, KICH
N: 23
T: 8
Fraction of samples
W1
W3
W4
W2
Δ
11p13 (10^4 bp)
W1
W2
W3
W4

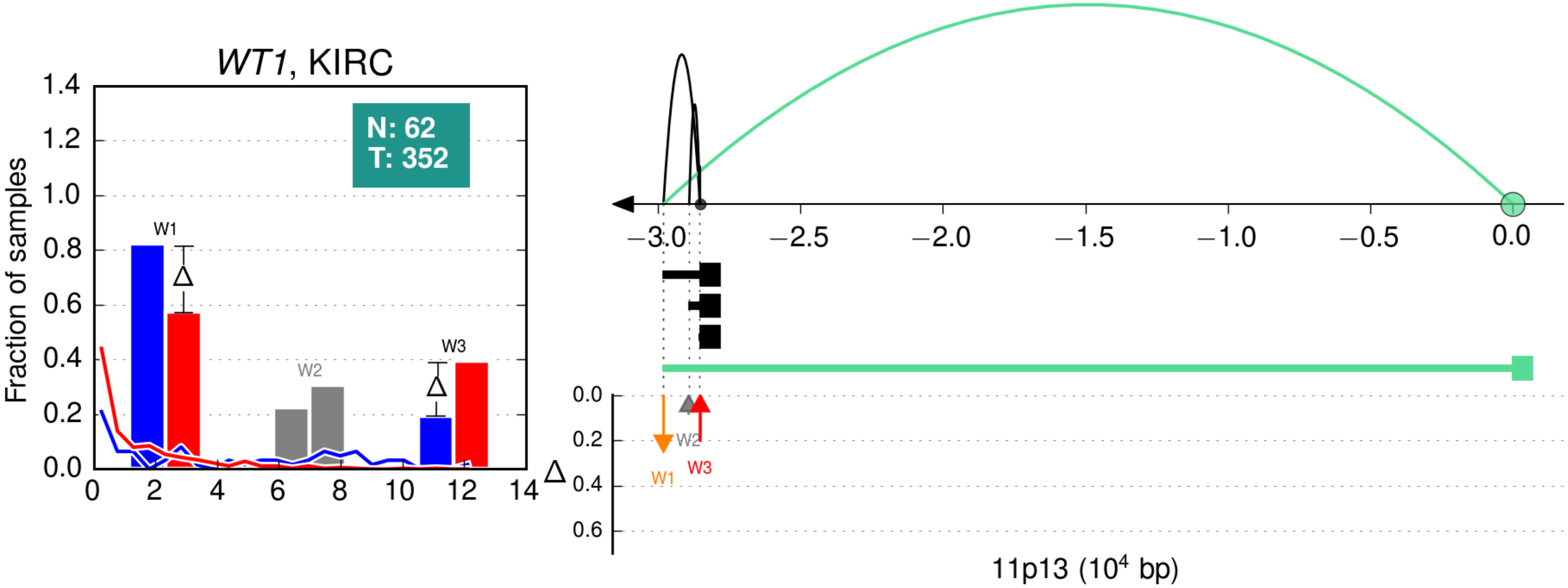

WT1, KIRC
N: 62
T: 352
Fraction of samples
W1
W2
W3
Δ
1.4
1.2
1.0
0.8
0.6
0.4
0.2
0.0
0
2
4
6
8
10
12
14
−3.0
−2.5
−2.0
−1.5
−1.0
−0.5
0.0
0.2
0.4
0.6
11p13 ($10^4$ bp)

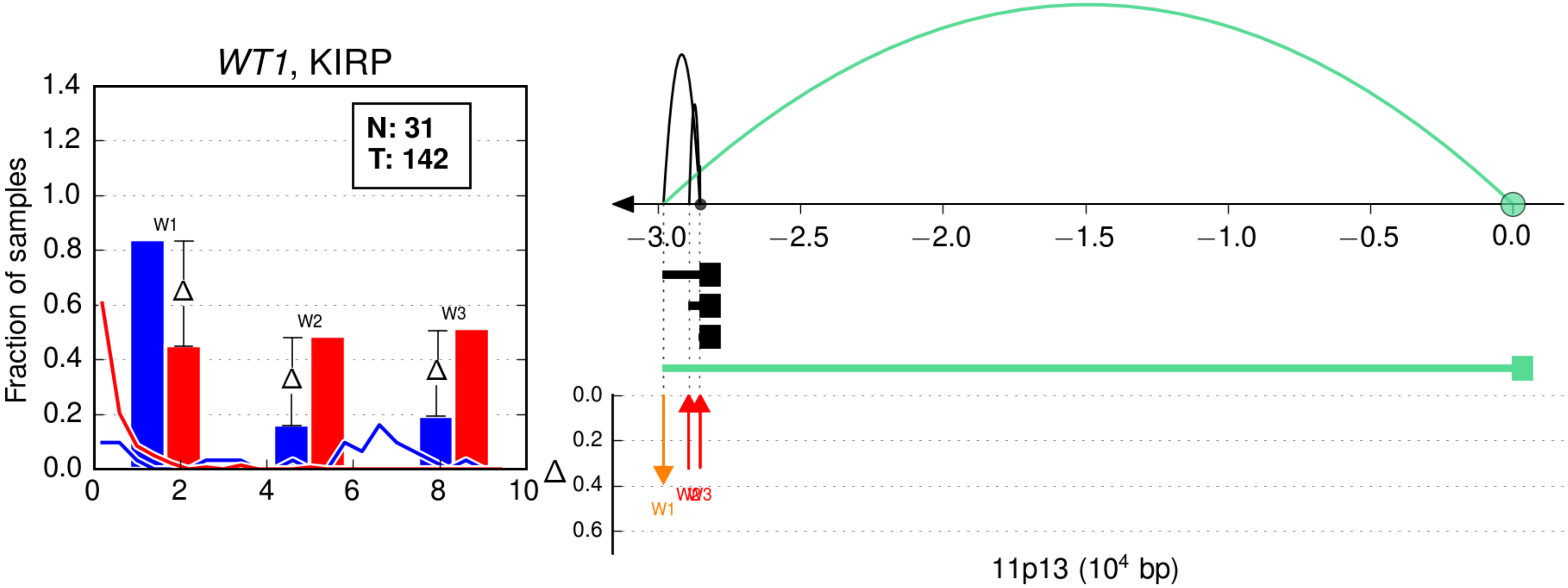

WT1, KIRP
N: 31
T: 142
Fraction of samples
W1
W2
W3
Δ
11p13 ($10^4$ bp)
