# Supplementary material for: Recurrent tumor-specific regulation of alternative polyadenylation of cancer-related genes
Source: BMC Genomics. 2018 Jul 13;19:536. doi: 10.1186/s12864-018-4903-7 (PMC6045855; doi:10.1186/s12864-018-4903-7)
Supplement: Supplementary file 4 — Supplementary methods. Description of additional methods. (PDF 157 kb) [file 12864_2018_4903_MOESM4_ESM.pdf]

Supplementary Methods

Details of sequence data collection and processing

The access to the TCGA RNA-Seq data hosted by ISB-CGC on the GCP was authorized through the ISB-CGC web application (https://isb-cgc.appspot.com/). The data were in both FASTQ and BAM formats. The former format was preferred because some of the BAM records were found to contain a confusing SAM flag value of 195, which means a read is both the first and second read in a pair [1]. There was a total of 11,340 RNA-Seq samples identified by analysis ID, but 672 were excluded from the analysis for the following reasons:

- 648 samples were produced with single-end sequencing method
- 23 samples had inconsistent read length distribution
- One sample (a1d922bb-a14c-48e5-86df-c6e74413955e) did not finish the pipeline analysis due to an error related to non-matching read IDs.

The remaining 10,668 samples finished the pipeline analysis, successfully.

Raw FASTQ reads on the GCS were stored in one archived tar ball per sample. Tar balls from UNC-LCCC and BI were archived first and then compressed with a file extension ".tar.gz" while those from BCCAGSC were compressed first and then archived with an extension of ".tar". Each tar ball from UNC-LCCC and BI had 2 FASTQ files, which contained the first-end and second-end reads, respectively, while that from BCCAGSC could have 2, 4, or 6 FASTQ files. In cases with more than 2 FASTQ files, they were generated on different DNA sequencing instruments, so FASTQ files for the first read and second read were concatenated together. Details on how FASTQ files were extracted and concatenated is found in the *extract_tarball* function in the source code.

Analysis of the relevance of candidate genes to cancer

To generate the entries in Additional file1: Figure S2, we downloaded

1) *CosmicFusionExport.tsv* (sftp://sftp-cancer.sanger.ac.uk//files/grch38/cosmic/v80/CosmicFusionExport.tsv.gz),
2) *CosmicMutantExport.tsv* (sftp://sftp-cancer.sanger.ac.uk//files/grch38/cosmic/v80/CosmicMutantExport.tsv.gz),
3) *CosmicCompleteGeneExpression.tsv* (sftp://sftp-cancer.sanger.ac.uk//files/grch38/cosmic/v80/CosmicCompleteGeneExpression.tsv.gz), and
4) *CosmicSample.tsv* (sftp://sftp-cancer.sanger.ac.uk//files/grch38/cosmic/v80/CosmicSample.tsv.gz)

from COSMIC [2] release v80. Then, we mapped COSMIC diseases to the 33 cancer types in our study based on eight columns (Primary site, Site subtype 1, Site subtype 2, Site subtype 3, Primary histology, Histology subtype 1, Histology subtype 2, and Histology subtype 3) in the *CosmicFusionExport.tsv*, *CosmicMutantExport.tsv*, and *CosmicSample.tsv* files (Additional file 2: Table S5). As for *CosmicCompleteGeneExpression.tsv*, we inner joined it with *CosmicSample.tsv* based on the *sample_name* column to incorporate the disease identity columns. A fusion (F), mutation (M), overexpression (O), and/or underexpression (U) was assigned to a gene in a disease if the gene exhibited a fusion event in the *CosmicFusion.tsv*, a pathogenic (FATHMM [3] score ≥ 0.7) mutation in the *CosmicMutantExport.tsv* file, overexpression, and/or underexpression in the *CosmicCompleteGeneExpression.tsv* file, respectively. We note that COSMIC's definitions of overexpression and underexpression are sample specific, meaning the expression in the sample is more than two standard deviations away from the average expression level of all samples from the same study.

Extraction of mapping information between annotated CSs and stop codons

For a given transcript, usually both of its stop codon and 3' UTR were annotated. Then, the extraction of their coordinates was straightforward. Otherwise, they were inferred as follows:

1) If the 3' UTR was annotated but the stop codon was not, then the coordinate of the stop codon was calculated as the starting position of the 3' UTR – 1 for plus-strand transcript or that + 1 for minus-strand transcript. This happens when a transcript is CDS 3' incomplete or CDS 5' incomplete. Since we ignored the former kind, it only applies to the latter in our analysis (e.g. ENST00000470094).
2) If the stop codon was annotated but the 3' UTR was not, then the end of the transcript was used as the 3' UTR end. This happens both when the corresponding transcript has a 0-length 3' UTR (e.g. ENST00000306120) and not (e.g. ENST00000397332).
3) If neither 3' UTR nor stop codon was annotated, then the transcript was not considered. This happens when a transcript is both CDS 3' incomplete and CDS 5' incomplete. Since we enforced that CDS 3' must be complete, so it didn't apply here.

The coordinate system used is illustrated in Additional file 1: Figure S3B.

Quantification of gene expression

Because we only considered a select list of 114 genes, the total number of reads mapped to the entire transcriptome was irrelevant for expression level normalization. Here, we used RPKMS. A previous study has shown that RPKMS and RPKM are closely correlated when the read quality is high [4], and for the samples analyzed, no poor quality read was found based on the analysis of FastQC files (part of metadata). To calculate RPKMS, we first ran Bedtools [5] *genomecov* to calculate the coverage of each individual base of the genome from mapped RNA-Seq reads. Then, the mean coverage of each gene was calculated as the total number of sequenced nucleotides within its coordinates divided by its effective

length. An effective gene length was defined as the total number of bases whose coverage was at least one. Lastly, the RPKMS of a gene was calculated as the mean coverage normalized by the total number of sequenced nucleotides scaled by 10^9:

$$\text{RPKMS} = \frac{10^9}{\text{totalNumSequencedReads} \cdot \text{readLength}} \cdot \frac{\text{numSequencedNucleotidesWithinAGene}}{\text{effectiveGeneLength}}$$

Trend resolution approach

Inspired by stop codon-level comparison, for each case of tumor-specific cleavage pattern, we first resolved the 3’ UTR shortening or lengthening trend at the stop codon level for every stop codon of a gene, and then integrated the stop-codon-level results to determine a trend at the gene level. The gene-level trend could be shortening, lengthening or complex. Here is the pseudocode:

1. First, regardless of the significance, all frequency changes of all CSs between normal and tumor samples were grouped by stop codon.
2. For each stop codon,
    a. We ordered the frequency changes (up, down or insignificant) from normal to tumor by their corresponding 3’ UTR lengths.
        i. If there were both up and down changes, then insignificant changes were ignored.
        ii. If there were only up and insignificant changes, insignificant ones were considered down changes in relative terms.
        iii. If there were only down and insignificant changes, insignificant ones were considered up changes in relative terms.
    b. If up and down changes were partitioned after sorting by 3’ UTR length:
        i. If there were both up and down:

            1. If shorter 3' UTRs are all associated with the "up" partition, then it's a shortening trend
            2. If shorter 3' UTRs are all associated with the "down" partition, then it's a lengthening trend
        ii. If there was only up or down, then the trend was considered unresolvable and left undefined
    c. If up and down changes were not partitioned, the trend was considered undefined.
3. Then, we integrated all stop codon-level results to determine a trend at the gene-level.
    a. If all stop codon-level trends were undefined, then it was considered a complex trend at the gene-level.
    b. Otherwise,
        i. If all stop codon-level trends contain only shortening and possibly also undefined trend, then the gene-level trend was shortening.
        ii. If all stop codon-level trends contain only lengthening and possibly also undefined trend, then the gene-level trend was lengthening.
        iii. If all stop codon-level trends contain both shortening and lengthening trends, then gene-level trend was considered complex.

Gene annotations used

In this study, two annotation files were used

1) http://ftp.ensembl.org/pub/release-75/gtf/homo_sapiens/Homo_sapiens.GRCh37.75.gtf.gz
2) http://bcgsc.ca/downloads/tasrkleat-static/on-cloud/ensembl.fixed.sorted.gz

The first one was downloaded from Ensembl [6], and used exclusively during the analysis, except when running KLEAT, which used the second file. The second annotation file was originally downloaded from UCSC (http://genome.ucsc.edu/cgi-bin/hgTables) [7]. Gene names in it were considered as gene IDs, and KLEAT [8] was tailored to use this particular version of annotation. We note that the coordinates in the two files also have minor differences, as detailed at https://github.com/bcgsc/tasrkleat-TCGA-analysis-scripts/tree/master/gtf_verification, yet these differences do not impact the conclusions reported.

References

1. Li H, Handsaker B, Wysoker A, Fennell T, Ruan J, Homer N, et al. The Sequence Alignment/Map format and SAMtools. Bioinformatics. 2009;25:2078–9.

2. Forbes SA, Beare D, Gunasekaran P, Leung K, Bindal N, Boutselakis H, et al. COSMIC: exploring the world's knowledge of somatic mutations in human cancer. Nucleic Acids Res. 2015;43:D805–11.

3. Shihab H a, Rogers MF, Gough J, Mort M, Cooper DN, Day INM, et al. An integrative approach to predicting the functional effects of non-coding and coding sequence variation. Bioinformatics. 2015;31:1536–43.

4. Gibb EA, Warren RL, Wilson GW, Brown SD, Robertson GA, Morin GB, et al. Activation of an endogenous retrovirus-associated long non-coding RNA in human adenocarcinoma. Genome Med. 2015;7:22.

5. Quinlan AR, Hall IM. BEDTools: A flexible suite of utilities for comparing genomic features. Bioinformatics. 2010;26:841–2.

6. Aken BL, Ayling S, Barrell D, Clarke L, Curwen V, Fairley S, et al. The Ensembl gene annotation system. Database. 2016;2016:baw093.

7. Rosenbloom KR, Armstrong J, Barber GP, Casper J, Clawson H, Diekhans M, et al. The UCSC Genome Browser database: 2015 update. Nucleic Acids Res. 2015;43:D670–81.

8. Birol I, Raymond A, Chiu R, Nip KM, Jackman SD, Kreitzman M, et al. Kleat: cleavage site analysis of transcriptomes. Pac. Symp. Biocomput. WORLD SCIENTIFIC; 2015;347–58.
