# Supplementary figures and images for: Recurrent tumor-specific regulation of alternative polyadenylation of cancer-related genes
Source: BMC Genomics. 2018 Jul 13;19:536. doi: 10.1186/s12864-018-4903-7 (PMC6045855; doi:10.1186/s12864-018-4903-7)

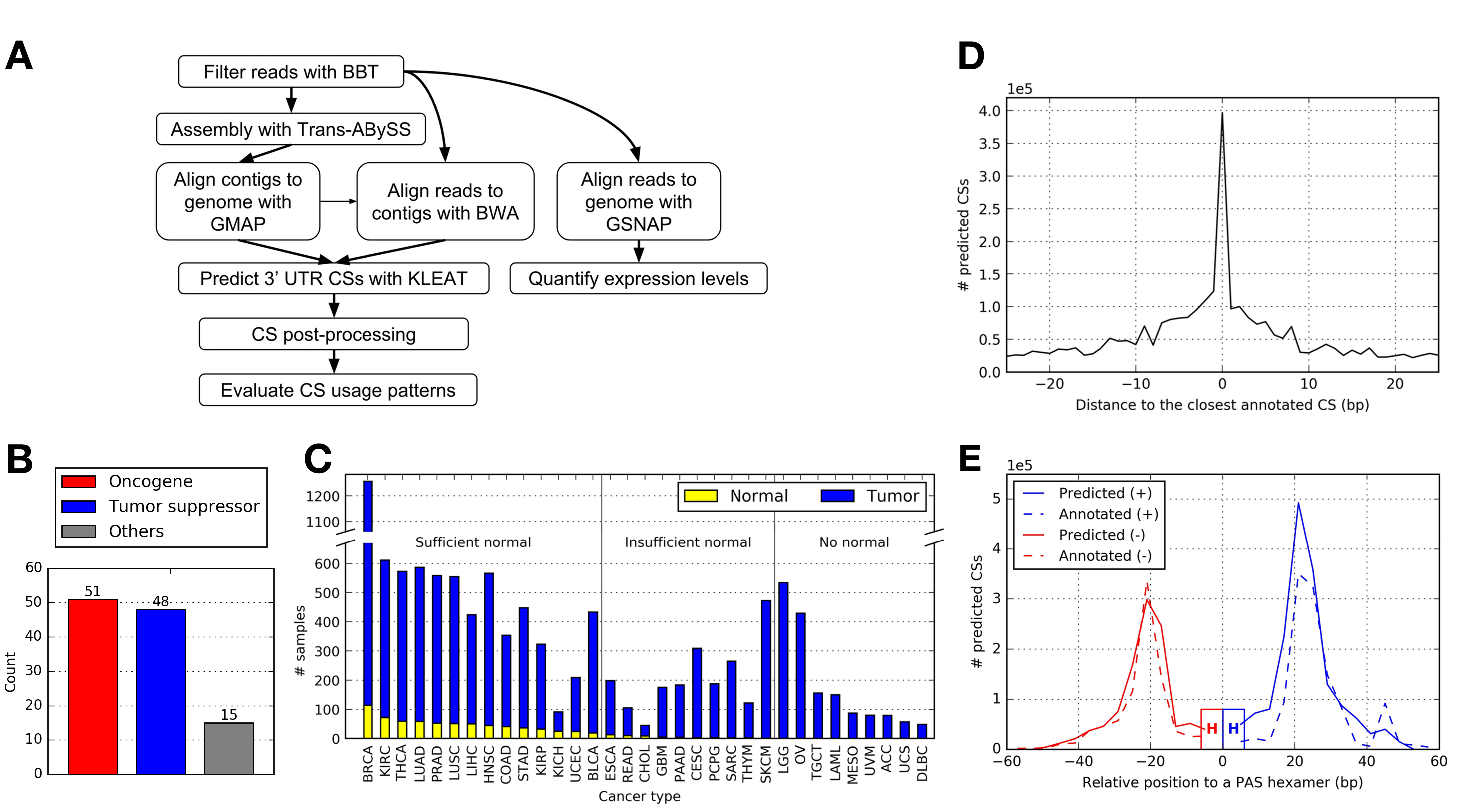

Supplement: Supplementary file 5 — Figures available for download. (PDF 86 kb) [file 12864_2018_4903_MOESM5_ESM.zip › Figure 1.tiff]

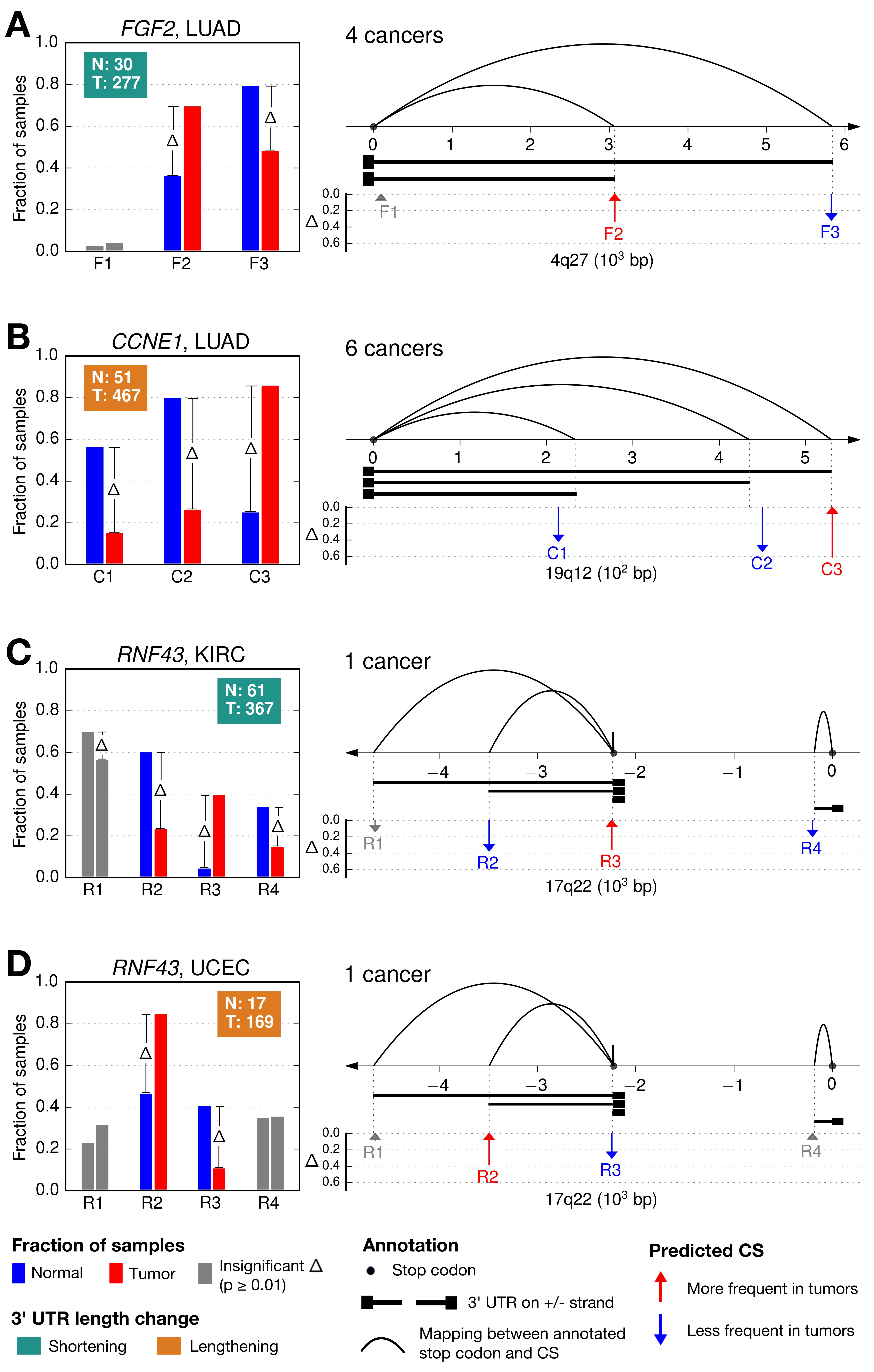

Supplement: Supplementary file 5 — Figures available for download. (PDF 86 kb) [file 12864_2018_4903_MOESM5_ESM.zip › Figure 2.tiff]

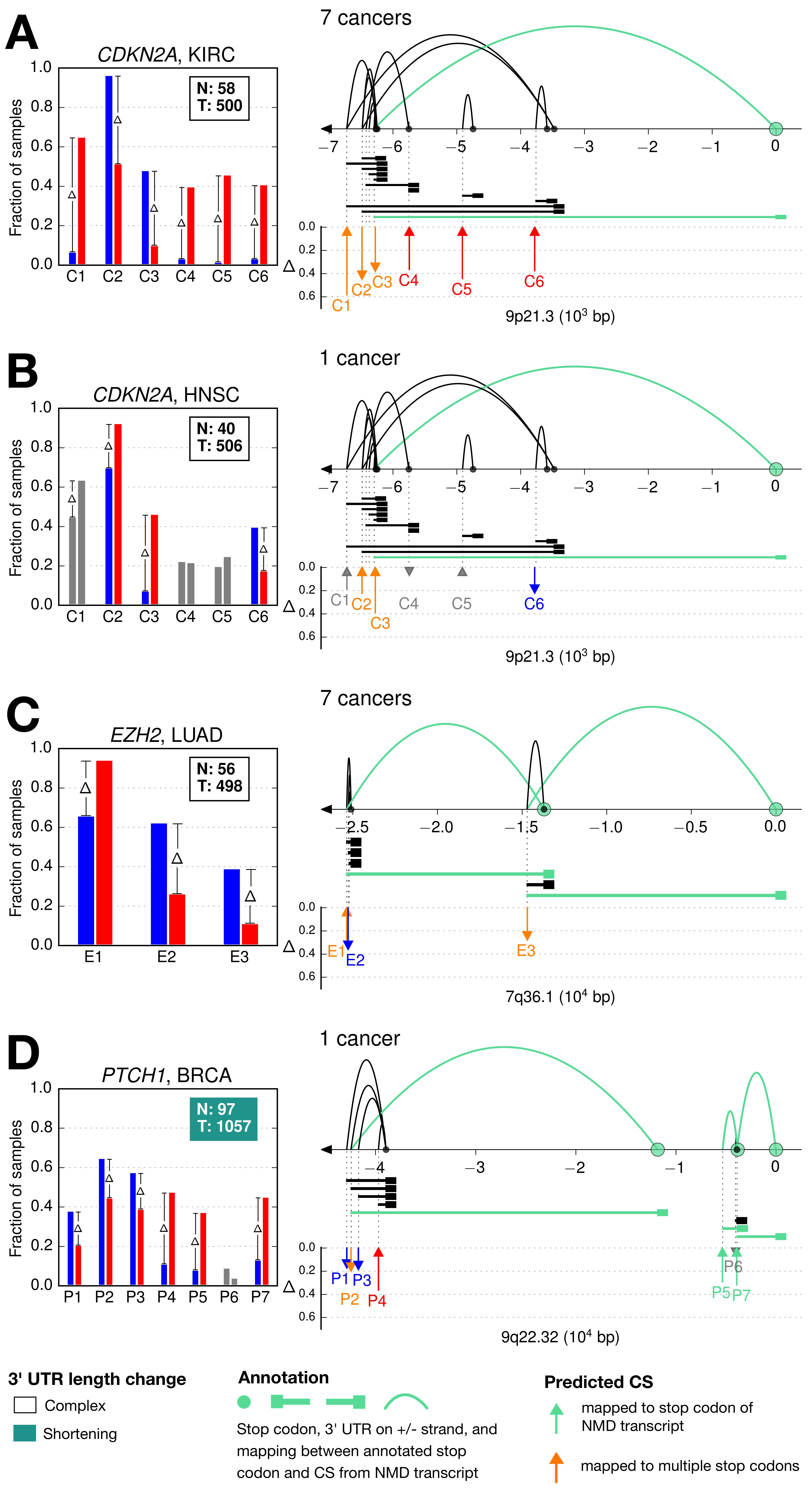

Supplement: Supplementary file 5 — Figures available for download. (PDF 86 kb) [file 12864_2018_4903_MOESM5_ESM.zip › Figure 3.tiff]
